# Polymerase-tagged respiratory syncytial virus reveals a dynamic rearrangement of the ribonucleocapsid complex during infection

**DOI:** 10.1371/journal.ppat.1008987

**Published:** 2020-10-08

**Authors:** Emmeline L. Blanchard, Molly R. Braun, Aaron W. Lifland, Barbara Ludeke, Sarah L. Noton, Daryll Vanover, Chiara Zurla, Rachel Fearns, Philip J. Santangelo

**Affiliations:** 1 Wallace H. Coulter Department of Biomedical Engineering, Georgia Institute of Technology and Emory University, Atlanta, GA, United States of America; 2 Department of Microbiology, Boston University School of Medicine, Boston, MA, United States of America; 3 Institute of Bioengineering and Bioscience, Georgia Institute of Technology, Atlanta, GA, United States of America; Washington University in Saint Louis School of Medicine, UNITED STATES

## Abstract

The ribonucleocapsid complex of respiratory syncytial virus (RSV) is responsible for both viral mRNA transcription and viral replication during infection, though little is known about how this dual function is achieved. Here, we report the use of a recombinant RSV virus with a FLAG-tagged large polymerase protein, L, to characterize and localize RSV ribonucleocapsid structures during the early and late stages of viral infection. Through proximity ligation assays and super-resolution microscopy, viral RNA and proteins in the ribonucleocapsid complex were revealed to dynamically rearrange over time, particularly between 6 and 8 hours post infection, suggesting a connection between the ribonucleocapsid structure and its function. The timing of ribonucleocapsid rearrangement corresponded with an increase in RSV genome RNA accumulation, indicating that this rearrangement is likely involved with the onset of RNA replication and secondary transcription. Additionally, early overexpression of RSV M2-2 from *in vitro* transcribed mRNA was shown to inhibit virus infection by rearranging the ribonucleocapsid complex. Collectively, these results detail a critical understanding into the localization and activity of RSV L and the ribonucleocapsid complex during RSV infection.

## Author summary

Respiratory syncytial virus is a common respiratory disease among children under the age of five. Yearly, approximately three million children infected require hospitalization for treatment. However, no vaccine or effective antiviral treatment for respiratory syncytial virus yet exists. Understanding how respiratory syncytial virus replicates in cells will provide key knowledge for the development of more effective countermeasures. Here, we investigated the interactions between the large protein subunit of the respiratory syncytial virus polymerase, which is responsible for viral mRNA synthesis and replication, and other viral proteins during infection, as well as the localization of the large protein subunit over time in key viral structures. Our study provides new insights into the localization and activity of the respiratory syncytial virus polymerase, and suggests ribonucleocapsid complex rearrangement plays a key role in the function of the respiratory syncytial virus polymerase in replication of the virus, leading to a better understanding of respiratory syncytial virus biology.

## Introduction

Respiratory syncytial virus (RSV) is the leading cause of acute lower respiratory infections in children under the age of five worldwide, leading to roughly three million hospitalizations per year [1]. Reinfection with RSV is common, as infants do not typically generate a strong, lasting immunity to RSV [2,3]. Despite the prevalence of RSV, no vaccine for RSV currently exists and current antiviral treatments are neither highly effective nor often used [2,3]. Development of improved therapies and preventative care for RSV is reliant on a stronger fundamental understanding of RSV biology and pathogenesis.

RSV is an enveloped, negative-sense, single-stranded RNA virus of the family *Pneumoviridae* [2,4]. The virus particle contains the viral ribonucleocapsid, which is comprised of the viral genome, encapsidated along its length with nucleoprotein (N) to form a helical structure, and associated with the viral polymerase, a complex of the large polymerase subunit (L) and phosphoprotein (P), and a transcription elongation protein, M2-1 [5–7]. The ribonucleocapsid is surrounded by matrix protein and a lipid envelope containing the viral glycoproteins, including the fusion protein (F) [8,9]. Fusion of the virus particle with the cell membrane releases the ribonucleocapsid into the cytoplasm, where viral transcription and genome replication occur [9]. Transcription of the genome yields capped and polyadenylated mRNAs, allowing accumulation of viral proteins [2,10]. Following primary transcription, the ribonucleocapsid can begin genome replication [2,9]. This involves synthesis of antigenome RNA, a complete positive sense complement of the genome, which then acts as a template for synthesis of viral genomes [2,9]. These genome RNAs can then serve as templates for secondary transcription and further rounds of RNA replication [2,9]. Ribonucleocapsids are detected in cytoplasmic inclusions, referred to as inclusion bodies (IB) that are thought to be sites of RNA synthesis [10–12]. IBs are spherical structures, roughly 1–5 μm, demarcated by N, P and M2-1 [12]. Newly synthesized genomes are assembled into infectious virions entitled filaments, either during trafficking within the cytoplasm or at the cell membrane [9,13,14]. Filaments contain all RSV proteins in addition to genome and extend from the cells before their release, up to 12 μm in length [15,16].

The mechanism regulating genome-containing ribonucleocapsids between synthesis of mRNAs versus antigenome is not fully understood. Both processes are initiated by the L-P complex from a common promoter at the 3´ end of the genome [17–19]. The polymerase initiates transcription and antigenome synthesis from two distinct sites within the same promoter, and the relative concentrations of the respective initiating NTPs can determine which initiation site the polymerase selects [20]. This, coupled with the fact that the polymerase can only engage in antigenome synthesis following accumulation of sufficient soluble N protein to encapsidate the antigenome, provides an explanation for how a polymerase molecule engaging with the genome template can become committed to either mRNA transcription versus antigenome production [21], but how transcription and replication are temporally controlled remains unclear. In other non-segmented negative strand RNA viruses, increasing concentrations of N protein can elicit a switch from transcription to replication, but this is not the case for RSV [21–24]. However, another factor might be involved. Unlike other non-segmented negative strand RNA viruses, pneumoviruses code for a protein referred to as M2-2, the product of the second open reading frame of the M2 gene. Studies using recombinant RSV showed that ablation of the M2-2 open reading frame resulted in a decrease in RSV genomic RNA and an increase in RSV mRNAs, indicating that M2-2 influences the balance between transcription and genome replication [25].

Nucleocapsids of RSV and other related viruses, such as vesicular stomatitis virus (VSV), Sendai virus, measles virus, mumps virus, and Nipah virus, have conformational flexibility [26–31], which can be affected by interactions with other proteins and salt concentrations [26,27,29]. It has previously been suggested that conformational changes in the paramyxovirus ribonucleocapsid enable it to perform its different functions of transcription versus replication [28], but this possibility has not previously been examined. In work described here, we took advantage of cutting edge imaging methods to examine the distributions of the polymerase and other ribonucleocapsid proteins and RNAs relative to one another at different times post infection to gain a greater understanding of RSV ribonucleocapsid structure and function. A proximity ligation assay (PLA) was used to examine the spatial relationships between proteins and RNA [8,12,32,33]. PLA results in a signal, detectable by microscopy, only when two molecules of interest are proximal to each other. This allows for quantification of protein and RNA interactions on a per-cell basis, providing a measure of the breadth of viral heterogeneity in a population of infected cells [12]. In addition, we utilized super-resolution microscopy, specifically direct stochastic optical reconstruction microscopy (dSTORM), allowing for a close visualization of protein arrangements within the ribonucleocapsid complex [34]. Using these approaches, we showed that the RSV ribonucleocapsid complex undergoes a significant structural rearrangement between 6 and 8 hpi, which correlates with the onset of RNA replication. Further, we demonstrate that over-expression of M2-2 protein early during the infection causes a similar structural rearrangement of nucleocapsids and an inhibition of viral gene expression. These results comprise a new understanding into the dynamics of the ribonucleocapsid complex during RSV infection and reveal that M2-2 plays a role in this process.

## Results

### Generation and characterization of recombinant RSV expressing epitope-tagged L protein

The RSV L protein is the polymerase subunit that contains the enzymatic domains required for transcription and genome replication and so is a key component of ribonucleocapsids [4,18]. It is only expressed at very low levels in infected cells, making it difficult to detect [9,10]. Although a polyclonal antibody specific to L protein has been generated in the past, this antibody did not readily detect L in all inclusion bodies, suggesting that it was not sufficiently sensitive to detect low levels of L protein [10]. Therefore, to allow more efficient detection of L protein in RSV infected cells, a recombinant virus was engineered to express L containing a 2x FLAG tag (S1 Fig) within a hinge region of the polymerase [35]. The 2x FLAG tagged RSV L virus, termed rRSVflag2-L, produced a similar pattern of genomic RNA and mRNA as recombinant RSV lacking a tag (rRSV) (S1A Fig) and Lflag2 was successfully detected via FLAG antibody by western blot (S1B Fig). Importantly, rRSVflag2-L displayed similar growth kinetics as rRSV (S1C Fig). These data indicate that recombinant RSV containing a FLAG tag within the L protein is suitable to study L and the ribonucleocapsid complex.

### Localization of L protein in RSV viral structures during infection

**Fig 1:**
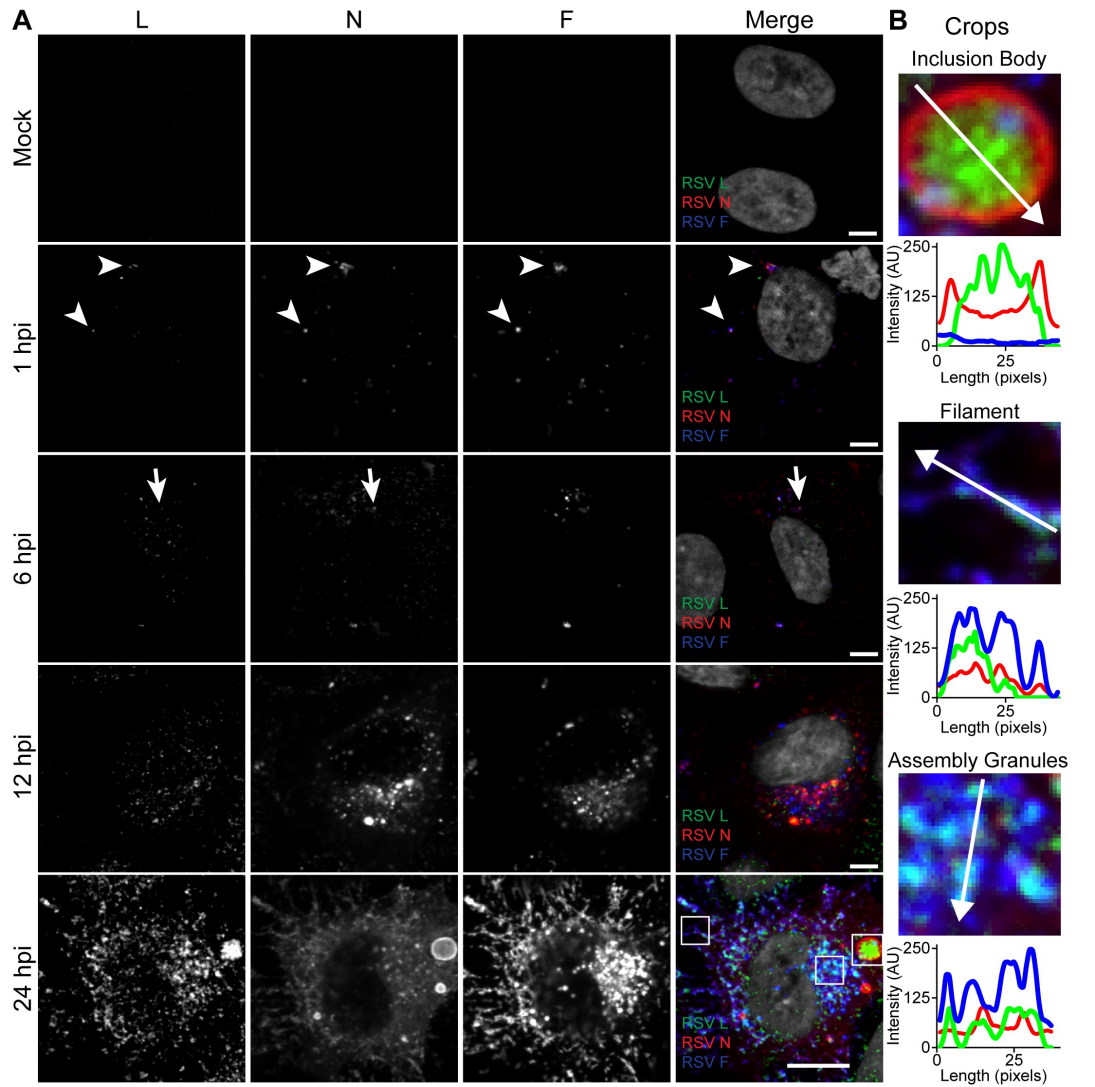
RSV L localizes to inclusion bodies, filaments and assembly granules. **A)** A549 cells were infected or mock infected with rRSVflag(2)L at a MOI of 3. Cells were fixed at 1, 6, 12, and 24 hours post infection. Cells were stained for RSV L (green), RSV N (red) and RSV F (blue). Representative single plane images are shown. Scale bar is 10 μm. Arrowheads indicate virions while arrows indicate viral protein granules. **B)** Enlarged cropped images of viral structures indicated by white boxes in (A). Representative single plane images are shown. Intensity profiles are drawn along the white line on the cropped image.

Using rRSVflag2-L, we then characterized L localization with respect to the ribonucleocapsid complex proteins, N, P, and M2-1, and the viral envelope F protein (Fig 1, S2 Fig) over the course of infection. A549 cells were infected with rRSVflag2-L and fixed and processed for immunofluorescence at different times post infection. To enable us to capture early events, we considered the time at which virus was added to the cells to be time 0. During early time points from 1 to 12 h post infection (hpi), L protein was observed to be present along with other viral proteins, including N, P and M2-1, in small viral cytoplasmic protein granules <0.8 μm in diameter (Fig 1, S2 Fig). In some cases, F protein was also present, most likely indicating viral particles infecting the cells (Fig 1, S2 Fig). Based on studies with human metapneumovirus (HMPV), another member of the pneumovirus family, granules lacking F are likely incoming nucleocapsids and sites of early RNA synthesis [36]. At 24 hpi, L protein was found in three main structures characteristic of late stage RSV infection: IBs, filaments, and small cytoplasmic granules that we have defined as assembly granules (Fig 1B; S2B Fig, S2D Fig). Assembly granules are cytoplasmic clusters of protein, which are distinct from IBs due to their smaller size and the presence of RSV F [12,13]. L protein was observed throughout the interior of IBs (Fig 1B; S2B Fig, S2D Fig). L protein was also present in viral filaments, though it was not homogenously distributed across the entire length, instead appearing to be localized to one end of the filament (Fig 1B; S2B Fig, S2D Fig). At later timepoints, L protein was also clearly present in assembly granules (Fig 1B; S2B Fig, S2D Fig)

**Fig 2:**
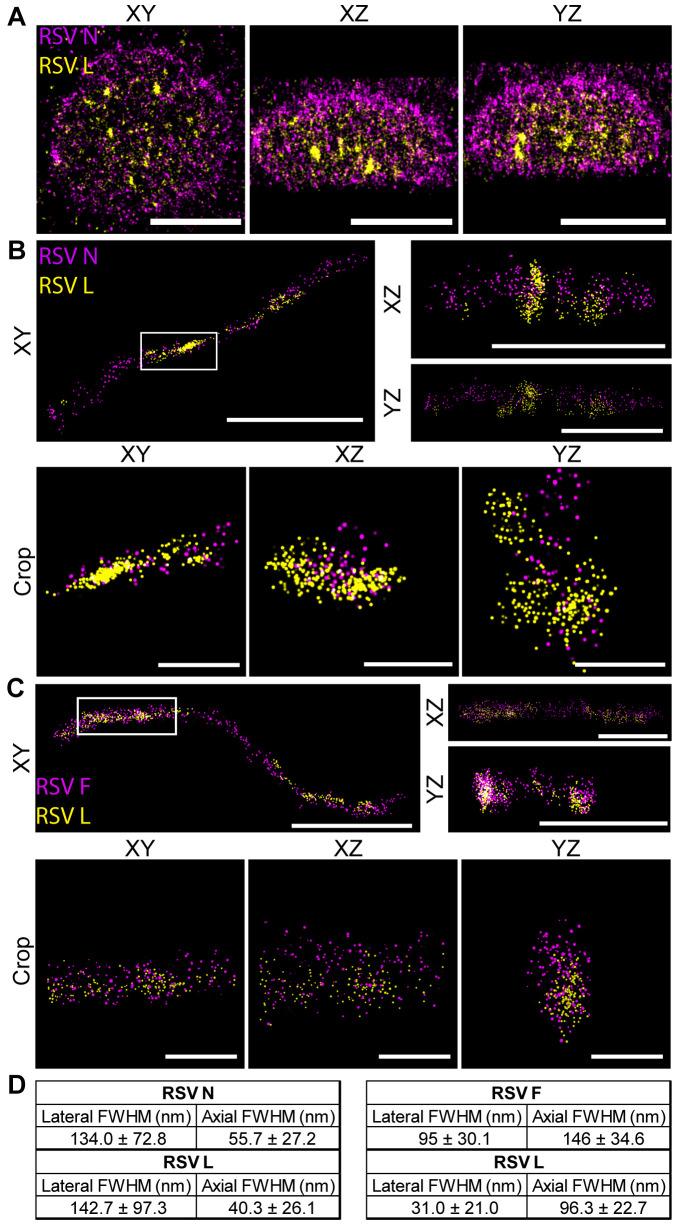
dSTORM images reveal RSV L localizes to the center of inclusion bodies and virions. **A)** A549 cells were infected with rRSVflag(2)L at a MOI of 3. Cells were fixed at 24 hpi and stained for RSV L (yellow) and RSV N (magenta). Representative dSTORM images of an inclusion body are shown, with a 2 μm scale bar. XY views, as well as XZ and YZ, are shown. **B)** Viral filaments on glass were stained for RSV L (yellow) and RSV N (magenta). XY views, as well as XZ and YZ, are shown, with a 1 μm scale bar. The white boxed region represents a 500 nm cropped region shown below in the XY, XZ, and YZ views with 250 nm scale bars. **C)** Viral filaments on glass were stained for RSV L (yellow) and RSV F (magenta). XY views, as well as XZ and YZ, are shown, with a 1 μm scale bar. The white boxed region represents cropped region shown below in the XY, XZ, and YZ views with 250 nm scale bars. D) Tables with full width at half maximum (FWHM) of the point count of the crops in (B) (left) and (C) (right). This measurement was repeated for a total of n = 3 filaments.

Next, we employed dSTORM to more closely examine L localization in IBs and filaments at 24 hpi by super-resolution microscopy. This approach allowed us to achieve ~20 nm lateral resolution. At 24 hpi, L was observed throughout the interior of IBs, although it was more highly concentrated in some areas (Fig 2A). Conversely, N was detected predominantly on the periphery of the IB, likely due at least in part to a lack of epitope accessibility, as previously observed [12]. In RSV filaments, L protein was observed in close proximity to N on the interior of the filament, with F surrounding it on the exterior (Fig 2B and 2C). The location of L in filaments was quantified by measuring the full width half maximums (FWHM) of each protein signal in the cross section of a viral filament as described previously [37]. The FWHMs for L were similar to those of N and smaller than F (Fig 2D), indicating that N and L were in the interior of filamentous virion, as expected. A large amount of L protein was clustered towards the end of the filament in relation to F protein, as observed previously with confocal microscopy (Figs 1B and 2C, S2B Fig, S2D Fig). However, the higher resolution provided by dSTORM imaging revealed that L protein is additionally clustered in other areas across the filament (Fig 2B and 2C).

### Ribonucleocapsids underwent structural rearrangement between 6 and 8 hpi

**Fig 3:**
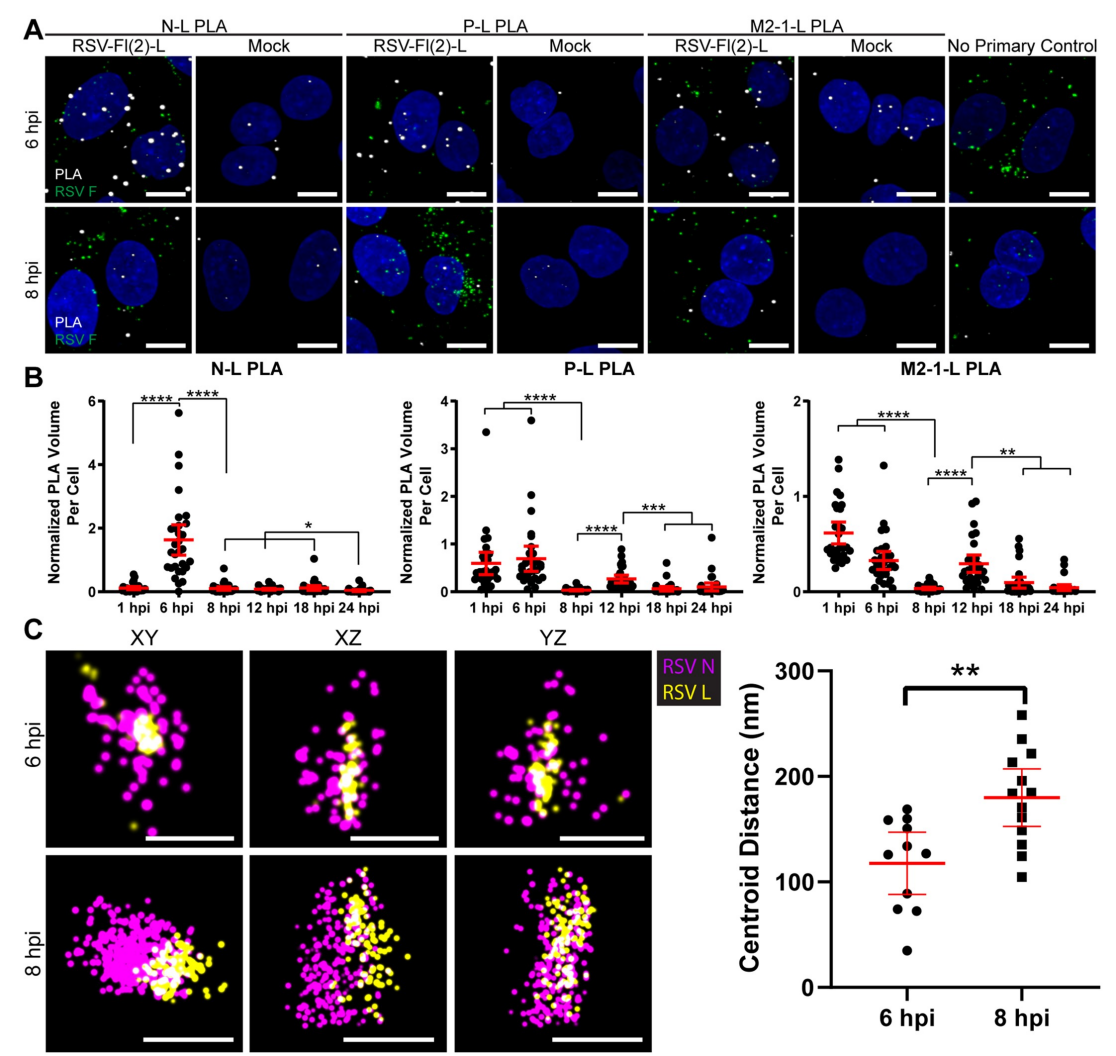
RSV proteins in the ribonucleocapsid complex rearrange between 6 and 8 hpi. **A)** A549 cells were infected or mock infected with rRSVflag(2)L at a MOI of 3. Cells were fixed at 1, 6, 8, 12, 18, and 24 hours post infection. PLA (white) was performed between N-L, P-L, and M2-1-L. Cells were stained for RSV F (green) to select for positively infected cells. Duplicates were performed, but representative extended focus images from one experiment are shown. Scale bar is 10 μm. Images for additional timepoints are reported in S3 Fig. **B)** Quantification of PLA in (A) is shown. PLA volume was normalized by volume of RSV F in the cell. Mean and 95% confidence intervals are shown in red. Kruskal-Wallis tests with a Dunn’s multiple comparison test were performed, where n = 30 cells and * p < 0.05, ** p < 0.01, *** p < 0.001, and **** p < 0.0001. **C)** A549 cells were infected with rRSVflag(2)L at a MOI of 3 and fixed at 6 or 8 hpi. Cells were stained for L (yellow) or N (magenta) and ribonucleocapsid complexes imaged with dSTORM. XY, XY, and YZ views are shown, with a 200 nm scale bar. XYZ positions of the centroid of the L and N protein clusters were measured, and the distance between L and N were quantified. Mean and 95% confidence intervals are shown in red. 5–10 infected cells were imaged for each timepoint across two experiments A Welch t-test was performed, where n = 10 and ** p < 0.01.

We utilized PLA to investigate the spatial relationship of L with the other ribonucleocapsid proteins at different stages of infection over a timecourse of RSV infection (Fig 3A and 3B, S3A and S3B Fig). In PLA, fixed and permeabilized cells are incubated with antibodies against the molecules of interest, which are in turn detected with secondary antibodies. If the secondary antibodies are in proximity (<40 nm) to each other, a PLA signal is generated upon ligation between the antibodies and the consequent rolling circle amplification. The number of PLA puncta can then be quantified to determine the relative frequency with which the two molecules of interest are in proximity to each other. As all antibodies bind in a non-specific manner to some degree, and PLA is a particularly sensitive technique, some background signal is always observed. Therefore, multiple controls were included for each timepoint and condition, to distinguish between interactions versus non-specific binding. A no primary antibody control was performed to ensure that the PLA probes, ligation reagents, and amplification reagents alone did not generate a significant number of PLA puncta. A mock infected control was included to account for nonspecific binding of the antibodies to other molecules in the cell. Because the background signal may change over time, due to the cell cycle, a mock control was included for each timepoint.

Comparison of the number of puncta in test samples versus controls revealed that there were a significant number of L: N and L: P interactions at 6 hpi (S3B Fig). However, surprisingly, these interactions had decreased to background levels at 8 hpi (S3B Fig). After 8 hpi, the number of interactions between L and the other proteins significantly increased until 24 hpi (S3B Fig). While we observed variation in the number of puncta in the mock controls at different times post infection, there was no significant difference between timepoints (S1 Table). Viral proteins accumulate in cells over time, which inherently results in an increased number of PLA puncta at late time points of infection. To account for this, we also normalized the number of PLA interactions to the viral protein content at each timepoint (Fig 3B). The ratio of the volume of PLA signal compared to the volume of viral protein present on a per cell basis was calculated, with the volume of viral protein being defined by the signals obtained by staining with either an F-specific or pan-RSV antibody. Both the F and pan-RSV antibody signals increased proportionally to each other over time during infection, so both approaches could be used interchangeably (S3C Fig). According to this analysis, the frequency of L: N and L: P interactions increased between 1 and 6 hpi, and then decreased between 6 and 8 hpi, and remained low for the remainder of the timecourse (Fig 3B).

As another approach to study the relationship between L and N proteins, we analyzed cytoplasmic granules using dSTORM (Fig 3C). Consistent with the findings using PLA, we observed a significant increase in distance between the clusters of L and N protein between 6 and 8 hpi. Interestingly, the proteins did not appear to dissociate completely but rather rearranged their relative distributions within the granule. To assess whether this rearrangement was related to disassembly of the incoming virion, we quantified the proximity between F and L proteins. F and L were in close proximity at 1 hpi, but not at 6 or 8 hpi, as measured both by the number of puncta and normalized PLA volume (S4A and S4B Fig). Together these results show that there is a structural rearrangement of the nucleocapsid proteins within granules between 6 and 8 hpi that is unrelated to virion disassembly.

### Ribonucleocapsid rearrangement was concomitant with the onset of RNA replication and secondary transcription

We hypothesized that rearrangement of the ribonucleocapsid complex could correspond to the onset of antigenome synthesis following primary transcription. To test this hypothesis, we first analyzed the amounts of negative strand (RSV genome) and positive strand (RSV mRNA/antigenome) synthesized over time by Northern blot and RT-qPCR (S5 Fig). Unfortunately, we were not able to reliably detect antigenome RNA, most likely because only a small quantity is produced during the infection [21,38]. Both RSV genome and mRNA slowly increased from 0 to 8 hpi, followed by a more dramatic increase in synthesis (S1 Fig, S5 Fig). This is consistent with the first strands of newly synthesized antigenome and genome RNAs being produced by approximately 8 hpi, followed by secondary transcription and further rounds of RNA replication.

**Fig 4:**
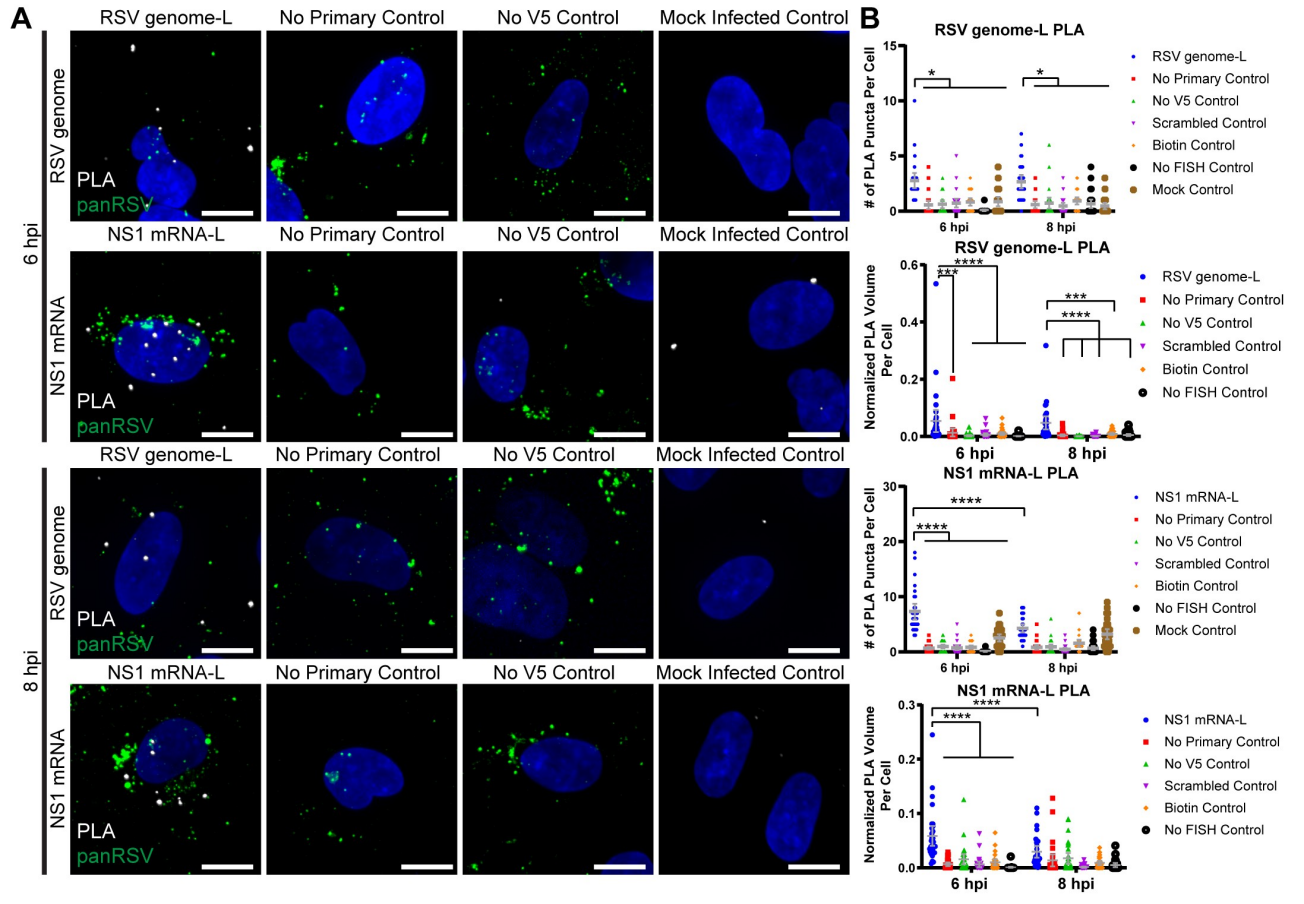
The positions of RSV L, RSV genome, and RSV mRNA in the ribonucleocapsid complex rearrange over time. **A)** A549 cells were infected or mock infected with rRSVflag(2)L at a MOI of 3. Cells were fixed at 6, 8, 12, 18, and 24 hours post infection. FISH with multiply-labeled tetravalent RNA imaging probes (MTRIPs) was performed for RSV genome, RSV NS1 mRNA, without an epitope tag, a scrambled control, or without targeting oligos. PLA (white) was performed between RSV genome/ NS1 mRNA and L protein. Cells were stained for panRSV (green) to select for positively infected cells. Duplicates were performed, but representative extended focus images from one experiment are shown. Scale bar is 10 μm. **B)** Quantification of PLA in (A) is shown. PLA volume was normalized by volume of panRSV in the cell. Mean and 95% confidence intervals are shown in grey. Statistics for the number of puncta per cell were two-way ANOVAs with Holm-Sidak’s multiple comparisons tests, where * p < 0.05, and **** p < 0.0001. Statistics for the fractions of interactions per cell were two-way ANOVAs with Tukey’s multiple comparison tests, where *** p < 0.001, and **** p < 0.0001. n = 30 cells per experiment. Additional results are reported in S6 Fig and S7 Fig.

We reasoned that the onset of antigenome and genome synthesis would correspond to a decrease in L interactions with mRNA, as a smaller proportion of polymerase molecules would be engaged in transcription. To test this hypothesis, we quantified the number of interactions and the normalized volume of interactions between L protein and either RSV genome or NS1 mRNA over time using PLA (Fig 4A and 4B, S6A–S6C Fig, S7A and S7B Fig) [33]. Additional controls specific for this fluorescence *in situ* hybridization (FISH)-based approach included controls that accounted for the background signal generated by nonspecific binding of the FISH probes. Analysis of total L protein and genome RNA PLA interactions showed that they were significantly above background at all times analyzed, with an increase between 8 and 12 hpi (Fig 4A and 4B, S6A–S6C Fig). The number of interactions in the mock controls were not significantly different from each other over time, with the exception of the 12 hpi timepoint (S1 Table). However, at 12 hpi, the experimental condition was significantly different than all of its controls. Normalization of total L: genome interactions to viral protein showed significant levels of interactions at 6, 8 and 12 hpi, with a reduction to background levels at 18 and 24 hpi due to the high level of viral protein expression at these times (Fig 4A and 4B, S6A–S6C Fig). In contrast, L: NS1 mRNA interactions showed a different pattern. The levels of total L: NS1 mRNA PLA interactions were high at 6 hpi, but decreased to background levels at 8 hpi, then increased to high levels at 12, 18 and 24 hpi, consistent with the high levels of mRNA synthesis at these times (Fig 4A and 4B, S7A and S7B Fig). When these PLA interactions were normalized to total protein, the level of L: NS1 mRNA PLA interactions were significantly above background at 6 hpi but then diminished to background levels at later timepoints (Fig 4A and 4B, S7A and S7B Fig). Interactions between L and polyadenylated mRNA showed a similar trend, indicating that the analysis reflected interactions between L protein and NS1 mRNA, rather than antigenome (S7C–S7E Fig). These data clearly show a proportional decrease in L: mRNA interactions between 6 and 8 hpi, coinciding with the structural rearrangement of the ribonucleocapsid and the onset of RNA replication.

### M2-2 inhibited RSV gene expression when exogenously expressed at an early stage of infection, but not if expressed at a later stage

**Fig 5:**
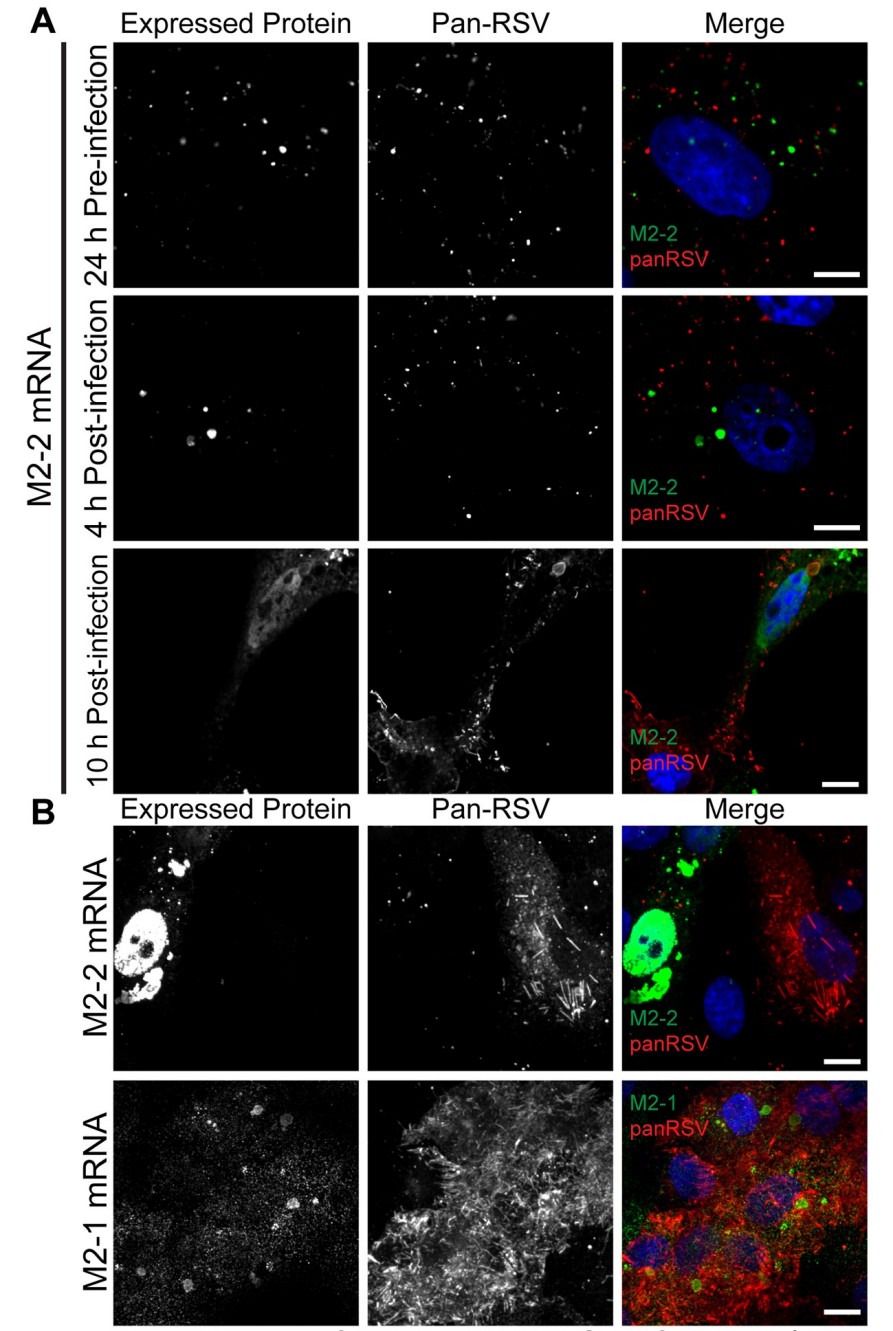
M2-2 overexpression early in infection inhibits RSV infection. **A)** Vero cells were transfected with 200 ng of M2-2-Myc mRNA 24 h pre-infection, 4 hpi, or 10 hpi. Cells were infected with RSV at a MOI of 3 and fixed 24 hpi. Cells were stained for M2-2 via Myc (green), and panRSV (red). Representative single plane images are shown, with a scale bar of 10 μm. Control images are presented in S8 Fig. **B)** Vero cells were infected with RSV at a MOI of 3 and transfected with 200 ng of M2-2-Myc or M2-1-HA mRNA 3 hpi. Cells were fixed at 24 hpi and stained for M2-2 via Myc (green) or M2-1 via HA (green), and panRSV (red). Representative extended focus images are shown, with a scale bar of 10 μm.

As noted in the introduction, M2-2 protein is thought to play a regulatory role, suppressing transcription and enhancing genome replication [25,39,40]. We hypothesized that this M2-2 function might be linked to the structural reorganization of the ribonucleocapsid described above. To test this hypothesis, we generated *in vitro* transcribed mRNA encoding Myc-tagged M2-2. *In vitro* transcribed mRNA is expressed as protein by 2 hours post transfection [41] and so transfection of mRNA is a rapid and efficient way to produce exogenous proteins, allowing us to perturb the viral infection cycle at defined timepoints [41]. A Myc tag was included on M2-2 to allow for visualization of exogenous M2-2, as no M2-2 specific antibody is currently available. However, the Myc tag specific antibody was found to have significant background signal in human A549 cells. Consequently, experiments to elucidate the role of M2-2 overexpression were performed in Vero cells. To verify that the kinetics of RSV infection were not different between the cell types at the experimental timepoints, dSTORM analysis of ribonucleocapsid complex rearrangements at 6 and 8 hpi in Vero cells was performed and compared to the analysis in A549 cells (Fig 3C, S8A Fig). No significant difference was observed between the cell types. Next, cells were transfected with either M2-2 mRNA or a control mRNA encoding GFP, either 24 h prior to infection, or at 4- or 10-hours post infection. 24 hours after infection, cells were prepared for immunofluorescence analysis using pan-RSV antibodies to detect infected cells and Myc antibody to detect exogenously expressed M2-2 (Fig 5A, S8B Fig). Exogenous M2-2 was observed to be have both nuclear and cytoplasmic distribution (Fig 5A). It was also found to form large granules in both infected (Fig 5A) and mock infected cells (S8B Fig). It is possible that this localization and clusters are a result of overexpression of M2-2, as it is typically only expressed at very low amounts during infection [42]. However, when transfected late in infection at 10 hpi, exogenous M2-2 was localized in IBs, as demarcated by the panRSV antibody (Fig 5A) suggesting that it associates with ribonucleocapsid proteins. Assessment of the viral infection using the pan-RSV antibody showed that when M2-2 mRNA was transfected prior to infection or at 4 hpi, viral protein expression was minimal and proteins were only detected in small puncta, indicating that cells did not progress to late stage infection. This inhibition did not occur in cells transfected with GFP (S8B Fig). In contrast, when M2-2 mRNA was transfected at 10 hpi, the infection was able to progress to a late stage, with abundant expression of viral proteins (Fig 5A). We further confirmed that the inhibition of infection was specific to M2-2 mRNA by overexpressing HA-tagged M2-1 protein via transfection of *in vitro* transcribed mRNA (Fig 5B). Cells transfected with M2-1 mRNA did progress to late stage infection, unlike those overexpressing M2-2. We further confirmed the inhibitory effects of M2-2 mRNA transfection early in infection by both plaque assays and RT-qPCR for F mRNA (S8C and S8D Fig). Cells transfected with M2-2 generated significantly fewer plaques and F mRNA than those transfected with either GFP or M2-1 mRNA.

### Exogenously expressed M2-2 protein caused premature ribonucleocapsid rearrangement

**Fig 6:**
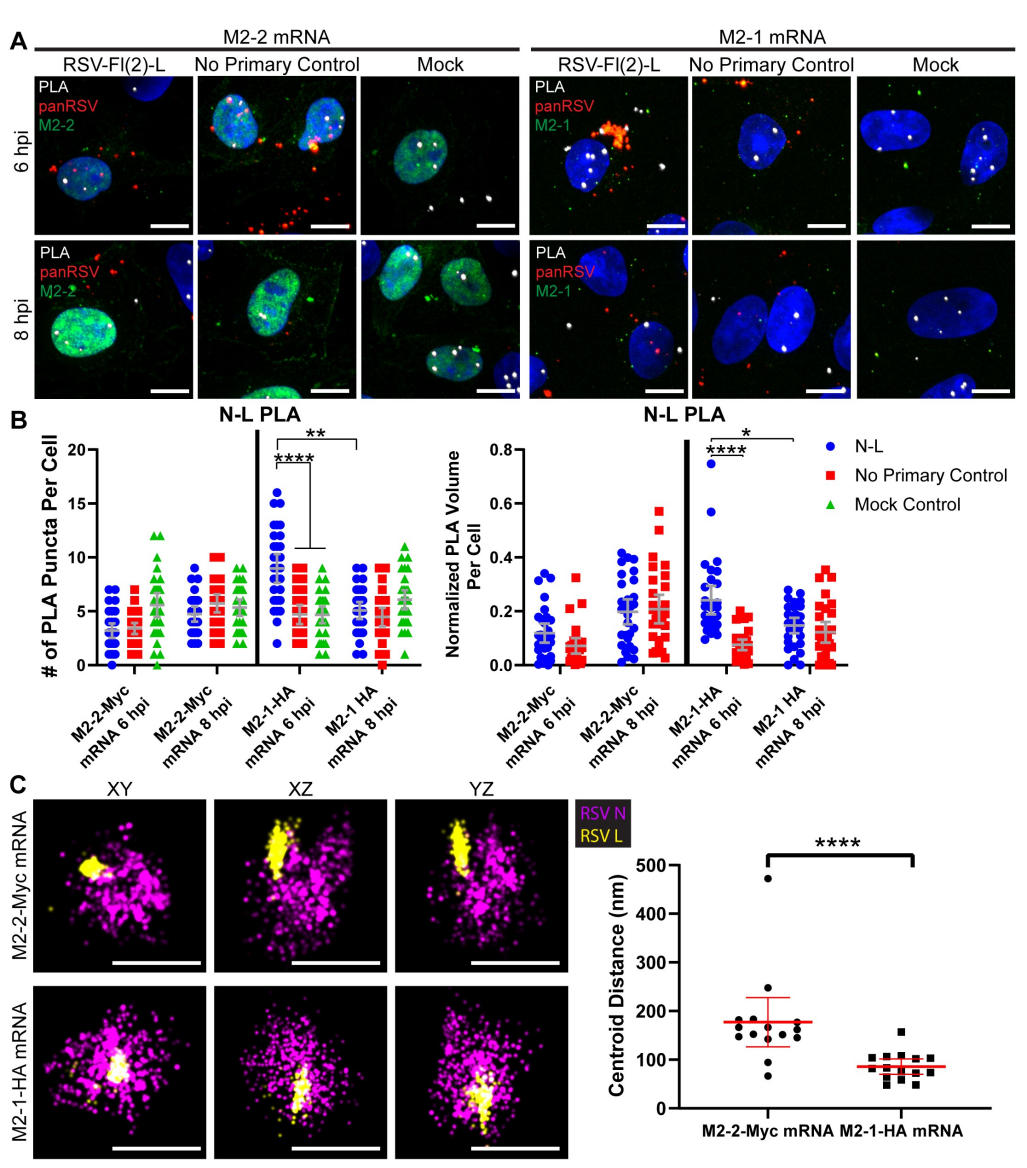
Overexpression of M2-2 forces ribonucleocapsid complex rearrangement. **A)** Vero cells were infected or mock infected with rRSVflag(2)L at a MOI of 3. Cells were transfected with 200 ng of M2-2-Myc or M2-1-HA mRNA at 3 hpi. Cells were fixed at 6 or 8 hours post infection. PLA (white) was performed between N and L. Cells were stained for M2-2 via Myc (green) or M2-1 via HA (green), and panRSV (red). Duplicates were performed, but representative extended focus images from one experiment are shown, with a scale bar of 10 μm. **B)** Quantification for (A) is shown. Two-way ANOVAs with Tukey’s multiple comparisons tests were performed, where n = 30 cells and * p < 0.05, ** p< 0.01 and **** p < 0.0001. Mean and 95% confidence intervals are shown in grey. PLA volume (right) is normalized by volume of panRSV per cell. **C)** Vero cells were infected with rRSVflag(2)L at a MOI of 3. Cells were transfected with 500 ng of M2-2-Myc or M2-1-HA mRNA at 3 hpi. Cells were fixed at 6 hours post infection and stained for N (magenta) or L (yellow). Cells were also stained for M2-2 via Myc or M2-1 via HA as a reference color to positively select transfected cells. Ribonucleocapsid complexes in transfected cells were imaged with dSTORM. XY, XZ, and YZ views are shown, with a 300 nm scale bar. XYZ positions of the centroid of the L and N protein clusters were measured, and the distance between L and N were quantified. Mean and 95% confidence intervals are shown in red. 5–10 infected cells were imaged for each condition across two infections. A Mann-Whitney t-test was performed, where n = 15 granules and **** p < 0.0001.

**Fig 7:**
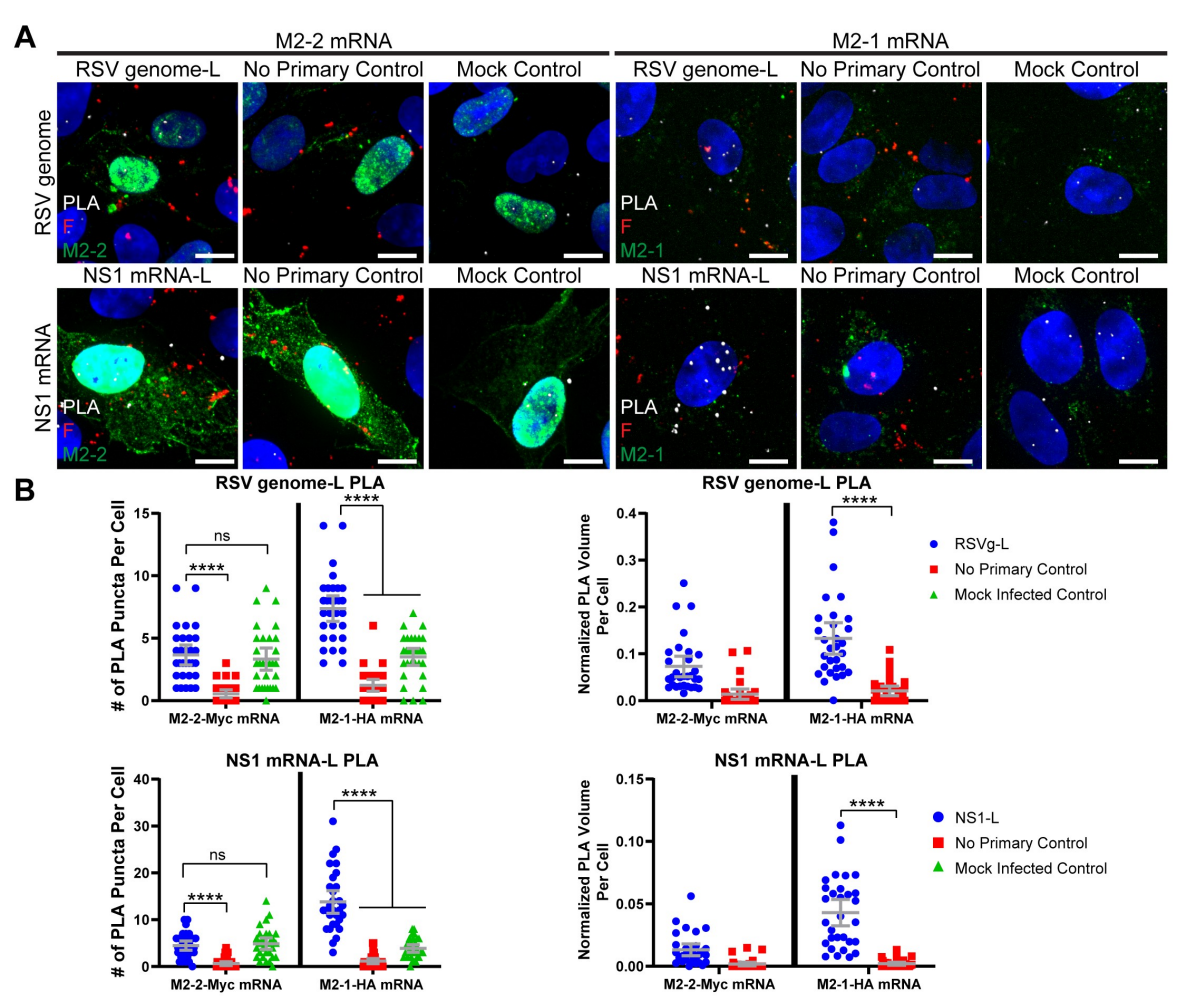
Overexpressing M2-2 reduces interactions of L with RSV genome and mRNA. **A)** Vero cells were infected or mock infected with rRSVflag(2)L at a MOI of 3. Cells were transfected with 200 ng of M2-2-Myc or M2-1-HA mRNA 3 hpi. Cells were fixed at 6 h post infection. FISH with PMTRIPs was performed for RSV genome or RSV NS1 mRNA. PLA (white) was performed between RSV genome/ NS1 mRNA and L. Cells were stained for M2-2 via Myc (green) or M2-1 via HA (green), and F (red). Duplicates were performed, but representative extended focus images from one experiment are shown. Scale bar is 10 μm. Representative images for additional controls and timepoints are described in S9 Fig. **B)** Quantification of PLA in (A) at 6 hpi is shown. Additional quantifications are described in S9 Fig. Mean and 95% confidence intervals are shown in grey. Two-way ANOVAs with Tukey’s multiple comparison tests were performed, where n = 30 cells and * p < 0.05, *** p < 0.001, and **** p < 0.0001. PLA volume (right) is normalized by volume of RSV F per cell.

We then examined the effect of M2-2 on ribonucleocapsid interactions. We transfected M2-2 or M2-1 mRNA at 3 hpi, to ensure protein expression prior to the observed endogenous viral protein rearrangement at 6–8 hpi. We quantified total and normalized PLA interactions between L and N proteins at 6 and 8 hpi (Fig 6A and 6B). When cells were transfected with M2-2 mRNA, no significant PLA interactions between L and N were detected at 6 hpi, whereas cells transfected with M2-1 retained a significant number of L: N protein interactions at 6 hpi. Using immunofluorescence microscopy, we confirmed that the decrease in interactions in cells transfected with M2-2 RNA was not merely caused by M2-2 overexpression affecting epitope access to L (S8E and S8F Fig). We next confirmed that the rearrangement caused by M2-2 overexpression at 6 hpi could also be observed via dSTORM imaging (Fig 6C). N and L in ribonucleocapsid complexes were significantly further apart after M2-2 overexpression than with M2-1 overexpression. However, the proteins did not appear to completely disassociate. The effect of M2-2 on L protein interactions with RSV RNAs was quantified using PLA (Fig 7A and 7B, S9A–S9C Fig). Whereas L: genome RNA interactions were detectable in cells transfected with M2-1 RNA and analyzed at 6 hpi, there was no significant interaction detected in cells transfected with M2-2 RNA at 6 hpi (Fig 7A and 7B). This was also the case at 8 hpi (S9B and S9C Fig). Similarly, there were no significant interactions between L and NS1 mRNA at 6 hpi in cells transfected with M2-2 mRNA, unlike the situation with M2-1 mRNA (Fig 7A and 7B, S9A–S9C Fig).

## Discussion

Here, we present the detailed characterization of the spatio-temporal relationships between the polymerase and other ribonucleocapsid components at different times over the course of RSV infection. We show that RSV ribonucleocapsids undergo a structural reorganization at 6–8 hpi, concomitant with the onset of genome replication. Further, we show that ribonucleocapsid reorganization can be elicited by expression of the M2-2 protein, and that premature expression of M2-2 can impair viral gene expression.

By introducing an epitope tag into the RSV L protein expressed by recombinant RSV, we were able to track the polymerase over the course of infection. During the early stages of RSV infection, at 1 and 6 hpi, L protein was primarily localized within diffraction-limited cytosolic protein granules (< 0.8 μm in diameter), colocalizing with other RSV proteins, including N, P, M2-1 and in some cases F (Fig 1, S2 Fig). These early viral granules that form before inclusion bodies have previously been observed to contain N and RSV genome RNA and they form spontaneously if N and P are coexpressed via plasmid transfection [12,43,44]. Using PLA, we found that the number of F and L protein containing granules significantly decreased between 1 and 6 hpi (S4 Fig). Thus, it is likely that granules that contained F protein were virus particles entering the cell, either at the plasma membrane, or by macropinocytosis, whereas those lacking F represent delivered nucleocapsids and sites of early RSV transcription. By 12 hpi, the L protein was localized within larger inclusion body structures that also contained N, P and M2-1 proteins (Fig 1, S2 Fig). The inclusion bodies increased in size, such that by 24 hpi they were ~1–5 μm in diameter (Fig 1, S2 Fig). Inclusion bodies are a common feature of non-segmented negative strand RNA virus infection and have been shown or implicated to be sites of RNA synthesis for a number of viruses [11,36,45–50]. Inclusion bodies can undergo fusion and fission, and studies with rabies virus and VSV have shown that they have properties of liquid organelles [36,45,51,52]. Consistent with this, RSV inclusion bodies are bounded by N and P proteins, similarly to those of HMPV (Figs 1 and 2, and S2 Fig) [11,12,36,44,53]. A previous study of RSV inclusion bodies showed that L protein was also on the periphery, rather than within the inclusion body [10]. However, the confocal microscopy and dSTORM analysis presented here (Figs 1B and 2A, S2B Fig, S2D Fig), as well as work by Rincheval *et al*., reveals that L is localized throughout inclusion bodies, in a non-uniform distribution [11]. While Rincheval *et al*. observed L protein forming large rings surrounding IBAGs, we instead observed clusters of L protein through the inclusion body (Figs 1B and 2A, S2B Fig, S2D Fig). The discrepancy between these studies is most likely due to the different methodologies used to express L protein. In the present study, we used infectious recombinant virus, such that all virus proteins were present and L protein would have been expressed at appropriate levels compared to other viral proteins. In contrast, Rincheval and coworkers used a minireplicon system, which involves overexpression of a subset of RSV proteins [11]. Given that L is only expressed at very low levels in RSV infection, it is possible that over-expression led to artefactual accumulation. Finally, at 24 hpi, we also observed L in assembly granules and viral filaments (Figs 1 and 2, S2 Fig). Assembly granules contain F protein, are <1 μm in diameter, and have been observed in late stage infection in both RSV and HMPV infected cells [13,15,36]. We have previously observed virus filaments both extending from and retracting into assembly granules [13]. Viral filaments then protrude from the surface of infected cells, having a length of up to ~12 μm [15,16]. L protein was observed throughout the center of viral filaments, in close proximity to N, although with increased clustering towards the end of the filament (Fig 2B). This is similar to what has previously been observed with VSV and could occur if the polymerase continues to elongate RNA and move towards the 5´ end of the genome as the viral filament is being formed [54].

The early viral granules detected up to 12 hpi are roughly the size of a point spread function in confocal microscopy, meaning they are approximately equal to, or smaller than, the resolution limit of the utilized microscope. However, we were able to distinguish their components as separate structures and study interactions with other RSV proteins using dSTORM and PLA. Surprisingly, we found that ribonucleocapsid components undergo significant reorganization between 6 and 8 hpi. By using PLA to characterize the relationship of L with N, P and M2-1 at different times post infection, we observed a significant decrease in the total and normalized numbers of L interactions with the other ribonucleocapsid complex proteins, particularly N, between 6 and 8 hpi, (Fig 3A and 3B, S3A and S3B Fig). These findings were substantiated by dSTORM analysis, which showed that the centroids of L and N protein clusters within a granule were significantly farther apart at 8 hpi than at 6 hpi (Fig 3C). While it is not possible to guarantee we were analyzing exactly the same type granules at the different timepoints, as dSTORM was performed post-fixation, the structures measured are roughly the same size scale between both timepoints and both contain the N and L proteins. Furthermore, they are the primary structures observed at 6 hpi and distinct from the only other structures occasionally observed at 8 hpi, early inclusion bodies, which are characterized by a “hollow” N protein staining pattern. Therefore, we believe a clear rearrangement in granule structure is being observed. This indicates that the available epitopes on L and N proteins move apart between 6–8 hpi. Because there are multiple secondary antibodies and fluorophores bound to each protein epitope, and because the fluorophores could be positioned some distance away from the protein they represent due to the intervening antibodies, it is not possible to identify how many individual L and N proteins are being detected in the dSTORM analysis shown in Fig 3C, or to accurately measure distances between these proteins. Nonetheless, for reference, the diameter of the L protein is approximately 15 nm, while the width and length of the RSV nucleocapsid is 10–16 nm and ~1000 nm, respectively [55,56]. Therefore, the ~50–75 nm average centroid distance change detected by dSTORM, and >40 nm proximity changes detected by PLA, could relate to individual encapsidated genomes adopting a different conformation with respect to the associated L protein. Importantly, the protein clusters did not appear to completely disassociate, but rather to rearrange their relative positions in the complex, suggesting a structural reorganization of the ribonucleocapsid.

Reorganization of the ribonucleocapsids occurred after dissociation of F protein (S4 Fig), indicating that the ribonucleocapsid reorganization that occurred between 6 and 8 hpi was not linked to fusion and entry of the incoming virion. Instead, we hypothesized that the ribonucleocapsid rearrangement that was detected corresponds to the switch in which ribonucleocapsids that were engaged exclusively in primary transcription begin to engage in RNA replication. Consistent with this hypothesis, we detected that levels of genome RNA began to increase at 8 hpi, and we detected a significant decrease in L-mRNA interactions between 6 and 8 hpi, with no significant change in L-genome interactions (Fig 4, S5 Fig, S6 Fig, S7 Fig). Assuming that the positive sense antigenome RNA levels were below the level of detection and did not factor into the PLA data, this finding is consistent with the hypothesis that a significant proportion of ribonucleocapsid complexes were diverted from transcription to replication between 6 to 8 hpi. The total number of L-mRNA interactions then increased again from 8 to 12 hpi and remained high until the end of the timecourse (Fig 4, S7 Fig). This finding is consistent with a scenario in which a proportion of newly synthesized genomes engaged in transcription accounting for the observed increase in mRNA levels after 8 hpi (S1 Fig and S5 Fig). However, the normalized level of L: mRNA interaction never reached the levels detected at early times of infection, indicating a significant shift in ribonucleocapsid function between 6 and 8 hpi. The discrepancy between the total number of interactions and the normalized number of interactions at later timepoints is a result of the high levels of viral proteins generated by late stage infection. This is likely an indication that while transcription of mRNAs and replication via generation of antigenome are both still occurring at late timepoints, the cell is dominated by assembly of virions from already produced proteins and genome. Taken together, the results presented in Fig 3, Fig 4, S1 Fig, S3 Fig, S5 Fig, S6 Fig and S7 Fig indicate that the onset of RNA replication is accompanied by a dynamic rearrangement of ribonucleocapsid proteins. As noted in the Introduction, it was previously known that the ribonucleocapsids of non-segmented negative strand RNA viruses can adopt different conformations [26–31]. However, to our knowledge, the present study presents the first evidence that ribonucleocapsid conformational changes are associated with the onset of replication.

M2-2 is a non-essential accessory protein. Ablation of the M2-2 open reading frame from the RSV genome results in increased levels of mRNAs and decreased levels of genome RNA, indicating that M2-2 plays a role in inhibiting transcription while promoting RNA replication [25,39,40]. Studies using the RSV minigenome system showed that over-expression of M2-2 inhibited both transcription and replication [6] and previous studies have shown that over-expression of M2-2 protein in the context of RSV infection is highly detrimental to the virus [39,40]. This suggests that at high concentrations, M2-2 inhibition is less specific and that all RSV RNA synthesis is inhibited. Here, we observed that M2-2 transfection prior to infection, or at 4 hpi, suppressed RSV infection, preventing formation of large inclusion bodies and viral filaments (Fig 5A). In contrast, transfection of M2-2 RNA at 10 hpi had no detectable effect (Fig 5A). These results confirm the inhibitory effect of M2-2, but importantly, show that M2-2 is not simply a general inhibitor of RSV RNA synthesis, but rather exerts its effect within a specific window, between 4 and 10 hpi, likely before the window of 6–8 hpi when rearrangement was observed. Analysis using PLA and dSTORM imaging showed that early overexpression of M2-2 reduced the levels of L-N protein interactions and induced a rearrangement in ribonucleoprotein complexes (Fig 6). Additionally, M2-2 transfection resulted in decreased interactions between L and RSV NS1 mRNA (Fig 7, S9 Fig). It should be noted that M2-2 expression also resulted in decreased interactions of L with genome RNA, which does not replicate the situation observed at 8 hpi in non-transfected cells (Fig 7, S9 Fig), and might be a consequence of the high-level expression of M2-2. Nonetheless, these findings are consistent with the hypothesis that M2-2 affects RNA synthesis by inducing rearrangement of the ribonucleocapsid complex, and that this rearrangement must occur at a certain time in infection. If it happens prematurely, the infection cannot proceed, most likely because there is insufficient N and P proteins available for RNA replication to occur.

Based on these results we propose the following model for the events that occur during RSV infection. When genome-containing nucleocapsids are delivered into the cell, their default mode is mRNA transcription, resulting in accumulation of all viral proteins, including N and P which are required for antigenome synthesis, and M2-2. Accumulation of M2-2 elicits a ribonucleocapsid rearrangement which causes a switch from primary transcription to antigenome synthesis, facilitated by newly-synthesized N and P proteins, and subsequent accumulation of genome ribonucleocapsids. After this rearrangement, M2-2 ceases to have a detectable effect and newly synthesized genomes are capable of transcription. It is possible that the proliferation of genome ribonucleocapsids leads to M2-2 being outcompeted, allowing secondary transcription to occur from the majority of genome templates, with a small number of genome ribonucleocapsids remaining associated with M2-2 and continuing to be committed to antigenome synthesis.

In summary, this study provides new insight into the dynamics of RSV ribonucleocapsids and suggests that ribonucleocapsid conformation relates to its transcription and replication activities. Further studies are required to determine if genome ribonucleocapsids become exclusively committed to transcription or replication, as our model proposes, and to determine exactly how M2-2 functions.

## Methods

### Cell lines

A549 (ATCC, human, male), Vero (ATCC, primate, female), or HEp-2 cells (ATCC, human, female) were maintained in DMEM (Lonza) supplemented with 10% fetal bovine serum (FBS) (Hyclone) and 100 U/ml penicillin and 100 mg/ml streptomycin (pen-strep) (Life Technologies). All cells were cultured at 37°C and 5% CO_2_. Cells were authenticated by ATCC and checked for mycoplasma contamination in our laboratory.

### Recombinant virus generation

To generate recombinant viruses, a plasmid that encodes the RSV antigenome was used [57,58]. To facilitate the insertion of tags into the L protein, a KpnI restriction site was introduced into the L gene at a position after amino acid 1749 in L using a q5 Site-directed mutagenesis kit (NEB). Two FLAG tags were cloned into this region using KpnI-HF (NEB). Full-length clones of RSV encoding a flag(2)-L were subjected to restriction digests and sequencing to confirm their identity. The virus was rescued as previously described and sequenced to confirm the integrity of the tag [57,59].

### Viruses

HEp-2 cells were used to propagate human RSV A2 (ATCC, Cat# VR-1544) and rRSVflag(2)L in culture. To propagate, cells at 80% confluence were washed with DPBS without Ca^2+^ and Mg^+^ (Lonza) and virus was added at a multiplicity of infection (MOI) of 0.1 diluted in complete medium for 1 h at 37°C. Complete medium to culture volume was then added to the culture for prolonged inoculation at 37°C. When high cytopathic effects were observed (~4–7 days), cells were scraped to release cell associated virus. Medium was then collected, aliquoted, and stored at either -80°C or in liquid nitrogen. Virus titers were measured via plaque assay.

### Antibodies

For plaques, immunostaining, and PLA, primary antibodies used were mouse anti-RSV N (Novus Biologics, Cat. No. NB100-64752 1:500 dilution), human anti-RSV F (Palivizumab, Medimmune, 1 μg/mL), mouse anti-RSV P (Abcam, Cat. No. ab94965, 1:1000 dilution), mouse anti-RSV M2-1 (Abcam, Cat, No. ab94805, 1:1000 dilution), goat anti-panRSV (Abcam, Cat. No. ab20745, 1:1000 dilution), rabbit anti-FLAG (Cell Signaling Technology, Cat. No. 14793S, 1:1000 dilution), mouse anti-V5 (Sigma Aldrich, Cat. No. V8012, 1:500 dilution), mouse anti-Myc (Cell Signaling Technology, Cat. No. 2276S, 1:1000 dilution), mouse anti-Myc-AF488 (Cell Signaling Technology, Cat. No. 2279S, 1:250 dilution), rabbit anti-HA (Santa Cruz, Cat. No. sc-805, 1:1000 dilution), and mouse anti-HA-AF488 (Novus Biologics, Cat. No. NBP2-50416AF488, 1:100 dilution). All respective secondary antibodies for immunostaining (Jackson Immunoresearch, Thermo Fisher) were used at 4 μg/ml. Secondary antibodies for PLA (Sigma Aldrich) were used according to manufacturer’s instructions.

### Virus multiple-step growth analysis

A549 cells were grown in F-12K media (ATCC) containing 10% FBS and were seeded in 6-well plates and infected with rRSV or rRSVflag(2)L in triplicate at an MOI of 0.01. After 1 hr absorption, media was replaced with 1 mL of serum containing media. At indicated timepoints, media overlay was snap-frozen and stored at -80°C and subsequently replaced every 24 hrs for 7 days. Virus titer was determined by plaque assay.

### Plaque assays

For plaque assays corresponding to titers presented in S1C Fig, plaque assays were carried out as described in Hanley *et al*. (2010) with the exception that A549 cells were used with overlay medium of F-12K media supplemented with 10% FBS, pen-strep, and 0.8% methylcellulose [58].To determine virus titer and for the plaque assays presented in S8C Fig, HEp-2 cells were plated in 24 well plates. When cells were confluent, virus was added as an inoculum for 1 h at 37°C. Overlay medium of DMEM, FBS, pen-strep, and avicel (FMC Biopolymer) was then added on top of inoculum, and cells were incubated for 6 days at 37°C before fixation with 4% paraformaldehyde (Electron Microscopy Sciences) for 10 minutes at room temperature. Cells were then blocked for 30 minutes at 37°C with 5% BSA (Sigma Aldrich). Cells were stained with an antibody specific for RSV F protein for 30 minutes at 37°C, followed by an anti-human horse radish peroxidase secondary antibody (Jackson Immunoresearch, 1:250 dilution) for 30 minutes at 37°C. Finally, cells were incubated with TrueBlue peroxidase substrate (SeraCare) for 10 minutes at RT. Plaques were then counted to determine titer. Biological replicates were performed, where n = 4.

### RSV infections

Medium was removed from confluent cells and cells were washed with DPBS without Ca^2+^ and Mg^+^. Virus was added at a MOI of 3, diluted in complete medium for 1 h at 37°C. Additional complete medium was then added on top of inoculum to maintain cells at 37°C until use. Time of virus addition was considered to be 0 hpi in all experiments.

### Western blot analysis of epitope-tagged L protein

A549 cells were infected with rRSVflag(2)L at an MOI = 3. At 20 hpi, cells were lysed in RIPA lysis buffer and genomic DNA was digested with TURBO DNaseI (Invitrogen, AM2238). 5% of the sample was run on a tris-glycine gel and was transferred to a nitrocellulose membrane using XCell II blot module (Invitrogen) for 2 hrs at 30V in a transfer buffer containing 10% methanol. Blot was analyzed with primary antibody ANTI-FLAG(R) M2 (Sigma-Aldrich, F3165) at a dilution of 1:500 and secondary antibody IRDye 800CW anti-Mouse IgG (Licor, 26–32212) at a dilution of 1:20,000.

### Northern blot analysis of RSV RNAs

A549 cells were grown in D-MEM containing 10% FBS and antibiotics and were seeded in 6-well plates. Cells were infected with either rRSV or rRSVflag(2) at an MOI of 3. At indicated time points, total RNA was harvested either by TRIzol extraction or RNeasy extraction with on-column DNase treatment. RNA was purified following manufacturer’s instructions. TRIzol extracted RNA was subjected to a second round of RNA purification by phenol:chloroform extraction. 5 μg of RNA from each sample was subjected to electrophoresis in 1.5% agarose-formaldehyde gels in MOPS buffer and subjected to Northern blot analysis with probes to detect genome RNA (5´-TTTATGCAAGTTTGTTGTACGCATTTTT-3´) or N mRNA (5´- TGACTTTGCTAAGAGCCATGGTTGT-3´) as previously described [19]. RNA was detected by autoradiography.

### Immunostaining for confocal microscopy

At the indicated timepoints, specified cells in a 96 well glass bottom plate (Cell-Vis) were fixed with 4% paraformaldehyde for 10 minutes at room temperature. Cells were then permeabilized with 0.2% Triton X-100 (Sigma Aldrich) for 5 minutes at room temperature. Next, cells were blocked with 5% BSA for 30 minutes at 37°C, before being incubated with the indicated primary antibody overnight at 4°C. Cells were then washed with PBS and incubated with secondary antibody for 30 minutes at 37°C. Cell nuclei were stained with 4’,6-diamidino-2-phenylindole (DAPI) (Life Technologies), and Prolong Gold (Life Technologies) was added on top of cells before imaging.

### Immunostaining for dSTORM

At the indicated timepoints, specified cells in an 8 chambered glass slide (Ibidi) were fixed with 4% paraformaldehyde for 10 minutes at room temperature. Cells were then permeabilized with 0.2% Triton X-100 (Sigma Aldrich) for 5 minutes at room temperature. Next, cells were blocked with 5% BSA for 30 minutes at 37°C, before being incubated with the indicated primary antibody overnight at 4°C. Cells were then washed with PBS and incubated with secondary antibody (Jackson Immunoresearch or Biotium, 1:1000 dilution) for 30 minutes at 37°C. Cells were then stored in 1 x PBS at 4°C until imaging.

### Virus filament isolation

Virus was subjected to centrifugation through a 5 μm filter (Millipore) at 5000 x g for 5 minutes at 4°C, then through a 0.45 μm filter (Millipore) at 5000 x g for 1 minute at 4°C to separate filaments from cell debris. Next, 35 mm glass bottom dishes (Mattek) were coated with poly-L-lysine (Sigma) for 1 h at room temperature. Filtered virus filaments were then centrifuged onto dish surfaces at 2400 x g for 30 minutes at 4°C. Filaments were then immunostained as described above for dSTORM.

### Protein-Protein Proximity Ligation Assays

At the indicated timepoints, specified cells in a 96 well glass bottom plate were fixed with 1% paraformaldehyde for 10 minutes at room temperature. Cells were then permeabilized with 0.2% Triton X-100 for 5 minutes at room temperature. Cells were then blocked for 30 minutes at 37°C in a blocking buffer of 1% BSA, 2% donkey serum, and 0.2% gelatin in 1X PBS. PLA was then performed according to manufacturer instructions. Briefly, cells were incubated with primary antibodies for 30 min at 30 minutes at 37°C. Primary antibodies were diluted in a buffer of 1% BSA, 0.2% donkey serum (Sigma Aldrich), and 0.25% gelatin (Thermo Fisher). Cells were washed with Duolink Wash Buffer A (Sigma Aldrich) for 10 minutes at room temperature and then incubated with PLA secondary antibodies (Sigma Aldrich) for 30 minutes at 37°C. Cells were then washed with Duolink Wash Buffer A for 10 minutes at room temperature and then incubated with PLA ligation reagents (Sigma Aldrich) for 30 minutes at 37°C. Next, cells were washed with Duolink Wash Buffer A for 10 minutes at room temperature and then incubated with PLA rolling circle amplification reagents (Sigma Aldrich) for 100 minutes at 37°C. Cells were then washed for 20 minutes at room temperature with Duolink Wash Buffer B (Sigma Aldrich) and then were immunostained as described above. Controls included no primary antibodies and mock infected cells. Cells were positively selected for successful infection by presence of viral protein immunofluorescence. Biological replicates were used, where n = 30 cells counted.

### Multiply-labeled Tetravalent RNA Imaging Probe (MTRIP) Assembly

A V5 epitope tag was conjugated to NeutrAvidin (ThermoFisher) with Solulink conjugation technology, according to manufacturer protocol (Trilink). V5-NeutrAvidin was incubated with 5’biotynlated peptide nucleic acid (PNA) oligonucleotides (PNA-Bio) at a 1:5 mol ratio for 1 hour at room temperature. Multiply-labeled tetravalent RNA imaging probes (MTRIPS) were then filtered with a 30 KDa MWCO centrifugal filter (Millipore Sigma) before use. PNA oligonucleotides (Table 1) were designed with a 5’ biotin and an O-K-O-K spacer following manufacturer design specifications.

**Table 1:** PNA oligonucleotide sequences.

Target Name	Accession Number/Target Sequence	PNA Sequence
Poly(A) Tail	AAAAAAAAAAAAA	TTTTTTTTTTTTT
RSV genome	M74568	ATGGGGCAAATA
RSV genome	M74568	ACACTTTTTTTCTC
RSV genome	M74568	AACTAATCCTAAAG
RSV genome	M74568	TGAACTAGGATATC
RSV NS1 mRNA	M74568	TTTGTTGTACGCATTTT
RSV NS1 mRNA	M74568	GTACTTATCAAATTCTT
RSV NS1 mRNA	M74568	GTATCACTGCCTTAGCC
RSV NS1 mRNA	M74568	GTTAGTGACATTGATTT
Scrambled	AACCCTGAAGCGACGA	TCGTCGCTTCAGGGTT

### RNA-Protein Proximity Ligation Assays (FISH-PLA)

FISH-PLA was performed as previously described [33]. Briefly, at the indicated timepoints, specified cells in a 96 well glass bottom plate were fixed with 1% paraformaldehyde for 10 minutes at RT. Cells were then permeabilized with 0.2% Triton X-100 for 5 minutes at RT. Cells were blocked for endogenous biotin with an endogenous biotin blocking kit (Thermo Fisher). MTRIPs were made as described above targeting RSV genome RNA, RSV NS1 mRNA, or polyadenylated transcripts. Controls included no primary antibodies, NeutrAvidin without a V5 epitope tag, NeutrAvidin bound to a scrambled PNA oligonucleotide, NeutrAvidin bound to biotin without an oligonucleotide, no FISH, and mock infected cells. MTRIPs were then incubated with cells overnight at 37°C in a hybridization buffer of 2x SSC with 0.5% tRNA, 0.5% ssDNA and 0.2% BSA in 1x PBS. RSV genome FISH, poly(A) FISH, and associated controls were at 5 nM, while NS1 mRNA FISH and associated controls were at 10 nM. PLA was then performed as described above between the V5 tag and RSV L. Cells were positively selected for successful infection by presence of viral protein immunofluorescence. Biological replicates were used, where n = 30 cells counted.

### *In vitro* transcription of mRNA

DNA gblocks (IDT DNA) were designed using the full-length nucleotide sequences of GFP and RSV M2-2 and RSV M2-1 (GenBank M74568.1). The sequences contained a 5’ untranslated region (UTR) with a Kozak sequence, a 3’ UTR derived from the mouse alpha globin sequence, and extensions that allow for Gibson assembly. RSV M2-2 mRNA contained a 5’ Myc tag sequence while RSV M2-1 contained a 5’ HA tag sequence (Table 2). Sequences were codon optimized and then inserted into a pMA-7 vector using Gibson assembly using New England Biolabs Builder. Plasmids were first linearized overnight at 37°C with Not-1 HF (New England Biolabs). They were then *in vitro* transcribed overnight at 37°C using a HiScribe T7 kit (New England Biolabs), capped, and polyadenylated enzymatically (Aldeveron). Nucleotides used were ATP, GTP, CTP and m1Y-5’-triphosphate (Trilink). mRNAs were then purified with a lithium chloride precipitation before treatment with Antarctic Phosphatase (New England Biolabs) for 2 h. They were then purified again before storage for use.

**Table 2:** IVT mRNA open reading frame sequences.

mRNA Name	ORF (5’-3’)
RSV M2-2-Myc	ATGGAACAAAAACTGATTTCTGAGGAGGACCTTGGAGGATCAGGCGGCTCCACCATGCCCAAGATTATGATTCTCCCAGACAAGTACCCCTGCAGTATTACGTCCATCTTGATTACCAGTAGATGTAGAGTCACGATGTATAACCAGAAAAACACGTTGTATTTCAACCAAAACAACCCAAACAACCATATGTATAGTCCGAACCAGACCTTCAATGAAATCCATTGGACATCTCAAGAACTGATCGATACAATTCAGAACTTTCTTCAACACCTCGGCATTATAGAAGATATATACACGATATACATCCTGGTTTCTTGA
RSV M2-1-HA	ATGTATCCTTATGACGTGCCCGATCACGCTTCCCGCCGGAACCCATGCAAATTTGAAATACGAGGTCATTGTCTCAATGGTAAGCGGTGTCATTTCTCACACAACTACTTTGAGTGGCCACCGCATGCACTGTTGGTTCGACAGAATTTCATGCTCAACCGAATCCTCAAATCTATGGATAAGAGCATCGACACGCTGAGCGAGATCTCTGGCGCGGCCGAACTTGACAGAACTGAGGAATATGCTCTCGGCGTCGTCGGCGTTCTCGAAAGCTACATAGGTTCCATAAATAACATCACAAAACAATCTGCCTGCGTAGCCATGAGCAAACTGCTCACGGAACTTAACTCAGATGATATTAAAAAGTTGAGAGACAATGAAGAGTTGAATTCACCAAAGATACGAGTATATAACACGGTTATCAGTTATATTGAATCAAATAGAAAGAACAACAAACAGACGATTCACCTGCTCAAACGGCTTCCGGCAGACGTGTTGAAGAAAACTATAAAGAACACCCTCGACATACATAAGTCTATAACAATAAACAACCCAAAGGAGTCCACAGTTTCAGACACTAACGATCACGCTAAAAATAACGACACTACATGATAA
GFP	ATGGTGTCCAAGGGCGAGGAACTGTTCACCGGCGTGGTGCCCATCCTGGTGGAACTGGATGGCGACGTGAACGGCCACAAGTTCAGCGTGTCCGGCGAGGGCGAAGGCGACGCCACATACGGAAAGCTGACCCTGAAGTTCATCTGCACCACCGGCAAGCTGCCCGTGCCTTGGCCTACCCTCGTGACCACACTGACCTACGGCGTGCAGTGCTTCAGCAGATACCCCGACCATATGAAGCAGCACGACTTCTTCAAGAGCGCCATGCCCGAGGGCTACGTGCAGGAAAGAACCATCTTCTTTAAGGACGACGGCAACTACAAGACCAGGGCCGAAGTGAAGTTCGAGGGCGACACCCTCGTGAACAGAATCGAGCTGAAGGGCATCGACTTCAAAGAGGACGGCAACATCCTGGGCCACAAGCTGGAGTACAACTACAACAGCCACAACGTGTACATCATGGCCGACAAGCAGAAAAACGGCATCAAAGTGAACTTCAAGATCCGGCACAACATCGAGGACGGCTCCGTGCAGCTGGCCGACCACTACCAGCAGAACACCCCTATCGGCGACGGCCCTGTGCTGCTGCCTGACAACCACTACCTGAGCACCCAGAGCGCCCTGAGCAAGGACCCCAACGAGAAGAGGGACCACATGGTGCTGCTGGAATTCGTGACCGCCGCTGGCATCACCCTGGGCATGGACGAGCTGTACAAGTGATAA

### Transfection of *in vitro* transcribed mRNA

Cells were transfected using lipofectamine 3000 (Thermo Fisher), according to manufacturer instructions. Cells were transfected with 200 ng RNA per well for 96 well plates, 500 ng RNA per well for 8 chambered glass slides, and 5 μg per well for 6 well plates to maintain consistent RNA concentration across different sized wells.

### RT-qPCR analysis of viral RNA levels

For relative quantification of N RNA in S5B Fig, A549 cells were infected with RSV A2 at an MOI of 3. At designated timepoints, total RNA was extracted from infected cells using RNeasy Mini Kit or TRIzol extraction as previously described. A total of 250 ng of RNA was reverse transcribed using SuperScript IV reverse transcriptase (Thermo Fisher) per manufacturer’s instructions with primers for either positive sense or negative sense N RNA and GAPDH (Table 3). cDNA was then combined with iTaq universal SYBR green supermix (BioRad), 300 nM of primers, and water added to a final volume of 20 μL, where cDNA represented 10% of the final qPCR mix. Relative RNA expression compared to RNA present at 5 hpi was performed with the 2^-ΔΔCt^ method and the linear regressions were performed by Prism. Experiment was performed in duplicate. For data presented in S8D Fig, Vero cells were plated in 6 well plates. Cells were then infected with RSV A2 at a MOI of 3. 3 hpi, cells were transfected with specified mRNAs or with a lipofectamine 3000 only control. Biological duplicates were performed. At designated timepoints, total RNA was extracted from infected cells using a RNeasy Plus kit (Qiagen) following manufacturer instructions. mRNA was then isolated using an Oliogotex Kit (Qiagen) following manufacturer instructions. Next, cDNA was synthesized from mRNA using equivalents amount of mRNA for each condition with a High Capacity cDNA kit (Applied Biosystems), also following manufacturer instructions. RT-qPCR was then performed in technical triplicate for each biological duplicate using TaqMan probes (Thermo Fisher) for RSV F mRNA and GAPDH (Table 3).

**Table 3:** RT-qPCR primers.

Oligonucleotide	Source	Identifier
RSV N-qPCR Forward: ATGGGAGAGGTAGCTCCAGA	ThermoFisher	This study
RSV N-qPCR Reverse: AGCTCTCCTAATCACGGCTG	ThermoFisher	This study
GAPDH qPCR Forward (S5B Fig): TGGTCACCAGGGCTGCTT	ThermoFisher	RTPrimerDB # 1233
GAPDH qPCR Reverse (S5B Fig): AGCTTCCCGTTCTCAGCCTT	ThermoFisher	RTPrimerDB # 1233
Taqman Probes: GAPDH (S8D Fig)	ThermoFisher	Cat# 4331182; Assay ID: Hs02786624_g1
RSV F- qPCR Forward: AACAGATGTAAGCAGCTCCGTTATC	ThermoFisher	Mentel et al. 2003
RSV F-qPCR Reverse: CGATTTTTATTGGATGCTGTACATTT	ThermoFisher	Mentel et al. 2003
RSV F Probe: TGCCATAGCATGACACAATGGCTCCT	ThermoFisher	Mentel et al. 2003

### Confocal microscopy

Images were acquired with a Hamamatsu Flash 4.0 v2 sCMOS camera on a PerkinElmer UltraView spinning disk confocal microscope mounted to a Zeiss Axiovert 200 M body with a x63 NA 1.4 plan-apochromat objective. Images were acquired with Volocity Acquisition Software (PerkinElmer) with z-stack intervals of 200 nm. Images were linearly contrast enhanced for visual clarity. Images were uniformly contrast enhanced across an experiment.

### dSTORM analysis

1xPBS was removed from cells and replaced with dSTORM imaging buffer of 50 mM Tris-HCl (pH 8.0), 10 mM NaCl, 10% glucose, 20 mM cysteamine, 1% 2-Mercaptoethanol, 169 AU/mL glucose-oxidase, and 1404 AU/mL catalase. Buffer was made fresh for use and replaced after 2 hours. Images were acquired using a Bruker Vutara 352 with a 63x NA 1.2 plan-apochromat water immersion objective lens at RT. Vutara SRX software was used for acquisition and analysis.

### Image analysis

All quantifications and analyses were performed on unenhanced data. PLA image analysis and protein volume measurements were performed using Volocity acquisition software. Regions of interest (ROI) were drawn around individual cells that demonstrated positive viral signal and/or exogenous overexpressed protein signal. PLA puncta number and volume, as well as viral protein volume, were quantified per cell above a specified signal threshold. Signal thresholds were kept consistent within each experiment. dSTORM images were analyzed using Vutara SRX software. ROIs were drawn around each RSV granule. Cluster analysis by Vutara SRX software found the (x,y,z) location of the centroid of each protein cluster in the granule. The distance between the two protein clusters was then found.

### Statistical analysis

Statistics were performed in GraphPad Prism 7.0. Statistical tests used, n values, error bars, and significance levels for each experiment can be found in the figure legends and methods. When data is displayed as per cell values, mean value is also represented as a line with precision measurements. Significance was defined as p < 0.05, but specific significances were defined for each experiment in the figure legends. Power analysis was performed to determine adequate sample size. Cells were picked for inclusion on the basis of positive staining for infection and/or transfection, depending on the experiment. A full list of p-values can be seen in S1 Table.

## Supporting information

S1 Fig Recombinant RSV expressed epitope-tagged L protein replicates efficiently. **A)** Northern blot analysis of genome RNA and N mRNA produced by recombinant RSV (rRSV) or recombinant RSV containing the flag(2) tag (rRSV-flag2-L) at various times post infection (hpi). Mock-infected cells are denoted by ‘M’. **B)** Western blot for flag tag expressing L protein of total cell lysate from Mock, rRSV, or rRSVflag(2)-L infected cells. **C)** Growth curves of rRSV and rRSV-flag2-L viruses, with n = 3.
(TIF)

S2 Fig Localization of RSV L, P and M2-1 proteins in inclusion bodies, filaments and assembly granules. **A)** A549 cells were infected or mock infected with rRSVflag(2)L at a MOI of 3. Cells were fixed at 1, 6, 12, and 24 hours post infection. Cells were stained for RSV L (green), RSV P (red) and RSV F (blue). Single plane images are shown. Arrowheads indicate incoming virus particles, while arrows indicate viral protein granules. Scale bar is 10 μm. **B)** Enlarged cropped images of viral structures indicated by white boxes in (A). Single plane images are shown. Intensity profiles are drawn along the white line on the cropped image. **C)** A549 cells were infected or mock infected with rRSVflag(2)L at a MOI of 3. Cells were fixed at 1, 6, 12, and 24 hours post infection. Cells were stained for RSV L (green), RSV M2-1 (red) and RSV F (blue). Single plane images are shown. Arrowheads indicate viral protein granules. Scale bar is 10 μm. **D)** Enlarged cropped images of viral structures indicated by white boxes in (C). Single plane images are shown. Intensity profiles are drawn along the white line on the cropped image.
(TIF)

S3 Fig Additional timepoints for viral protein PLA. **A)** A549 cells were infected or mock infected with rRSVflag(2)L at a MOI of 3. Cells were fixed at 1, 6, 8, 12, 18, and 24 hours post infection. PLA (white) was performed between N-L, P-L, and M2-1-L. Cells were stained for RSV F (green) to select for positively infected cells. Representative extended images for additional timepoints from Fig 3 are shown. Duplicates were performed, but representative images from one experiment are shown. Scale bar is 10 μm. **B)** Additional quantification of PLA experiments described in Fig 3. Mean values and 95% confidence intervals are shown in grey. A two-way ANOVA with a Tukey’s multiple comparison test was performed, where n = 30 and * p < 0.05, *** p < 0.001, and **** p < 0.0001. **C)** Quantification of RSV F or panRSV protein volume over time, with mean values and 95% confidence intervals. Linear regression of the log transform of volumes is shown on right. Statistics were an ANCOVA, and no significant difference between the slopes was found, with n = 100 cells.
(TIF)

S4 Fig RSV proteins disassociate from the incoming virion early in infection. **A)** A549 cells were infected or mock infected with rRSVflag(2)L at a MOI of 3. Cells were fixed at 1, 6, and 8 hours post infection. PLA (white) was performed between F-L. Cells were stained for panRSV (green) to select for positively infected cells. Duplicates were performed, but representative extended focus images from one experiment are shown. Scale bar is 10 μm. **B)** Quantification for PLA in (A) is shown. PLA volume was normalized by volume of panRSV in the cell. Mean and 95% confidence intervals are shown in grey. Two-way ANOVAs with a Tukey’s multiple comparison test were performed, where n = 30 cells and **** p < 0.0001.
(TIF)

S5 Fig RSV RNA quantities increases over time, with an increase in synthesis post 8 hpi. A549 cells were infected or mock infected with rRSVflag(2)L at a MOI of 3. RNA was harvested at 5, 6, 7, 8, and 9 hours post infection. **A)** Northern blots were performed for the positive and negative sense RSV N RNA. **B)** RT-qPCR for the negative and positive sense RSV N RNA was performed. The slopes of the data from 4–8 hpi and 8–9 hpi were determined by linear regression and are shown on the graphs with SEM for n = 2.
(TIF)

S6 Fig Additional timepoints for RSV genome-L PLA. A549 cells were infected or mock infected with rRSVflag(2)L at a MOI of 3. Cells were fixed at 6, 8, 12, 18, and 24 hours post infection. FISH with PMTRIPs was performed for RSV genome, a scrambled control, or without targeting oligos. PLA (white) was performed between RSV genome RNA and L protein. Cells were stained for panRSV (green) to select for positively infected cells. Duplicates were performed, but representative extended focus images from one experiment are shown. **A)** Additional control images for Fig 4. Scale bar is 10 μm. **B)** Images for additional timepoints related to the results described in Fig 4. Scale bar is 10 μm. **C)** Quantification of PLA with all timepoints described in Fig 4 for RSV genome-L PLA. PLA volume was normalized by volume of panRSV in the cell. Mean and 95% confidence intervals are shown in grey. Two-way ANOVAs with a Tukey’s multiple comparison test were performed, where n = 30 cells and *p < 0.05, *** p < 0.001 and **** p < 0.0001.
(TIF)

S7 Fig Additional timepoints and controls for NS1 mRNA-L PLA. A549 cells were infected or mock infected with rRSVflag(2)L at a MOI of 3. Cells were fixed at 6, 8, 12, 18, and 24 hours post infection. FISH with PMTRIPs was performed for NS1 mRNA (A) poly(A) (C), a scrambled control, or without targeting oligos. PLA (white) was performed between NS1 RNA (A) and L or poly(A) (C) and L. Cells were stained for panRSV (green) to select for positively infected cells. **A)** Images for additional timepoints from Fig 4, with a scale bar of 10 μm. **B)** Quantification of PLA with all timepoints from data presented in Fig 4 for RSV NS1 mRNA—L PLA. PLA volume was normalized by volume of panRSV in each cell. Mean and 95% confidence intervals are shown in grey. Two-way ANOVAs with a Tukey’s multiple comparison test were performed, where n = 30 cells and **** p < 0.0001. **C)** Extended focus images for poly(A)-L PLA. Duplicates were performed, but representative images from one experiment are shown. Representative images of additional controls are reported in S4 Fig. Scale bar is 10 μm. **D)** Quantification of PLA in (C). PLA volume was normalized by volume of panRSV in each cell. Mean and 95% confidence intervals are shown in grey. Two-way ANOVAs with a Tukey’s multiple comparison test (right) were performed, where n = 30 cells, * p < 0.05, ** p < 0.01, ***p < 0.001 and **** p < 0.0001. E) Comparison of the PLA volumes for NS1-L and poly(A)-L normalized over time. Mean and 95% confidence intervals are shown. A two-way ANOVA with a Sidak’s multiple comparison test was performed, with no significant difference between the two PLAs found.
(TIF)

S8 Fig M2-2 overexpression early in infection inhibits virus but does not affect FLAG epitope antibody access. **A)** Vero cells were infected with rRSVflag(2)L at a MOI of 3 and fixed at 6 or 8 hpi. Cells were stained for L (yellow) or N (magenta) and ribonucleocapsid complexes imaged with dSTORM. XY, XY, and YZ views are shown, with a 200 nm scale bar. XYZ positions of the centroid of the L and N protein clusters were measured, and the distance between L and N were quantified. Mean and 95% confidence intervals are shown in grey. 5 infected cells were imaged for each timepoint across two infections. N-L distance in Vero cells was compared to A549 cells as described in Fig 3. A two-way ANOVA with Tukey’s multiple comparison test was performed, where n = 40 granules for Veros and n = 10 granules for A549 cells; * p < 0.05 and **** p < 0.0001. No significance difference was found between the cell types. **B)** Control images for Fig 5A. Vero cells were transfected with 200 ng of GFP or M2-2-Myc mRNA 24 h pre-infection, 4 hpi, or 10 hpi. Cells were infected with RSV at a MOI of 3 or mock infected and fixed at 24 hpi. Cells were stained for GFP (green) or M2-2 via Myc (green) and panRSV (red). Single plane images are shown, with a scale bar of 10 μm. **C)** Vero cells in a 96 well plate were infected with RSV at a MOI of 3 and transfected with 200 ng, 100 ng, 50 ng, or 0 ng of M2-2-Myc or M2-1-HA mRNA at 3 hpi. At 24 hpi, the supernatant titer was measured via plaque assay. Mean and standard deviations are displayed in black. Statistics were a 2-way ANOVA with a Tukey’s multiple comparison test, where n = 4 and * p < 0.05, ** p <0.01, and **** p < 0.0001. **D)** Vero cells were transfected with 200 ng of GFP or M2-2 mRNA 4 hpi. Cells were infected with RSV at a MOI of 3 or mock infected and RNA was extracted at 6, 8, and 12 hpi. RSV F mRNA expression was analyzed by RT-qPCR. Statistics were a two-way ANOVA with a Tukey’s multiple comparisons test, where n = 2 and * p < 0.05. Standard deviations are shown in black. **E)** Vero cells were infected with rRSVflag(2)L at a MOI of 3. At 3 hpi, cells were transfected with 200 ng of M2-2-Myc mRNA or mock transfected. At 6 hpi, cells were fixed and immunostained for M2-2 via Myc (blue), L (red), or pan-RSV (green). Extended focus images are shown, with a 10 μm scale bar. **F)** Cropped images of granules as indicated by white boxes in (E). Line profiles are drawn along the white arrow in the image. L is shown in red and pan-RSV in green.
(TIF)

S9 Fig Additional images and controls for RNA-L PLA after M2-2 expression. Images for additional controls and timepoints for Fig 7. Vero cells were infected or mock infected with rRSVflag(2)L at a MOI of 3. Cells were transfected with 200 ng of M2-2-Myc or M2-1-HA mRNA at 3 hpi. **A-B)** Cells were fixed at 6 (A) or 8 (B) hours post infection. FISH with PMTRIPs was performed for RSV genome, RSV NS1 mRNA, without an epitope tag, a scrambled control, or without targeting oligos. PLA (white) was performed between RSV genome/ NS1 mRNA and L. Cells were stained for M2-2 via Myc (green) or M2-1 via HA (green), and F (red). Duplicates were performed, but representative extended focus images from one experiment are shown. Scale bar is 10 μm. **C)** Additional quantification for Fig 7, with mean and 95% confidence intervals in grey. Statistics were two-way ANOVAs with Tukey’s multiple comparison tests where n = 30 cells and * p < 0.05, ** p < 0.01, *** p < 0.001, and **** p < 0.0001. Quantification of PLA volume (right) is normalized by volume of RSV F per cell.
(TIF)

S1 Table Complete set of P values. (PDF)
