# Supplementary figures and images for: Polymerase-tagged respiratory syncytial virus reveals a dynamic rearrangement of the ribonucleocapsid complex during infection
Source: PLoS Pathog. 2020 Oct 8;16(10):e1008987. doi: 10.1371/journal.ppat.1008987 (PMC7575074; doi:10.1371/journal.ppat.1008987)

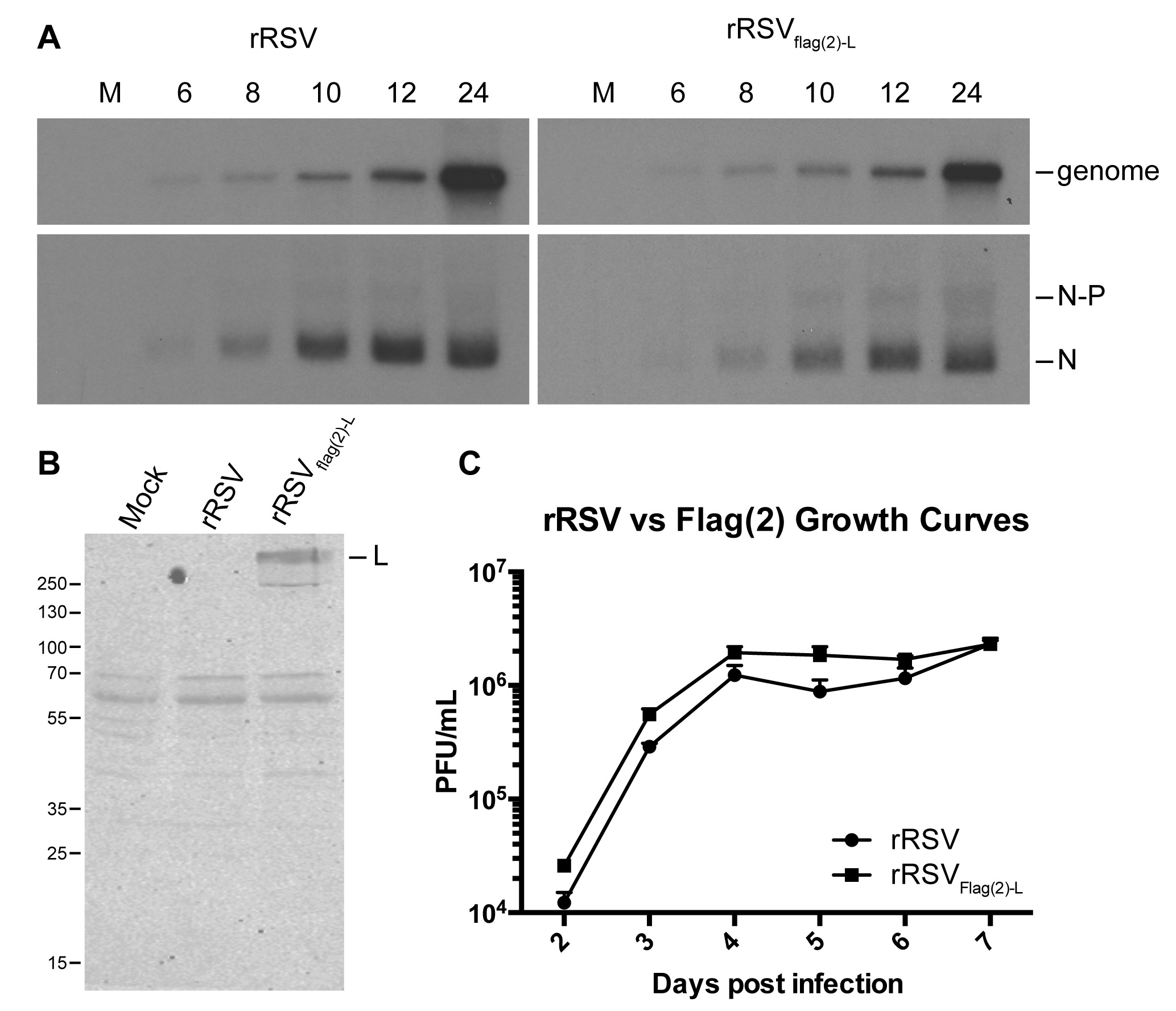

Supplement: S1 Fig — A) Northern blot analysis of genome RNA and N mRNA produced by recombinant RSV (rRSV) or recombinant RSV containing the flag(2) tag (rRSV-flag2-L) at various times post infection (hpi). Mock-infected cells are denoted by ‘M’. B) Western blot for flag tag expressing L protein of total cell lysate from Mock, rRSV, or rRSVflag(2)-L infected cells. C) Growth curves of rRSV and rRSV-flag2-L viruses, with n = 3. (TIF) [file ppat.1008987.s001.tif]

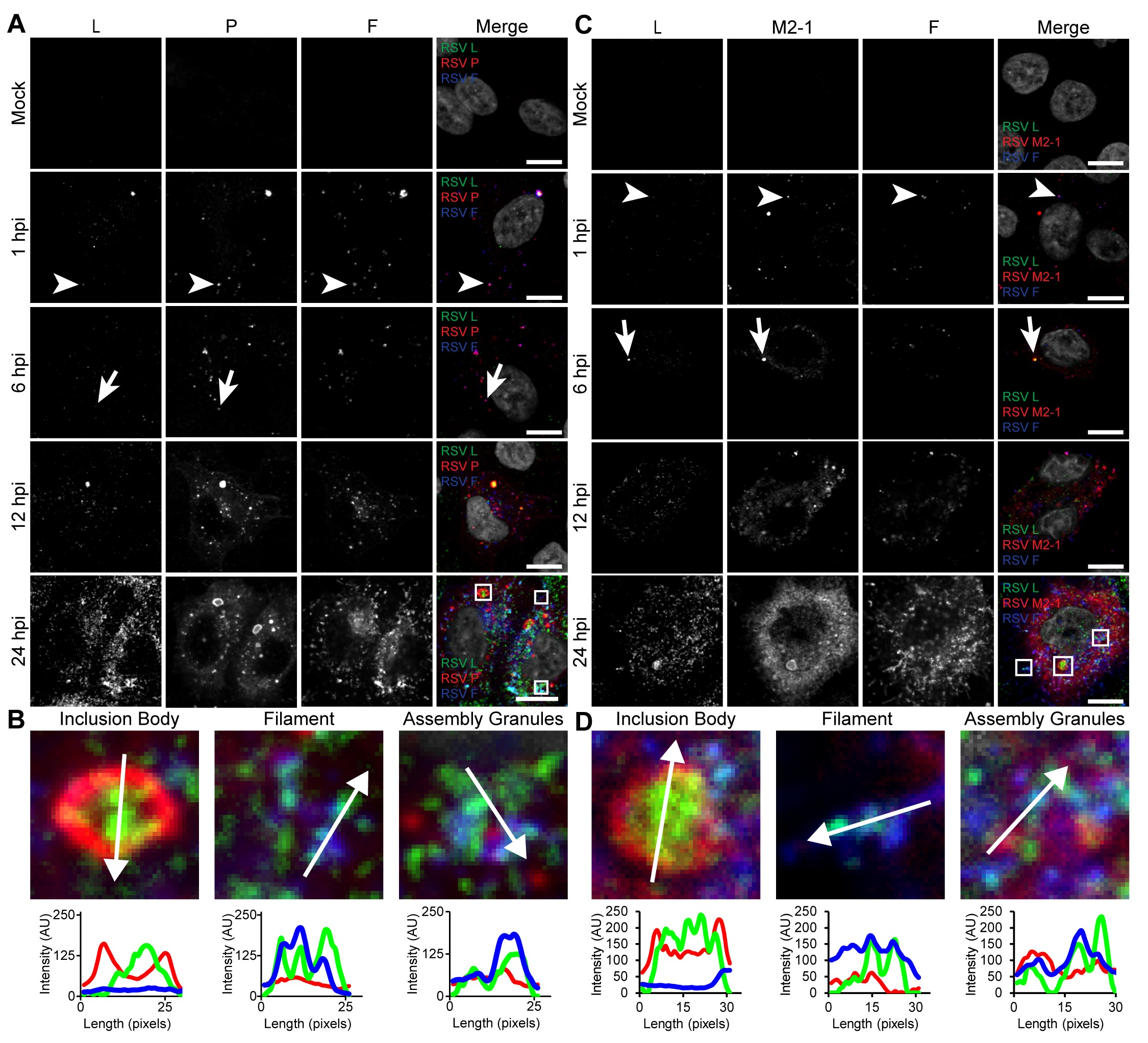

Supplement: S2 Fig — A) A549 cells were infected or mock infected with rRSVflag(2)L at a MOI of 3. Cells were fixed at 1, 6, 12, and 24 hours post infection. Cells were stained for RSV L (green), RSV P (red) and RSV F (blue). Single plane images are shown. Arrowheads indicate incoming virus particles, while arrows indicate viral protein granules. Scale bar is 10 μm. B) Enlarged cropped images of viral structures indicated by white boxes in (A). Single plane images are shown. Intensity profiles are drawn along the white line on the cropped image. C) A549 cells were infected or mock infected with rRSVflag(2)L at a MOI of 3. Cells were fixed at 1, 6, 12, and 24 hours post infection. Cells were stained for RSV L (green), RSV M2-1 (red) and RSV F (blue). Single plane images are shown. Arrowheads indicate viral protein granules. Scale bar is 10 μm. D) Enlarged cropped images of viral structures indicated by white boxes in (C). Single plane images are shown. Intensity profiles are drawn along the white line on the cropped image. (TIF) [file ppat.1008987.s002.tif]

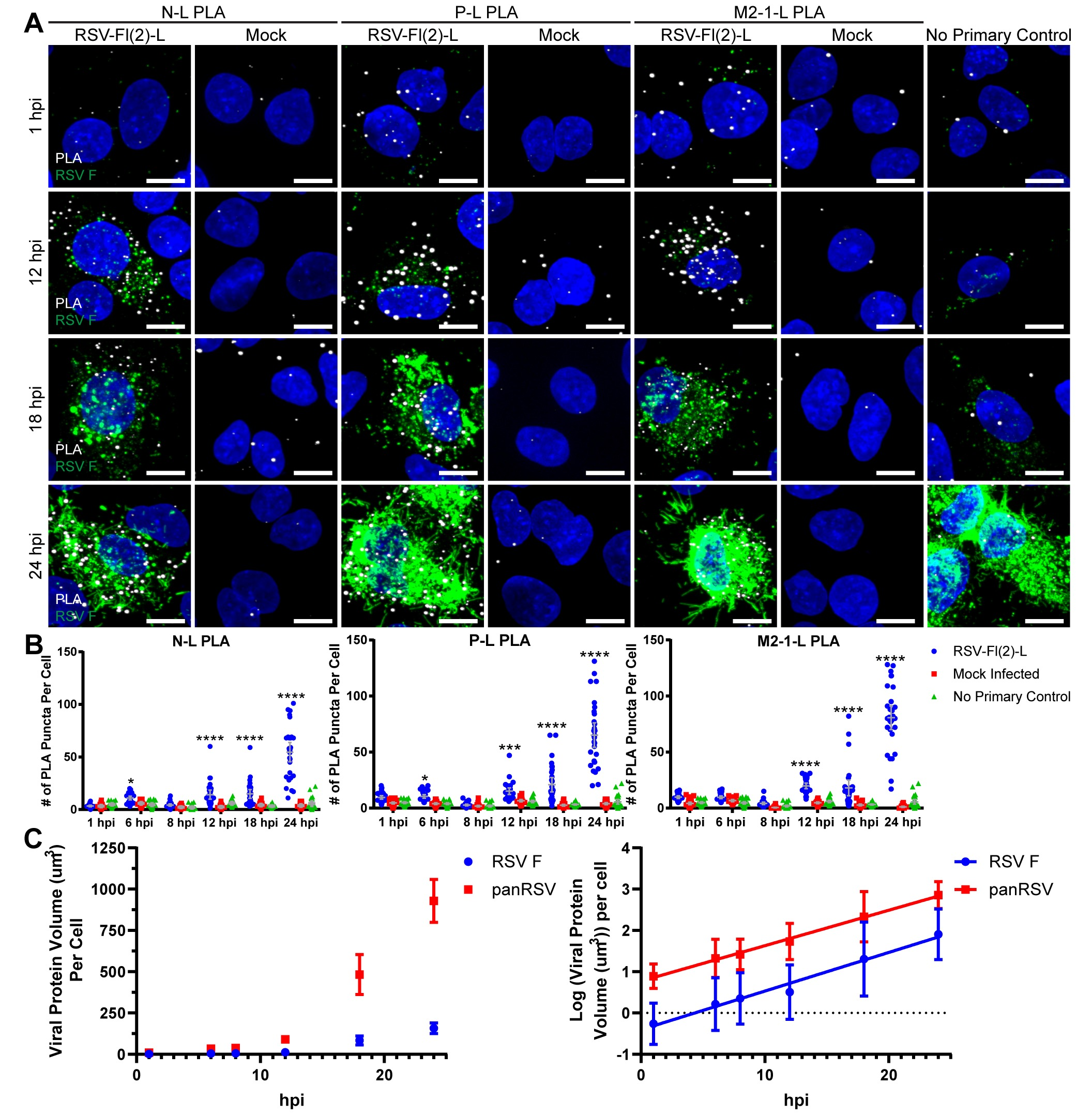

Supplement: S3 Fig — A) A549 cells were infected or mock infected with rRSVflag(2)L at a MOI of 3. Cells were fixed at 1, 6, 8, 12, 18, and 24 hours post infection. PLA (white) was performed between N-L, P-L, and M2-1-L. Cells were stained for RSV F (green) to select for positively infected cells. Representative extended images for additional timepoints from Fig 3 are shown. Duplicates were performed, but representative images from one experiment are shown. Scale bar is 10 μm. B) Additional quantification of PLA experiments described in Fig 3. Mean values and 95% confidence intervals are shown in grey. A two-way ANOVA with a Tukey’s multiple comparison test was performed, where n = 30 and * p < 0.05, *** p < 0.001, and **** p < 0.0001. C) Quantification of RSV F or panRSV protein volume over time, with mean values and 95% confidence intervals. Linear regression of the log transform of volumes is shown on right. Statistics were an ANCOVA, and no significant difference between the slopes was found, with n = 100 cells. (TIF) [file ppat.1008987.s003.tif]

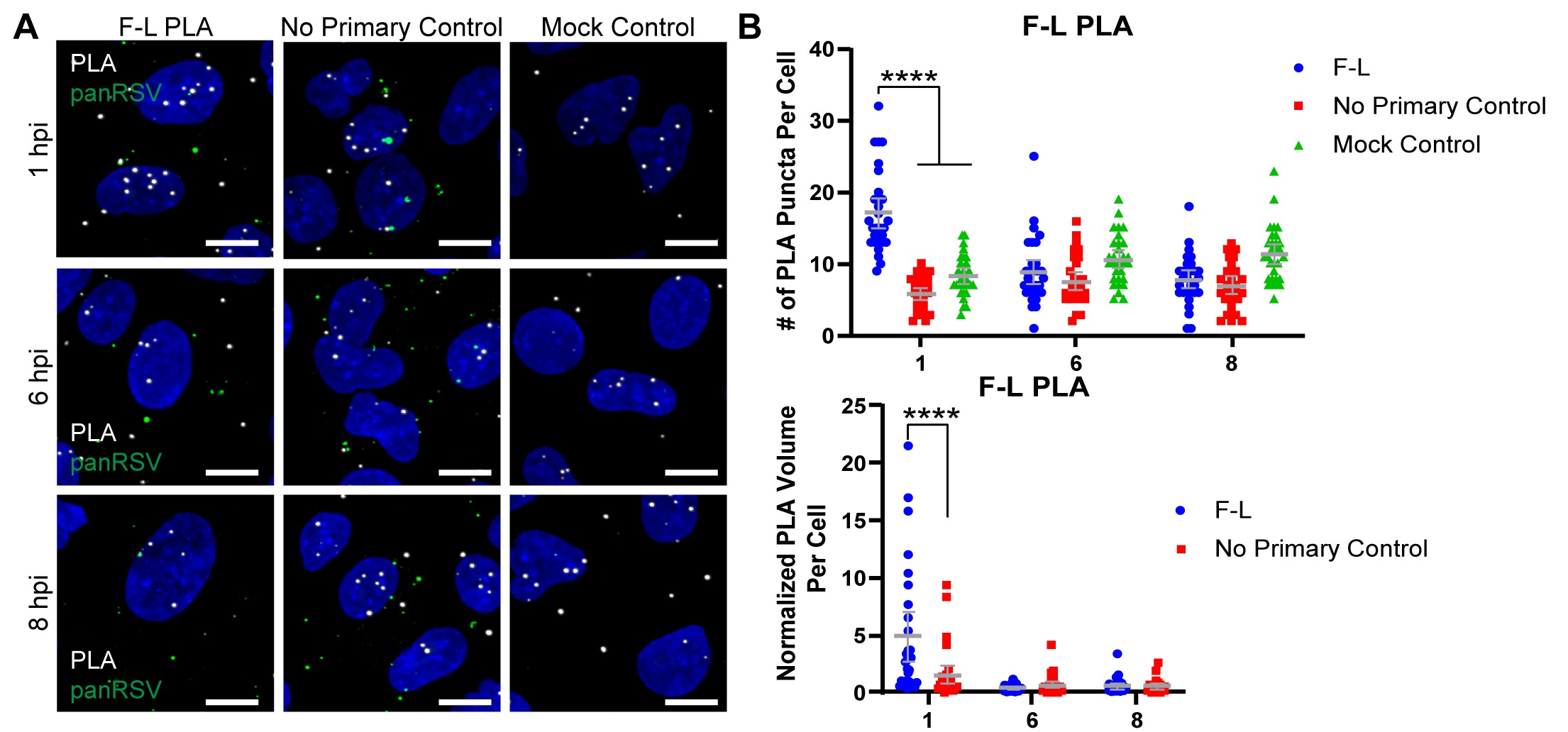

Supplement: S4 Fig — A) A549 cells were infected or mock infected with rRSVflag(2)L at a MOI of 3. Cells were fixed at 1, 6, and 8 hours post infection. PLA (white) was performed between F-L. Cells were stained for panRSV (green) to select for positively infected cells. Duplicates were performed, but representative extended focus images from one experiment are shown. Scale bar is 10 μm. B) Quantification for PLA in (A) is shown. PLA volume was normalized by volume of panRSV in the cell. Mean and 95% confidence intervals are shown in grey. Two-way ANOVAs with a Tukey’s multiple comparison test were performed, where n = 30 cells and **** p < 0.0001. (TIF) [file ppat.1008987.s004.tif]

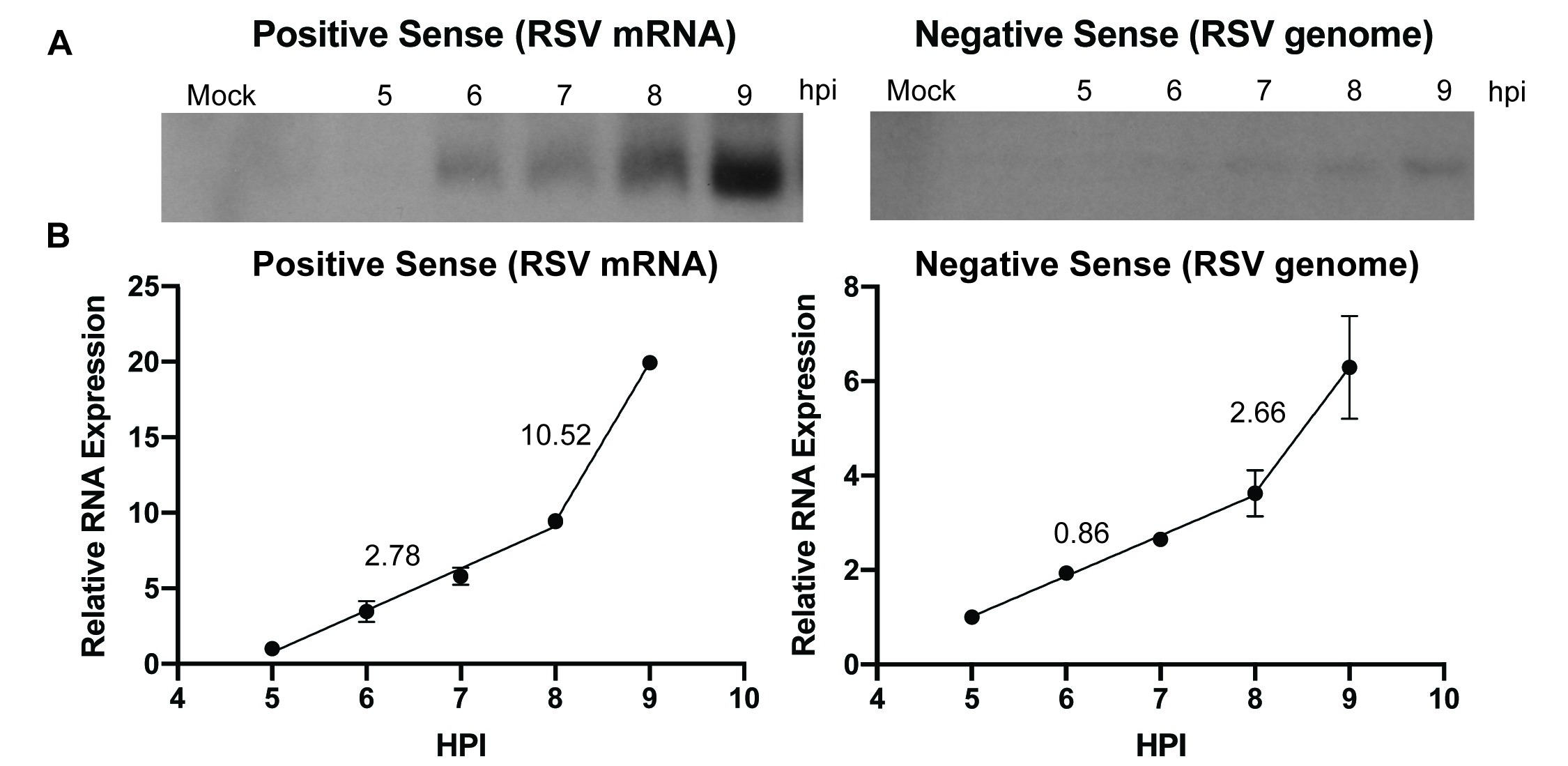

Supplement: S5 Fig — A549 cells were infected or mock infected with rRSVflag(2)L at a MOI of 3. RNA was harvested at 5, 6, 7, 8, and 9 hours post infection. A) Northern blots were performed for the positive and negative sense RSV N RNA. B) RT-qPCR for the negative and positive sense RSV N RNA was performed. The slopes of the data from 4–8 hpi and 8–9 hpi were determined by linear regression and are shown on the graphs with SEM for n = 2. (TIF) [file ppat.1008987.s005.tif]

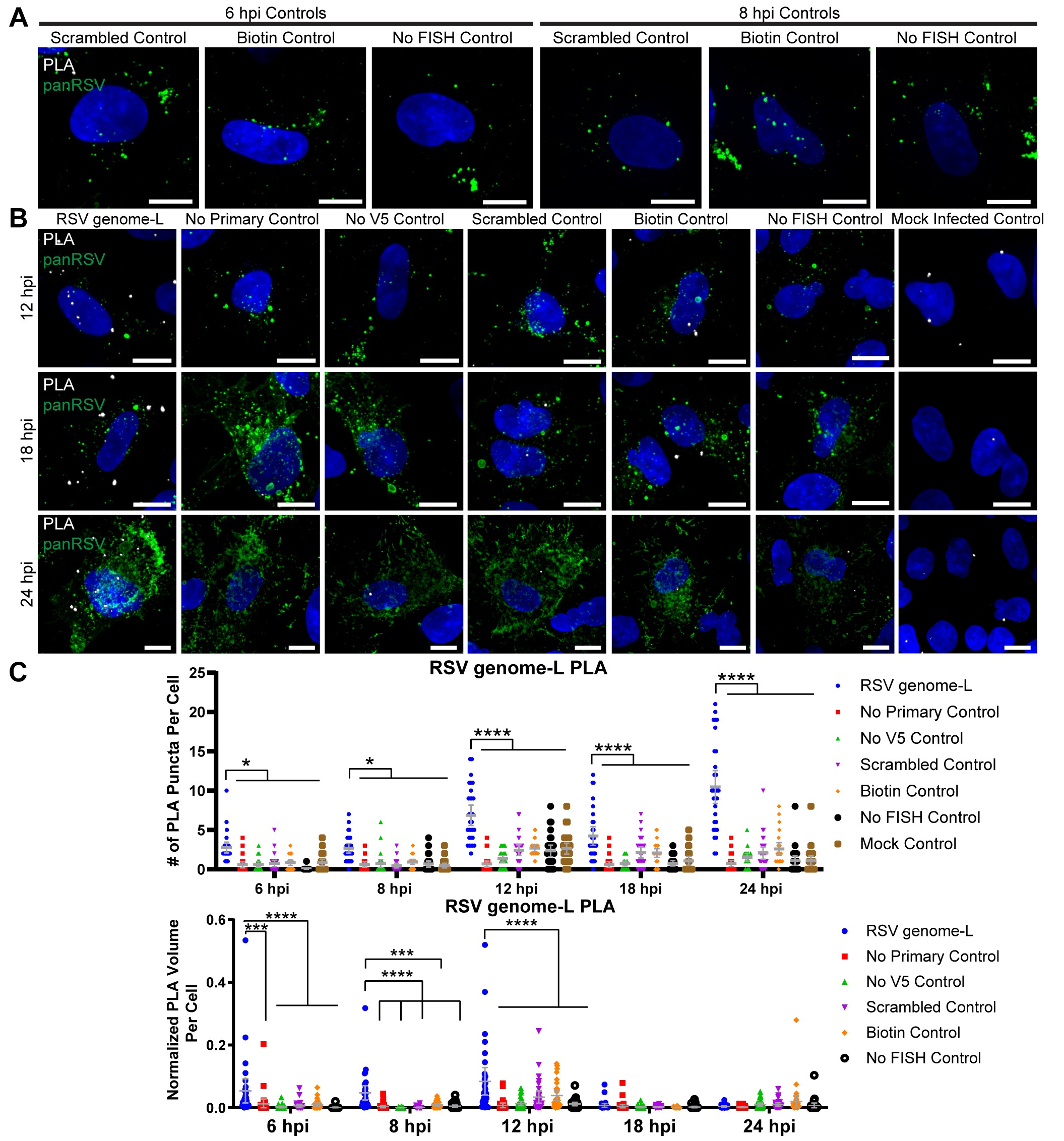

Supplement: S6 Fig — A549 cells were infected or mock infected with rRSVflag(2)L at a MOI of 3. Cells were fixed at 6, 8, 12, 18, and 24 hours post infection. FISH with PMTRIPs was performed for RSV genome, a scrambled control, or without targeting oligos. PLA (white) was performed between RSV genome RNA and L protein. Cells were stained for panRSV (green) to select for positively infected cells. Duplicates were performed, but representative extended focus images from one experiment are shown. A) Additional control images for Fig 4. Scale bar is 10 μm. B) Images for additional timepoints related to the results described in Fig 4. Scale bar is 10 μm. C) Quantification of PLA with all timepoints described in Fig 4 for RSV genome-L PLA. PLA volume was normalized by volume of panRSV in the cell. Mean and 95% confidence intervals are shown in grey. Two-way ANOVAs with a Tukey’s multiple comparison test were performed, where n = 30 cells and *p < 0.05, *** p < 0.001 and **** p < 0.0001. (TIF) [file ppat.1008987.s006.tif]

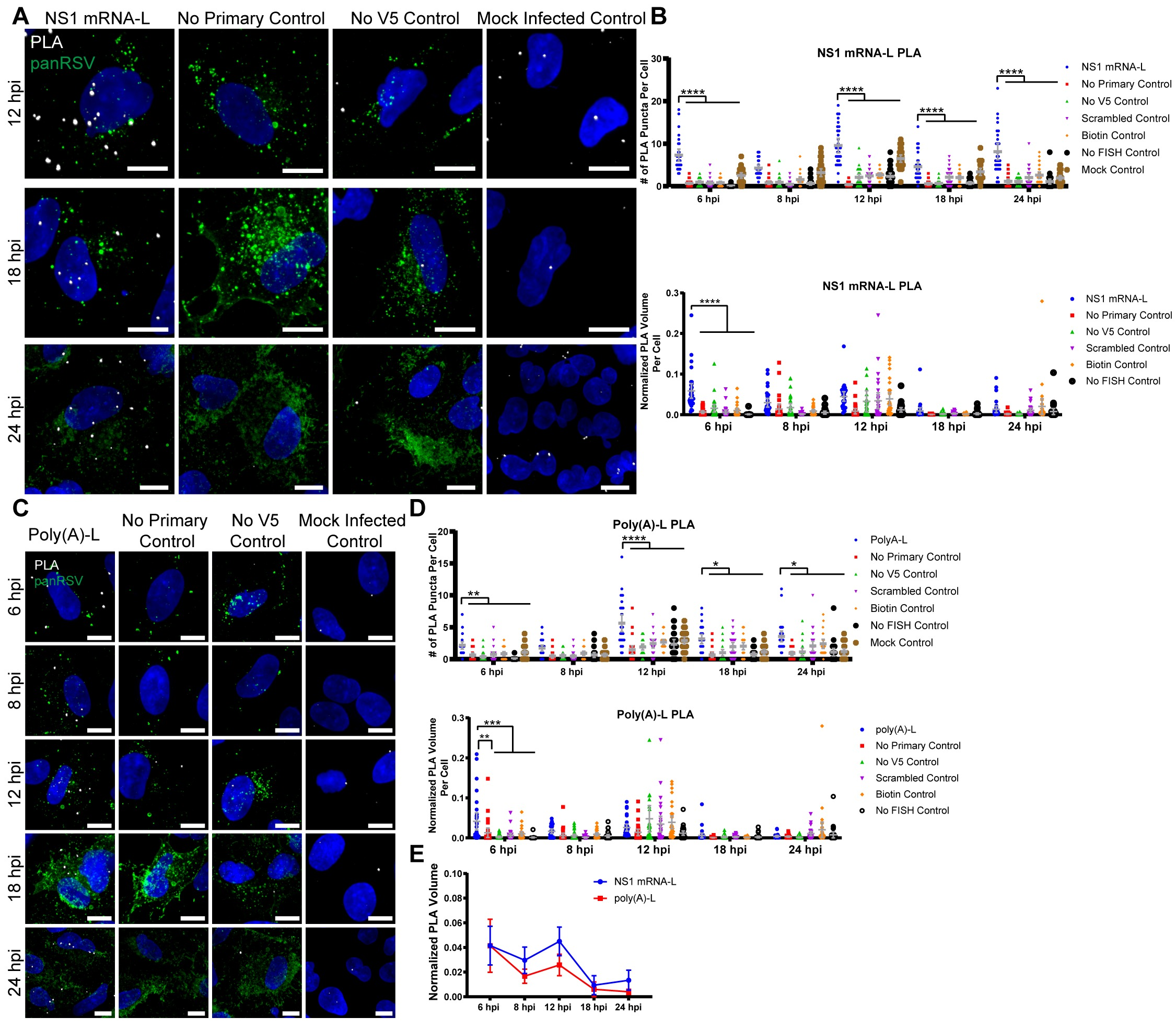

Supplement: S7 Fig — A549 cells were infected or mock infected with rRSVflag(2)L at a MOI of 3. Cells were fixed at 6, 8, 12, 18, and 24 hours post infection. FISH with PMTRIPs was performed for NS1 mRNA (A) poly(A) (C), a scrambled control, or without targeting oligos. PLA (white) was performed between NS1 RNA (A) and L or poly(A) (C) and L. Cells were stained for panRSV (green) to select for positively infected cells. A) Images for additional timepoints from Fig 4, with a scale bar of 10 μm. B) Quantification of PLA with all timepoints from data presented in Fig 4 for RSV NS1 mRNA—L PLA. PLA volume was normalized by volume of panRSV in each cell. Mean and 95% confidence intervals are shown in grey. Two-way ANOVAs with a Tukey’s multiple comparison test were performed, where n = 30 cells and **** p < 0.0001. C) Extended focus images for poly(A)-L PLA. Duplicates were performed, but representative images from one experiment are shown. Representative images of additional controls are reported in S4 Fig. Scale bar is 10 μm. D) Quantification of PLA in (C). PLA volume was normalized by volume of panRSV in each cell. Mean and 95% confidence intervals are shown in grey. Two-way ANOVAs with a Tukey’s multiple comparison test (right) were performed, where n = 30 cells, * p < 0.05, ** p < 0.01, ***p < 0.001 and **** p < 0.0001. E) Comparison of the PLA volumes for NS1-L and poly(A)-L normalized over time. Mean and 95% confidence intervals are shown. A two-way ANOVA with a Sidak’s multiple comparison test was performed, with no significant difference between the two PLAs found. (TIF) [file ppat.1008987.s007.tif]

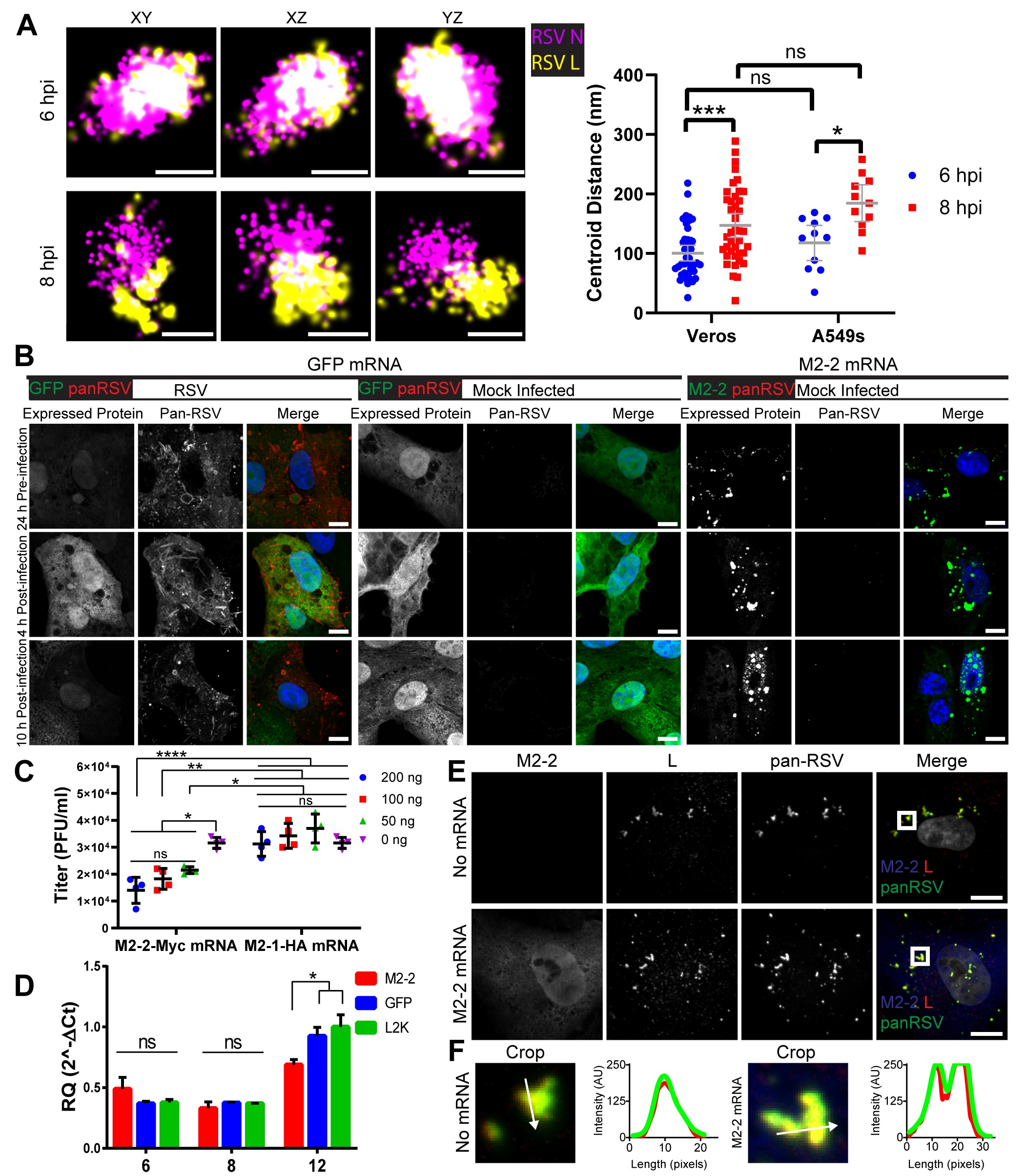

Supplement: S8 Fig — A) Vero cells were infected with rRSVflag(2)L at a MOI of 3 and fixed at 6 or 8 hpi. Cells were stained for L (yellow) or N (magenta) and ribonucleocapsid complexes imaged with dSTORM. XY, XY, and YZ views are shown, with a 200 nm scale bar. XYZ positions of the centroid of the L and N protein clusters were measured, and the distance between L and N were quantified. Mean and 95% confidence intervals are shown in grey. 5 infected cells were imaged for each timepoint across two infections. N-L distance in Vero cells was compared to A549 cells as described in Fig 3. A two-way ANOVA with Tukey’s multiple comparison test was performed, where n = 40 granules for Veros and n = 10 granules for A549 cells; * p < 0.05 and **** p < 0.0001. No significance difference was found between the cell types. B) Control images for Fig 5A. Vero cells were transfected with 200 ng of GFP or M2-2-Myc mRNA 24 h pre-infection, 4 hpi, or 10 hpi. Cells were infected with RSV at a MOI of 3 or mock infected and fixed at 24 hpi. Cells were stained for GFP (green) or M2-2 via Myc (green) and panRSV (red). Single plane images are shown, with a scale bar of 10 μm. C) Vero cells in a 96 well plate were infected with RSV at a MOI of 3 and transfected with 200 ng, 100 ng, 50 ng, or 0 ng of M2-2-Myc or M2-1-HA mRNA at 3 hpi. At 24 hpi, the supernatant titer was measured via plaque assay. Mean and standard deviations are displayed in black. Statistics were a 2-way ANOVA with a Tukey’s multiple comparison test, where n = 4 and * p < 0.05, ** p <0.01, and **** p < 0.0001. D) Vero cells were transfected with 200 ng of GFP or M2-2 mRNA 4 hpi. Cells were infected with RSV at a MOI of 3 or mock infected and RNA was extracted at 6, 8, and 12 hpi. RSV F mRNA expression was analyzed by RT-qPCR. Statistics were a two-way ANOVA with a Tukey’s multiple comparisons test, where n = 2 and * p < 0.05. Standard deviations are shown in black. E) Vero cells were infected with rRSVflag(2)L at a MOI of 3. At 3 hpi, cells were [file ppat.1008987.s008.tif]

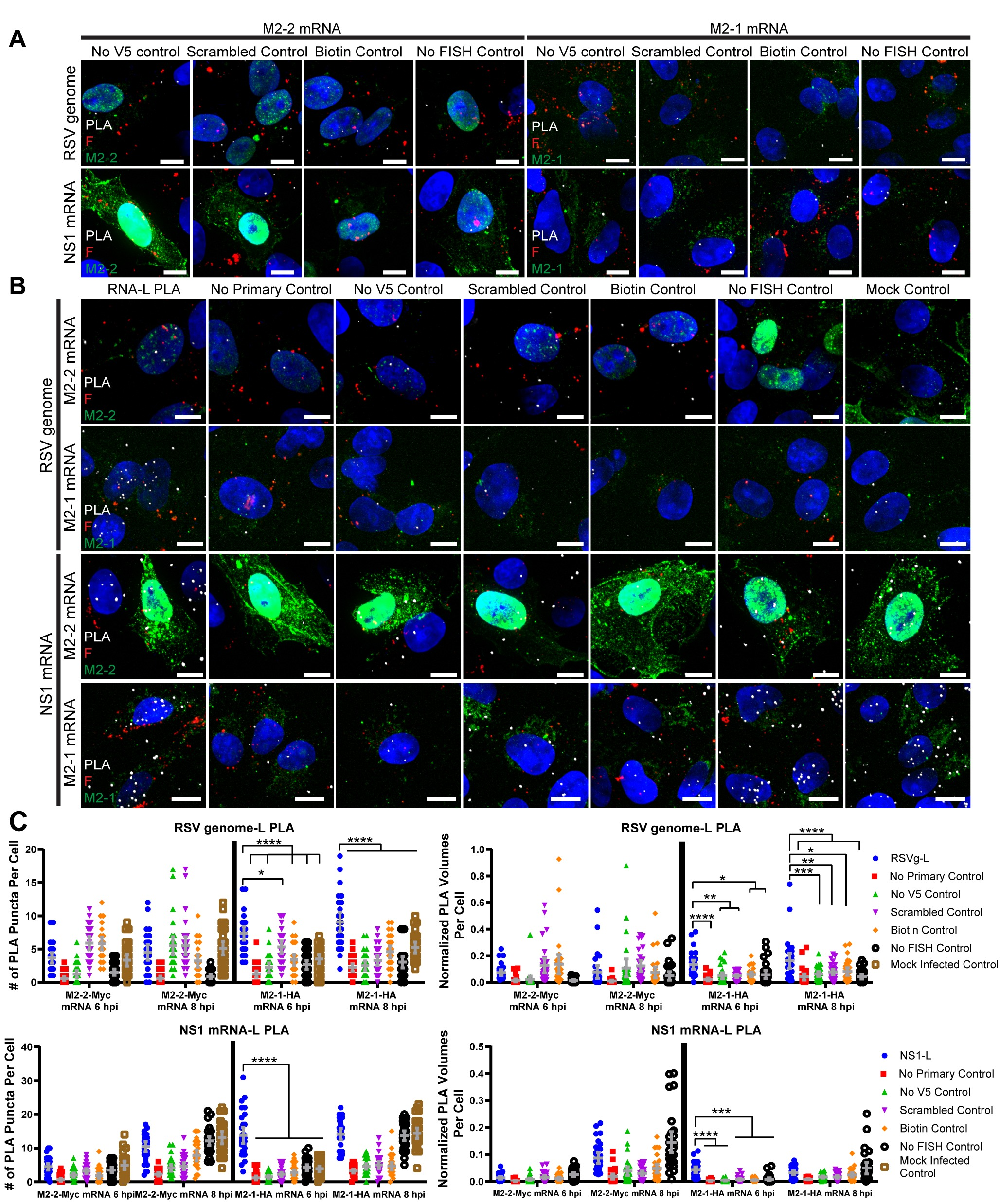

Supplement: S9 Fig — Images for additional controls and timepoints for Fig 7. Vero cells were infected or mock infected with rRSVflag(2)L at a MOI of 3. Cells were transfected with 200 ng of M2-2-Myc or M2-1-HA mRNA at 3 hpi. A-B) Cells were fixed at 6 (A) or 8 (B) hours post infection. FISH with PMTRIPs was performed for RSV genome, RSV NS1 mRNA, without an epitope tag, a scrambled control, or without targeting oligos. PLA (white) was performed between RSV genome/ NS1 mRNA and L. Cells were stained for M2-2 via Myc (green) or M2-1 via HA (green), and F (red). Duplicates were performed, but representative extended focus images from one experiment are shown. Scale bar is 10 μm. C) Additional quantification for Fig 7, with mean and 95% confidence intervals in grey. Statistics were two-way ANOVAs with Tukey’s multiple comparison tests where n = 30 cells and * p < 0.05, ** p < 0.01, *** p < 0.001, and **** p < 0.0001. Quantification of PLA volume (right) is normalized by volume of RSV F per cell. (TIF) [file ppat.1008987.s009.tif]
