# Supplementary material for: Polymerase-tagged respiratory syncytial virus reveals a dynamic rearrangement of the ribonucleocapsid complex during infection
Source: PLoS Pathog. 2020 Oct 8;16(10):e1008987. doi: 10.1371/journal.ppat.1008987 (PMC7575074; doi:10.1371/journal.ppat.1008987)
Supplement: S1 Table — (PDF) [file ppat.1008987.s010.pdf]

Figure 3B	
**Graph Title**	N-L PLA
Comparison	P Value
1 hpi vs. 6 hpi	<0.0001
1 hpi vs. 8 hpi	>0.9999
1 hpi vs. 12 hpi	>0.9999
1 hpi vs. 18 hpi	>0.9999
1 hpi vs. 24 hpi	0.0063
6 hpi vs. 8 hpi	<0.0001
6 hpi vs. 12 hpi	<0.0001
6 hpi vs. 18 hpi	<0.0001
6 hpi vs. 24 hpi	<0.0001
8 hpi vs. 12 hpi	>0.9999
8 hpi vs. 18 hpi	>0.9999
8 hpi vs. 24 hpi	0.0316
12 hpi vs. 18 hpi	>0.9999
12 hpi vs. 24 hpi	0.0154
18 hpi vs. 24 hpi	0.0267
**Graph Title**	P-L PLA
Comparison	P Value
1 hpi vs. 6 hpi	>0.9999
1 hpi vs. 8 hpi	<0.0001
1 hpi vs. 12 hpi	0.9262
1 hpi vs. 18 hpi	<0.0001
1 hpi vs. 24 hpi	<0.0001
6 hpi vs. 8 hpi	<0.0001
6 hpi vs. 12 hpi	0.4174
6 hpi vs. 18 hpi	<0.0001
6 hpi vs. 24 hpi	<0.0001
8 hpi vs. 12 hpi	<0.0001
8 hpi vs. 18 hpi	>0.9999
8 hpi vs. 24 hpi	>0.9999
12 hpi vs. 18 hpi	<0.0001
12 hpi vs. 24 hpi	0.0006
18 hpi vs. 24 hpi	>0.9999
**Graph Title**	M2-1-L PLA
Comparison	P Value
1 hpi vs. 6 hpi	0.2487
1 hpi vs. 8 hpi	<0.0001
1 hpi vs. 12 hpi	0.0647
1 hpi vs. 18 hpi	<0.0001
1 hpi vs. 24 hpi	<0.0001
6 hpi vs. 8 hpi	<0.0001
6 hpi vs. 12 hpi	>0.9999
6 hpi vs. 18 hpi	0.0002
6 hpi vs. 24 hpi	<0.0001
8 hpi vs. 12 hpi	<0.0001
8 hpi vs. 18 hpi	>0.9999
8 hpi vs. 24 hpi	>0.9999
12 hpi vs. 18 hpi	0.0013
12 hpi vs. 24 hpi	<0.0001

18 hpi vs. 24 hpi	>0.9999

Figure 3C	
**Graph Title**	N-L Centroid Distance
Comparison	P Value
6 hpi vs 8 hpi	0.0026

Figure 4B	
**Graph Title**	RSV genome-L PLA (# of Puncta)
Comparison	P Value
6 hpi :RSV genome-L vs. 6 hpi :No Primary Control	0.0018
6 hpi :RSV genome-L vs. 6 hpi :No V5 Control	0.0023
6 hpi :RSV genome-L vs. 6 hpi :Scrambled Control	0.0059
6 hpi :RSV genome-L vs. 6 hpi :Biotin Control	0.0143
6 hpi :RSV genome-L vs. 6 hpi :No FISH Control	<0.0001
6 hpi :RSV genome-L vs. 6 hpi :Mock Control	0.0189
6 hpi :RSV genome-L vs. 8 hpi :RSV genome-L	>0.9999
6 hpi :RSV genome-L vs. 8 hpi :No Primary Control	0.0017
6 hpi :RSV genome-L vs. 8 hpi :No V5 Control	0.0059
6 hpi :RSV genome-L vs. 8 hpi :Scrambled Control	0.0004
6 hpi :RSV genome-L vs. 8 hpi :Biotin Control	0.0322
6 hpi :RSV genome-L vs. 8 hpi :No FISH Control	0.0032
6 hpi :RSV genome-L vs. 8 hpi :Mock Control	0.0004
6 hpi :No Primary Control vs. 6 hpi :No V5 Control	>0.9999
6 hpi :No Primary Control vs. 6 hpi :Scrambled Control	>0.9999
6 hpi :No Primary Control vs. 6 hpi :Biotin Control	>0.9999
6 hpi :No Primary Control vs. 6 hpi :No FISH Control	>0.9999
6 hpi :No Primary Control vs. 6 hpi :Mock Control	>0.9999
6 hpi :No Primary Control vs. 8 hpi :RSV genome-L	0.0047
6 hpi :No Primary Control vs. 8 hpi :No Primary Control	>0.9999
6 hpi :No Primary Control vs. 8 hpi :No V5 Control	>0.9999
6 hpi :No Primary Control vs. 8 hpi :Scrambled Control	>0.9999
6 hpi :No Primary Control vs. 8 hpi :Biotin Control	>0.9999
6 hpi :No Primary Control vs. 8 hpi :No FISH Control	>0.9999
6 hpi :No Primary Control vs. 8 hpi :Mock Control	>0.9999
6 hpi :No V5 Control vs. 6 hpi :Scrambled Control	>0.9999
6 hpi :No V5 Control vs. 6 hpi :Biotin Control	>0.9999
6 hpi :No V5 Control vs. 6 hpi :No FISH Control	>0.9999
6 hpi :No V5 Control vs. 6 hpi :Mock Control	>0.9999
6 hpi :No V5 Control vs. 8 hpi :RSV genome-L	0.0059
6 hpi :No V5 Control vs. 8 hpi :No Primary Control	>0.9999
6 hpi :No V5 Control vs. 8 hpi :No V5 Control	>0.9999
6 hpi :No V5 Control vs. 8 hpi :Scrambled Control	>0.9999
6 hpi :No V5 Control vs. 8 hpi :Biotin Control	>0.9999
6 hpi :No V5 Control vs. 8 hpi :No FISH Control	>0.9999
6 hpi :No V5 Control vs. 8 hpi :Mock Control	>0.9999
6 hpi :Scrambled Control vs. 6 hpi :Biotin Control	>0.9999
6 hpi :Scrambled Control vs. 6 hpi :No FISH Control	>0.9999
6 hpi :Scrambled Control vs. 6 hpi :Mock Control	>0.9999
6 hpi :Scrambled Control vs. 8 hpi :RSV genome-L	0.0143
6 hpi :Scrambled Control vs. 8 hpi :No Primary Control	>0.9999
6 hpi :Scrambled Control vs. 8 hpi :No V5 Control	>0.9999

6 hpi :Scrambled Control vs. 8 hpi :Scrambled Control	>0.9999
6 hpi :Scrambled Control vs. 8 hpi :Biotin Control	>0.9999
6 hpi :Scrambled Control vs. 8 hpi :No FISH Control	>0.9999
6 hpi :Scrambled Control vs. 8 hpi :Mock Control	>0.9999
6 hpi :Biotin Control vs. 6 hpi :Mock Control	>0.9999
6 hpi :Biotin Control vs. 8 hpi :RSV genome-L	0.0322
6 hpi :Biotin Control vs. 8 hpi :No Primary Control	>0.9999
6 hpi :Biotin Control vs. 8 hpi :No V5 Control	>0.9999
6 hpi :Biotin Control vs. 8 hpi :Scrambled Control	>0.9999
6 hpi :Biotin Control vs. 8 hpi :Biotin Control	>0.9999
6 hpi :Biotin Control vs. 8 hpi :No FISH Control	>0.9999
6 hpi :Biotin Control vs. 8 hpi :Mock Control	>0.9999
6 hpi :No FISH Control vs. 6 hpi :Mock Control	0.9992
6 hpi :No FISH Control vs. 8 hpi :RSV genome-L	<0.0001
6 hpi :No FISH Control vs. 8 hpi :No Primary Control	>0.9999
6 hpi :No FISH Control vs. 8 hpi :No V5 Control	>0.9999
6 hpi :No FISH Control vs. 8 hpi :Scrambled Control	>0.9999
6 hpi :No FISH Control vs. 8 hpi :Biotin Control	0.9966
6 hpi :No FISH Control vs. 8 hpi :No FISH Control	>0.9999
6 hpi :No FISH Control vs. 8 hpi :Mock Control	>0.9999
6 hpi :Mock Control vs. 8 hpi :RSV genome-L	0.0416
6 hpi :Mock Control vs. 8 hpi :No Primary Control	>0.9999
6 hpi :Mock Control vs. 8 hpi :No V5 Control	>0.9999
6 hpi :Mock Control vs. 8 hpi :Scrambled Control	>0.9999
6 hpi :Mock Control vs. 8 hpi :Biotin Control	>0.9999
6 hpi :Mock Control vs. 8 hpi :No FISH Control	>0.9999
6 hpi :Mock Control vs. 8 hpi :Mock Control	>0.9999
8 hpi :RSV genome-L vs. 8 hpi :No Primary Control	0.0044
8 hpi :RSV genome-L vs. 8 hpi :No V5 Control	0.0143
8 hpi :RSV genome-L vs. 8 hpi :Scrambled Control	0.0012
8 hpi :RSV genome-L vs. 8 hpi :Biotin Control	0.0676
8 hpi :RSV genome-L vs. 8 hpi :No FISH Control	0.008
8 hpi :RSV genome-L vs. 8 hpi :Mock Control	0.0012
8 hpi :No Primary Control vs. 8 hpi :No V5 Control	>0.9999
8 hpi :No Primary Control vs. 8 hpi :Scrambled Control	>0.9999
8 hpi :No Primary Control vs. 8 hpi :Biotin Control	>0.9999
8 hpi :No Primary Control vs. 8 hpi :No FISH Control	>0.9999
8 hpi :No Primary Control vs. 8 hpi :Mock Control	>0.9999
8 hpi :No V5 Control vs. 8 hpi :Biotin Control	>0.9999
8 hpi :No V5 Control vs. 8 hpi :No FISH Control	>0.9999
8 hpi :No V5 Control vs. 8 hpi :Mock Control	>0.9999
8 hpi :Scrambled Control vs. 8 hpi :Biotin Control	>0.9999
8 hpi :Scrambled Control vs. 8 hpi :No FISH Control	>0.9999
8 hpi :Scrambled Control vs. 8 hpi :Mock Control	>0.9999
8 hpi :Biotin Control vs. 8 hpi :No FISH Control	>0.9999
8 hpi :Biotin Control vs. 8 hpi :Mock Control	>0.9999
8 hpi :No FISH Control vs. 8 hpi :Mock Control	>0.9999
**Graph Title**	RSV genome-L PLA (Normalized PLA)
Comparison	P Value
6 hpi	
RSV genome-L vs. No Primary Control	0.0003
RSV genome-L vs. No V5 Control	<0.0001
RSV genome-L vs. Scrambled Control	<0.0001

RSV genome-L vs. Biotin Control	<0.0001
RSV genome-L vs. No FISH Control	<0.0001
No Primary Control vs. No V5 Control	0.895
No Primary Control vs. Scrambled Control	0.9837
No Primary Control vs. Biotin Control	0.9998
No Primary Control vs. No FISH Control	0.8005
No V5 Control vs. Scrambled Control	0.9989
No V5 Control vs. Biotin Control	0.9676
No V5 Control vs. No FISH Control	>0.9999
Scrambled Control vs. Biotin Control	0.9985
Scrambled Control vs. No FISH Control	0.9912
Biotin Control vs. No FISH Control	0.9148
8 hpi	<0.0001
RSV genome-L vs. No Primary Control	<0.0001
RSV genome-L vs. No V5 Control	<0.0001
RSV genome-L vs. Scrambled Control	0.0006
RSV genome-L vs. Biotin Control	<0.0001
RSV genome-L vs. No FISH Control	0.9994
No Primary Control vs. No V5 Control	0.9997
No Primary Control vs. Scrambled Control	0.9945
No Primary Control vs. Biotin Control	>0.9999
No Primary Control vs. No FISH Control	>0.9999
No V5 Control vs. Scrambled Control	0.9518
No V5 Control vs. Biotin Control	0.9977
No V5 Control vs. No FISH Control	0.9605
Scrambled Control vs. Biotin Control	0.9985
Scrambled Control vs. No FISH Control	0.9982
Biotin Control vs. No FISH Control	
**Graph Title**	NS1 mRNA-L PLA (# of Puncta)
Comparison	P Value
6 hpi :NS1 mRNA-L vs. 6 hpi :No Primary Control	<0.0001
6 hpi :NS1 mRNA-L vs. 6 hpi :No V5 Control	<0.0001
6 hpi :NS1 mRNA-L vs. 6 hpi :Scrambled Control	<0.0001
6 hpi :NS1 mRNA-L vs. 6 hpi :Biotin Control	<0.0001
6 hpi :NS1 mRNA-L vs. 6 hpi :No FISH Control	<0.0001
6 hpi :NS1 mRNA-L vs. 6 hpi :Mock Control	<0.0001
6 hpi :NS1 mRNA-L vs. 8 hpi :NS1 mRNA-L	<0.0001
6 hpi :NS1 mRNA-L vs. 8 hpi :No Primary Control	<0.0001
6 hpi :NS1 mRNA-L vs. 8 hpi :No V5 Control	<0.0001
6 hpi :NS1 mRNA-L vs. 8 hpi :Scrambled Control	<0.0001
6 hpi :NS1 mRNA-L vs. 8 hpi :Biotin Control	<0.0001
6 hpi :NS1 mRNA-L vs. 8 hpi :No FISH Control	<0.0001
6 hpi :NS1 mRNA-L vs. 8 hpi :Mock Control	<0.0001
6 hpi :No Primary Control vs. 6 hpi :No V5 Control	>0.9999
6 hpi :No Primary Control vs. 6 hpi :Scrambled Control	>0.9999
6 hpi :No Primary Control vs. 6 hpi :Biotin Control	>0.9999
6 hpi :No Primary Control vs. 6 hpi :No FISH Control	>0.9999
6 hpi :No Primary Control vs. 6 hpi :Mock Control	0.1407
6 hpi :No Primary Control vs. 8 hpi :NS1 mRNA-L	<0.0001
6 hpi :No Primary Control vs. 8 hpi :No Primary Control	>0.9999
6 hpi :No Primary Control vs. 8 hpi :No V5 Control	>0.9999
6 hpi :No Primary Control vs. 8 hpi :Scrambled Control	>0.9999
6 hpi :No Primary Control vs. 8 hpi :Biotin Control	0.9996

6 hpi :No Primary Control vs. 8 hpi :No FISH Control	>0.9999
6 hpi :No Primary Control vs. 8 hpi :Mock Control	0.0011
6 hpi :No V5 Control vs. 6 hpi :Scrambled Control	>0.9999
6 hpi :No V5 Control vs. 6 hpi :Biotin Control	>0.9999
6 hpi :No V5 Control vs. 6 hpi :No FISH Control	0.9993
6 hpi :No V5 Control vs. 6 hpi :Mock Control	0.46
6 hpi :No V5 Control vs. 8 hpi :NS1 mRNA-L	<0.0001
6 hpi :No V5 Control vs. 8 hpi :No Primary Control	>0.9999
6 hpi :No V5 Control vs. 8 hpi :No V5 Control	>0.9999
6 hpi :No V5 Control vs. 8 hpi :Scrambled Control	>0.9999
6 hpi :No V5 Control vs. 8 hpi :Biotin Control	>0.9999
6 hpi :No V5 Control vs. 8 hpi :No FISH Control	>0.9999
6 hpi :No V5 Control vs. 8 hpi :Mock Control	0.0099
6 hpi :Scrambled Control vs. 6 hpi :Biotin Control	>0.9999
6 hpi :Scrambled Control vs. 6 hpi :No FISH Control	>0.9999
6 hpi :Scrambled Control vs. 6 hpi :Mock Control	0.1679
6 hpi :Scrambled Control vs. 8 hpi :NS1 mRNA-L	<0.0001
6 hpi :Scrambled Control vs. 8 hpi :No Primary Control	>0.9999
6 hpi :Scrambled Control vs. 8 hpi :No V5 Control	>0.9999
6 hpi :Scrambled Control vs. 8 hpi :Scrambled Control	>0.9999
6 hpi :Scrambled Control vs. 8 hpi :Biotin Control	0.9998
6 hpi :Scrambled Control vs. 8 hpi :No FISH Control	>0.9999
6 hpi :Scrambled Control vs. 8 hpi :Mock Control	0.0014
6 hpi :Biotin Control vs. 6 hpi :No FISH Control	>0.9999
6 hpi :Biotin Control vs. 6 hpi :Mock Control	0.2722
6 hpi :Biotin Control vs. 8 hpi :NS1 mRNA-L	<0.0001
6 hpi :Biotin Control vs. 8 hpi :No Primary Control	>0.9999
6 hpi :Biotin Control vs. 8 hpi :No V5 Control	>0.9999
6 hpi :Biotin Control vs. 8 hpi :Scrambled Control	>0.9999
6 hpi :Biotin Control vs. 8 hpi :Biotin Control	>0.9999
6 hpi :Biotin Control vs. 8 hpi :No FISH Control	>0.9999
6 hpi :Biotin Control vs. 8 hpi :Mock Control	0.0034
6 hpi :No FISH Control vs. 6 hpi :Mock Control	0.0014
6 hpi :No FISH Control vs. 8 hpi :NS1 mRNA-L	<0.0001
6 hpi :No FISH Control vs. 8 hpi :No Primary Control	>0.9999
6 hpi :No FISH Control vs. 8 hpi :No V5 Control	0.9998
6 hpi :No FISH Control vs. 8 hpi :Scrambled Control	>0.9999
6 hpi :No FISH Control vs. 8 hpi :Biotin Control	0.5664
6 hpi :No FISH Control vs. 8 hpi :No FISH Control	>0.9999
6 hpi :No FISH Control vs. 8 hpi :Mock Control	<0.0001
6 hpi :Mock Control vs. 8 hpi :NS1 mRNA-L	0.2336
6 hpi :Mock Control vs. 8 hpi :No Primary Control	0.2722
6 hpi :Mock Control vs. 8 hpi :No V5 Control	0.3602
6 hpi :Mock Control vs. 8 hpi :Scrambled Control	0.0334
6 hpi :Mock Control vs. 8 hpi :Biotin Control	0.9973
6 hpi :Mock Control vs. 8 hpi :No FISH Control	0.117
6 hpi :Mock Control vs. 8 hpi :Mock Control	>0.9999
8 hpi :NS1 mRNA-L vs. 8 hpi :No Primary Control	<0.0001
8 hpi :NS1 mRNA-L vs. 8 hpi :No V5 Control	<0.0001
8 hpi :NS1 mRNA-L vs. 8 hpi :Scrambled Control	<0.0001
8 hpi :NS1 mRNA-L vs. 8 hpi :Biotin Control	0.0002
8 hpi :NS1 mRNA-L vs. 8 hpi :No FISH Control	<0.0001
8 hpi :NS1 mRNA-L vs. 8 hpi :Mock Control	0.9866
8 hpi :No Primary Control vs. 8 hpi :No V5 Control	>0.9999

8 hpi :No Primary Control vs. 8 hpi :Scrambled Control	>0.9999
8 hpi :No Primary Control vs. 8 hpi :Biotin Control	>0.9999
8 hpi :No Primary Control vs. 8 hpi :No FISH Control	>0.9999
8 hpi :No Primary Control vs. 8 hpi :Mock Control	0.0034
8 hpi :No V5 Control vs. 8 hpi :Scrambled Control	>0.9999
8 hpi :No V5 Control vs. 8 hpi :Biotin Control	>0.9999
8 hpi :No V5 Control vs. 8 hpi :No FISH Control	>0.9999
8 hpi :No V5 Control vs. 8 hpi :Mock Control	0.0058
8 hpi :Scrambled Control vs. 8 hpi :Biotin Control	0.979
8 hpi :Scrambled Control vs. 8 hpi :No FISH Control	>0.9999
8 hpi :Scrambled Control vs. 8 hpi :Mock Control	0.0001
8 hpi :Biotin Control vs. 8 hpi :No FISH Control	0.9993
8 hpi :Biotin Control vs. 8 hpi :Mock Control	0.3602
8 hpi :No FISH Control vs. 8 hpi :Mock Control	0.0008
**Graph Title**	NS1 mRNA-L PLA (Normalized PLA)
Comparison	P Value
6 hpi	
NS1 mRNA-L vs. No Primary Control	<0.0001
NS1 mRNA-L vs. No V5 Control	<0.0001
NS1 mRNA-L vs. Scrambled Control	<0.0001
NS1 mRNA-L vs. Biotin Control	<0.0001
NS1 mRNA-L vs. No FISH Control	<0.0001
No Primary Control vs. No V5 Control	0.7602
No Primary Control vs. Scrambled Control	>0.9999
No Primary Control vs. Biotin Control	0.9952
No Primary Control vs. No FISH Control	0.9557
No V5 Control vs. Scrambled Control	0.7358
No V5 Control vs. Biotin Control	0.9638
No V5 Control vs. No FISH Control	0.2367
Scrambled Control vs. Biotin Control	0.9932
Scrambled Control vs. No FISH Control	0.9641
Biotin Control vs. No FISH Control	0.7368
8 hpi	0.2007
NS1 mRNA-L vs. No Primary Control	0.5055
NS1 mRNA-L vs. No V5 Control	0.0006
NS1 mRNA-L vs. Scrambled Control	0.0362
NS1 mRNA-L vs. Biotin Control	0.0054
NS1 mRNA-L vs. No FISH Control	0.9942
No Primary Control vs. No V5 Control	0.447
No Primary Control vs. Scrambled Control	0.9839
No Primary Control vs. Biotin Control	0.8028
No Primary Control vs. No FISH Control	0.1658
No V5 Control vs. Scrambled Control	0.823
No V5 Control vs. Biotin Control	0.4574
No V5 Control vs. No FISH Control	0.8612
Scrambled Control vs. Biotin Control	0.9934
Scrambled Control vs. No FISH Control	0.992
Biotin Control vs. No FISH Control	

**Figure 6B**	
**Graph Title**	N-L PLA (# of Puncta)
Comparison	P Value
Row 1:N-L vs. Row 1:No Primary Control	>0.9999

Row 1:N-L vs. Row 1:Mock Control	0.0057
Row 1:N-L vs. Row 2:N-L	0.315
Row 1:N-L vs. Row 2:No Primary Control	0.0019
Row 1:N-L vs. Row 2:Mock Control	0.0225
Row 1:N-L vs. Row 3:N-L	<0.0001
Row 1:N-L vs. Row 3:No Primary Control	0.3491
Row 1:N-L vs. Row 3:Mock Control	0.385
Row 1:N-L vs. Row 4:N-L	0.0865
Row 1:N-L vs. Row 4:No Primary Control	0.6207
Row 1:N-L vs. Row 4:Mock Control	0.0002
Row 1:No Primary Control vs. Row 1:Mock Control	0.0187
Row 1:No Primary Control vs. Row 2:N-L	0.5405
Row 1:No Primary Control vs. Row 2:No Primary Control	0.007
Row 1:No Primary Control vs. Row 2:Mock Control	0.0633
Row 1:No Primary Control vs. Row 3:N-L	<0.0001
Row 1:No Primary Control vs. Row 3:No Primary Control	0.5807
Row 1:No Primary Control vs. Row 3:Mock Control	0.6207
Row 1:No Primary Control vs. Row 4:N-L	0.1987
Row 1:No Primary Control vs. Row 4:No Primary Control	0.8325
Row 1:No Primary Control vs. Row 4:Mock Control	0.0008
Row 1:Mock Control vs. Row 2:N-L	0.966
Row 1:Mock Control vs. Row 2:No Primary Control	>0.9999
Row 1:Mock Control vs. Row 2:Mock Control	>0.9999
Row 1:Mock Control vs. Row 3:N-L	<0.0001
Row 1:Mock Control vs. Row 3:No Primary Control	0.9549
Row 1:Mock Control vs. Row 3:Mock Control	0.9414
Row 1:Mock Control vs. Row 4:N-L	0.9996
Row 1:Mock Control vs. Row 4:No Primary Control	0.8023
Row 1:Mock Control vs. Row 4:Mock Control	0.9996
Row 2:N-L vs. Row 2:No Primary Control	0.8846
Row 2:N-L vs. Row 2:Mock Control	0.9977
Row 2:N-L vs. Row 3:N-L	<0.0001
Row 2:N-L vs. Row 3:No Primary Control	>0.9999
Row 2:N-L vs. Row 3:Mock Control	>0.9999
Row 2:N-L vs. Row 4:N-L	>0.9999
Row 2:N-L vs. Row 4:No Primary Control	>0.9999
Row 2:N-L vs. Row 4:Mock Control	0.5405
Row 2:No Primary Control vs. Row 2:Mock Control	>0.9999
Row 2:No Primary Control vs. Row 3:N-L	<0.0001
Row 2:No Primary Control vs. Row 3:No Primary Control	0.8599
Row 2:No Primary Control vs. Row 3:Mock Control	0.8325
Row 2:No Primary Control vs. Row 4:N-L	0.9942
Row 2:No Primary Control vs. Row 4:No Primary Control	0.6207
Row 2:No Primary Control vs. Row 4:Mock Control	>0.9999
Row 2:Mock Control vs. Row 3:N-L	<0.0001
Row 2:Mock Control vs. Row 3:No Primary Control	0.9963
Row 2:Mock Control vs. Row 3:Mock Control	0.9942
Row 2:Mock Control vs. Row 4:N-L	>0.9999
Row 2:Mock Control vs. Row 4:No Primary Control	0.9549
Row 2:Mock Control vs. Row 4:Mock Control	0.9873
Row 3:N-L vs. Row 3:No Primary Control	<0.0001
Row 3:N-L vs. Row 3:Mock Control	<0.0001
Row 3:N-L vs. Row 4:N-L	<0.0001
Row 3:N-L vs. Row 4:No Primary Control	<0.0001

Row 3:N-L vs. Row 4:Mock Control	0.0001
Row 3:No Primary Control vs. Row 3:Mock Control	>0.9999
Row 3:No Primary Control vs. Row 4:N-L	>0.9999
Row 3:No Primary Control vs. Row 4:No Primary Control	>0.9999
Row 3:No Primary Control vs. Row 4:Mock Control	0.5004
Row 3:Mock Control vs. Row 4:N-L	>0.9999
Row 3:Mock Control vs. Row 4:No Primary Control	>0.9999
Row 3:Mock Control vs. Row 4:Mock Control	0.4609
Row 4:N-L vs. Row 4:No Primary Control	0.9977
Row 4:N-L vs. Row 4:Mock Control	0.8846
Row 4:No Primary Control vs. Row 4:Mock Control	0.2527
**Graph Title**	N-L PLA (Normalized PLA)
Comparison	P Value
Row 1:N-L vs. Row 1:No Primary Control	0.6865
Row 1:N-L vs. Row 2:N-L	0.0744
Row 1:N-L vs. Row 2:No Primary Control	0.0281
Row 1:N-L vs. Row 3:N-L	0.0003
Row 1:N-L vs. Row 3:No Primary Control	0.7546
Row 1:N-L vs. Row 4:N-L	0.9679
Row 1:N-L vs. Row 4:No Primary Control	>0.9999
Row 1:No Primary Control vs. Row 2:N-L	0.0002
Row 1:No Primary Control vs. Row 2:No Primary Control	<0.0001
Row 1:No Primary Control vs. Row 3:N-L	<0.0001
Row 1:No Primary Control vs. Row 3:No Primary Control	>0.9999
Row 1:No Primary Control vs. Row 4:N-L	0.1212
Row 1:No Primary Control vs. Row 4:No Primary Control	0.6052
Row 2:N-L vs. Row 2:No Primary Control	>0.9999
Row 2:N-L vs. Row 3:N-L	0.756
Row 2:N-L vs. Row 3:No Primary Control	0.0003
Row 2:N-L vs. Row 4:N-L	0.5693
Row 2:N-L vs. Row 4:No Primary Control	0.1025
Row 2:No Primary Control vs. Row 3:N-L	0.928
Row 2:No Primary Control vs. Row 3:No Primary Control	<0.0001
Row 2:No Primary Control vs. Row 4:N-L	0.341
Row 2:No Primary Control vs. Row 4:No Primary Control	0.0406
Row 3:N-L vs. Row 3:No Primary Control	<0.0001
Row 3:N-L vs. Row 4:N-L	0.0144
Row 3:N-L vs. Row 4:No Primary Control	0.0005
Row 3:No Primary Control vs. Row 4:N-L	0.1502
Row 3:No Primary Control vs. Row 4:No Primary Control	0.6768
Row 4:N-L vs. Row 4:No Primary Control	0.9844
**Figure 6C**	
**Graph Title**	N-L Centroid Distance
Comparison	P Value
6 hpi vs 8 hpi	<0.0001

**Figure 7B**	
**Graph Title**	RSV genome-L PLA (# of Puncta)
Comparison	P Value
M2-2-Myc mRNA	
RSVg-L vs. No Primary Control	<0.0001
RSVg-L vs. Mock Infected Control	0.7904

No Primary Control vs. Mock Infected Control	<0.0001
M2-1-HA mRNA	
RSVg-L vs. No Primary Control	<0.0001
RSVg-L vs. Mock Infected Control	<0.0001
No Primary Control vs. Mock Infected Control	<0.0001
**Graph Title**	RSV genome-L PLA (Normalized PLA)
Comparison	P Value
M2-2 Myc RNA RSVg-L vs. No Primary Control	0.1579
M2-1 HA RNA RSVg-L vs. No Primary Control	<0.0001
**Graph Title**	NS1 mRNA-L PLA (# of Puncta)
Comparison	P Value
M2-2-Myc mRNA	
NS1-L vs. No Primary Control	<0.0001
NS1-L vs. Mock Infected Control	0.8752
No Primary Control vs. Mock Infected Control	<0.0001
M2-1-HA mRNA	
NS1-L vs. No Primary Control	<0.0001
NS1-L vs. Mock Infected Control	<0.0001
No Primary Control vs. Mock Infected Control	0.006
**Graph Title**	NS1 mRNA-L PLA (Normalized PLA)
Comparison	P Value
M2-2 Myc NS1-L vs. No Primary Control	0.758
M2-1 HA NS1-L vs. No Primary Control	<0.0001

**Figure S3B**	
**Graph Title**	N-L PLA
Comparison	P Value
1 hpi	
RSV-Fl(2)-L vs. Mock Infected	0.9993
RSV-Fl(2)-L vs. No Primary Control	0.7023
Mock Infected vs. No Primary Control	0.6806
6 hpi	
RSV-Fl(2)-L vs. Mock Infected	0.025
RSV-Fl(2)-L vs. No Primary Control	0.0032
Mock Infected vs. No Primary Control	0.7856
8 hpi	
RSV-Fl(2)-L vs. Mock Infected	0.516
RSV-Fl(2)-L vs. No Primary Control	0.4486
Mock Infected vs. No Primary Control	0.9921
12 hpi	
RSV-Fl(2)-L vs. Mock Infected	<0.0001
RSV-Fl(2)-L vs. No Primary Control	<0.0001
Mock Infected vs. No Primary Control	0.1555
18 hpi	
RSV-Fl(2)-L vs. Mock Infected	<0.0001
RSV-Fl(2)-L vs. No Primary Control	<0.0001
Mock Infected vs. No Primary Control	0.8688
24 hpi	
RSV-Fl(2)-L vs. Mock Infected	<0.0001
RSV-Fl(2)-L vs. No Primary Control	<0.0001
Mock Infected vs. No Primary Control	0.5926

**Graph Title**	N-L PLA # of Puncta
Comparison	P Value
RSV-FI(2)-L	
1 hpi vs. 6 hpi	0.0012
1 hpi vs. 8 hpi	0.9966
1 hpi vs. 12 hpi	<0.0001
1 hpi vs. 18 hpi	<0.0001
1 hpi vs. 24 hpi	<0.0001
6 hpi vs. 8 hpi	0.0075
6 hpi vs. 12 hpi	0.5506
6 hpi vs. 18 hpi	0.322
6 hpi vs. 24 hpi	<0.0001
8 hpi vs. 12 hpi	<0.0001
8 hpi vs. 18 hpi	<0.0001
8 hpi vs. 24 hpi	<0.0001
12 hpi vs. 18 hpi	0.9991
12 hpi vs. 24 hpi	<0.0001
18 hpi vs. 24 hpi	<0.0001
Mock Infected	
1 hpi vs. 6 hpi	0.7413
1 hpi vs. 8 hpi	0.9926
1 hpi vs. 12 hpi	0.9976
1 hpi vs. 18 hpi	0.9966
1 hpi vs. 24 hpi	0.9998
6 hpi vs. 8 hpi	0.3721
6 hpi vs. 12 hpi	0.4477
6 hpi vs. 18 hpi	0.9492
6 hpi vs. 24 hpi	0.8751
8 hpi vs. 12 hpi	>0.9999
8 hpi vs. 18 hpi	0.8959
8 hpi vs. 24 hpi	0.9606
12 hpi vs. 18 hpi	0.9357
12 hpi vs. 24 hpi	0.9802
18 hpi vs. 24 hpi	>0.9999
No Primary Control	
1 hpi vs. 6 hpi	>0.9999
1 hpi vs. 8 hpi	0.6518
1 hpi vs. 12 hpi	0.9936
1 hpi vs. 18 hpi	0.957
1 hpi vs. 24 hpi	0.9987
6 hpi vs. 8 hpi	0.7279
6 hpi vs. 12 hpi	0.9842
6 hpi vs. 18 hpi	0.978
6 hpi vs. 24 hpi	0.9953
8 hpi vs. 12 hpi	0.3035
8 hpi vs. 18 hpi	0.9848
8 hpi vs. 24 hpi	0.3929
12 hpi vs. 18 hpi	0.7202
12 hpi vs. 24 hpi	>0.9999
18 hpi vs. 24 hpi	0.8095
**Graph Title**	P-L PLA
Comparison	P Value

1 hpi	
RSV-Fl(2)-L vs. Mock Infected	0.0704
RSV-Fl(2)-L vs. No Primary Control	0.0959
Mock Infected vs. No Primary Control	0.9901
6 hpi	
RSV-Fl(2)-L vs. Mock Infected	0.0151
RSV-Fl(2)-L vs. No Primary Control	0.0157
Mock Infected vs. No Primary Control	0.9999
8 hpi	
RSV-Fl(2)-L vs. Mock Infected	0.9082
RSV-Fl(2)-L vs. No Primary Control	0.9383
Mock Infected vs. No Primary Control	0.9969
12 hpi	
RSV-Fl(2)-L vs. Mock Infected	0.0005
RSV-Fl(2)-L vs. No Primary Control	0.0002
Mock Infected vs. No Primary Control	0.952
18 hpi	
RSV-Fl(2)-L vs. Mock Infected	<0.0001
RSV-Fl(2)-L vs. No Primary Control	<0.0001
Mock Infected vs. No Primary Control	0.952
24 hpi	
RSV-Fl(2)-L vs. Mock Infected	<0.0001
RSV-Fl(2)-L vs. No Primary Control	<0.0001
Mock Infected vs. No Primary Control	0.7268

**Graph Title**	P-L PLA # of Puncta
Comparison	P Value
RSV-Fl(2)-L	
1 hpi vs. 6 hpi	0.9991
1 hpi vs. 8 hpi	0.0292
1 hpi vs. 12 hpi	0.1323
1 hpi vs. 18 hpi	<0.0001
1 hpi vs. 24 hpi	<0.0001
6 hpi vs. 8 hpi	0.0088
6 hpi vs. 12 hpi	0.286
6 hpi vs. 18 hpi	<0.0001
6 hpi vs. 24 hpi	<0.0001
8 hpi vs. 12 hpi	<0.0001
8 hpi vs. 18 hpi	<0.0001
8 hpi vs. 24 hpi	<0.0001
12 hpi vs. 18 hpi	0.0995
12 hpi vs. 24 hpi	<0.0001
18 hpi vs. 24 hpi	<0.0001
Mock Infected	
1 hpi vs. 6 hpi	0.9998
1 hpi vs. 8 hpi	0.8095
1 hpi vs. 12 hpi	0.9437
1 hpi vs. 18 hpi	0.9474
1 hpi vs. 24 hpi	0.9998
6 hpi vs. 8 hpi	0.9342
6 hpi vs. 12 hpi	0.855
6 hpi vs. 18 hpi	0.9912
6 hpi vs. 24 hpi	>0.9999
8 hpi vs. 12 hpi	0.256

8 hpi vs. 18 hpi	0.9992
8 hpi vs. 24 hpi	0.9271
12 hpi vs. 18 hpi	0.4622
12 hpi vs. 24 hpi	0.8394
18 hpi vs. 24 hpi	0.9901
No Primary Control	
1 hpi vs. 6 hpi	0.9984
1 hpi vs. 8 hpi	0.7837
1 hpi vs. 12 hpi	0.9969
1 hpi vs. 18 hpi	0.978
1 hpi vs. 24 hpi	0.9994
6 hpi vs. 8 hpi	0.9595
6 hpi vs. 12 hpi	0.9517
6 hpi vs. 18 hpi	0.9997
6 hpi vs. 24 hpi	0.9757
8 hpi vs. 12 hpi	0.4821
8 hpi vs. 18 hpi	0.9925
8 hpi vs. 24 hpi	0.5704
12 hpi vs. 18 hpi	0.8322
12 hpi vs. 24 hpi	>0.9999
18 hpi vs. 24 hpi	0.8912

**Graph Title**	M2-1-L PLA
Comparison	P Value
1 hpi	
RSV-Fl(2)-L vs. Mock Infected	0.0329
RSV-Fl(2)-L vs. No Primary Control	0.0722
Mock Infected vs. No Primary Control	0.9468
6 hpi	
RSV-Fl(2)-L vs. Mock Infected	0.4135
RSV-Fl(2)-L vs. No Primary Control	0.064
Mock Infected vs. No Primary Control	0.6408
8 hpi	
RSV-Fl(2)-L vs. Mock Infected	0.208
RSV-Fl(2)-L vs. No Primary Control	0.5703
Mock Infected vs. No Primary Control	0.7811
12 hpi	
RSV-Fl(2)-L vs. Mock Infected	<0.0001
RSV-Fl(2)-L vs. No Primary Control	<0.0001
Mock Infected vs. No Primary Control	0.8591
18 hpi	
RSV-Fl(2)-L vs. Mock Infected	<0.0001
RSV-Fl(2)-L vs. No Primary Control	<0.0001
Mock Infected vs. No Primary Control	0.976
24 hpi	
RSV-Fl(2)-L vs. Mock Infected	<0.0001
RSV-Fl(2)-L vs. No Primary Control	<0.0001
Mock Infected vs. No Primary Control	0.1004

**Graph Title**	M2-1-L PLA # of Puncta
Comparison	P Value
RSV-Fl(2)-L	
1 hpi vs. 6 hpi	>0.9999
1 hpi vs. 8 hpi	0.1268

1 hpi vs. 12 hpi	0.0002
1 hpi vs. 18 hpi	0.0038
1 hpi vs. 24 hpi	<0.0001
6 hpi vs. 8 hpi	0.1429
6 hpi vs. 12 hpi	<0.0001
6 hpi vs. 18 hpi	0.0025
6 hpi vs. 24 hpi	<0.0001
8 hpi vs. 12 hpi	<0.0001
8 hpi vs. 18 hpi	<0.0001
8 hpi vs. 24 hpi	<0.0001
12 hpi vs. 18 hpi	0.9683
12 hpi vs. 24 hpi	<0.0001
18 hpi vs. 24 hpi	<0.0001
Mock Infected	
1 hpi vs. 6 hpi	0.8805
1 hpi vs. 8 hpi	0.5456
1 hpi vs. 12 hpi	0.9998
1 hpi vs. 18 hpi	0.9888
1 hpi vs. 24 hpi	0.73
6 hpi vs. 8 hpi	0.0729
6 hpi vs. 12 hpi	0.9595
6 hpi vs. 18 hpi	0.5264
6 hpi vs. 24 hpi	0.1425
8 hpi vs. 12 hpi	0.3728
8 hpi vs. 18 hpi	0.9006
8 hpi vs. 24 hpi	0.9997
12 hpi vs. 18 hpi	0.9464
12 hpi vs. 24 hpi	0.5556
18 hpi vs. 24 hpi	0.9732
No Primary Control	
1 hpi vs. 6 hpi	>0.9999
1 hpi vs. 8 hpi	0.7804
1 hpi vs. 12 hpi	0.9968
1 hpi vs. 18 hpi	0.9775
1 hpi vs. 24 hpi	0.9994
6 hpi vs. 8 hpi	0.8348
6 hpi vs. 12 hpi	0.992
6 hpi vs. 18 hpi	0.9888
6 hpi vs. 24 hpi	0.9977
8 hpi vs. 12 hpi	0.4768
8 hpi vs. 18 hpi	0.9923
8 hpi vs. 24 hpi	0.5654
12 hpi vs. 18 hpi	0.8295
12 hpi vs. 24 hpi	>0.9999
18 hpi vs. 24 hpi	0.8892

Figure S3C	
**Graph Title**	F vs Pan RSV
Comparison	P Value
Slope of F vs slope of PanRSV	0.0694

Figure S4B	
**Graph Title**	F-L PLA (# of Puncta)
Comparison	P Value

1 hpi	
F-L vs. No Primary Control	<0.0001
F-L vs. Mock Control	<0.0001
No Primary Control vs. Mock Control	0.0452
6 hpi	
F-L vs. No Primary Control	0.3583
F-L vs. Mock Control	0.1781
No Primary Control vs. Mock Control	0.0052
8 hpi	
F-L vs. No Primary Control	0.6898
F-L vs. Mock Control	0.001
No Primary Control vs. Mock Control	<0.0001
**Graph Title**	F-L PLA (Normalized PLA)
Comparison	P Value
1 hpi F-L- No Primary Control	<0.0001
6 F-L- No Primary Control	0.9634
8 F-L- No Primary Control	>0.9999

**Figure S6C**	
**Graph Title**	RSV genome-L PLA (# of Puncta)
Comparison	P Value
6 hpi :RSV genome-L vs. 6 hpi :No Primary Control	0.0018
6 hpi :RSV genome-L vs. 6 hpi :No V5 Control	0.0023
6 hpi :RSV genome-L vs. 6 hpi :Scrambled Control	0.0059
6 hpi :RSV genome-L vs. 6 hpi :Biotin Control	0.0143
6 hpi :RSV genome-L vs. 6 hpi :No FISH Control	<0.0001
6 hpi :RSV genome-L vs. 6 hpi :Mock Control	0.0189
6 hpi :RSV genome-L vs. 8 hpi :RSV genome-L	>0.9999
6 hpi :RSV genome-L vs. 8 hpi :No Primary Control	0.0017
6 hpi :RSV genome-L vs. 8 hpi :No V5 Control	0.0059
6 hpi :RSV genome-L vs. 8 hpi :Scrambled Control	0.0004
6 hpi :RSV genome-L vs. 8 hpi :Biotin Control	0.0322
6 hpi :RSV genome-L vs. 8 hpi :No FISH Control	0.0032
6 hpi :RSV genome-L vs. 8 hpi :Mock Control	0.0004
6 hpi :RSV genome-L vs. 12 hpi :RSV genome-L	<0.0001
6 hpi :RSV genome-L vs. 12 hpi :No Primary Control	0.0032
6 hpi :RSV genome-L vs. 12 hpi :No V5 Control	0.3795
6 hpi :RSV genome-L vs. 12 hpi :Scrambled Control	>0.9999
6 hpi :RSV genome-L vs. 12 hpi :Biotin Control	>0.9999
6 hpi :RSV genome-L vs. 12 hpi :No FISH Control	>0.9999
6 hpi :RSV genome-L vs. 12 hpi :Mock Control	>0.9999
6 hpi :RSV genome-L vs. 18 hpi:RSV genome-L	0.3267
6 hpi :RSV genome-L vs. 18 hpi:No Primary Control	0.0017
6 hpi :RSV genome-L vs. 18 hpi:No V5 Control	0.0059
6 hpi :RSV genome-L vs. 18 hpi:Scrambled Control	>0.9999
6 hpi :RSV genome-L vs. 18 hpi:Biotin Control	>0.9999
6 hpi :RSV genome-L vs. 18 hpi:No FISH Control	0.008
6 hpi :RSV genome-L vs. 18 hpi:Mock Control	0.0851
6 hpi :RSV genome-L vs. 24 hpi :RSV genome-L	<0.0001
6 hpi :RSV genome-L vs. 24 hpi :No Primary Control	0.0059
6 hpi :RSV genome-L vs. 24 hpi :No V5 Control	0.7312
6 hpi :RSV genome-L vs. 24 hpi :Scrambled Control	>0.9999
6 hpi :RSV genome-L vs. 24 hpi :Biotin Control	>0.9999

6 hpi :RSV genome-L vs. 24 hpi :No FISH Control	0.1062
6 hpi :RSV genome-L vs. 24 hpi :Mock Control	0.1062
6 hpi :No Primary Control vs. 6 hpi :No V5 Control	>0.9999
6 hpi :No Primary Control vs. 6 hpi :Scrambled Control	>0.9999
6 hpi :No Primary Control vs. 6 hpi :Biotin Control	>0.9999
6 hpi :No Primary Control vs. 6 hpi :No FISH Control	>0.9999
6 hpi :No Primary Control vs. 6 hpi :Mock Control	>0.9999
6 hpi :No Primary Control vs. 8 hpi :RSV genome-L	0.0047
6 hpi :No Primary Control vs. 8 hpi :No Primary Control	>0.9999
6 hpi :No Primary Control vs. 8 hpi :No V5 Control	>0.9999
6 hpi :No Primary Control vs. 8 hpi :Scrambled Control	>0.9999
6 hpi :No Primary Control vs. 8 hpi :Biotin Control	>0.9999
6 hpi :No Primary Control vs. 8 hpi :No FISH Control	>0.9999
6 hpi :No Primary Control vs. 8 hpi :Mock Control	>0.9999
6 hpi :No Primary Control vs. 12 hpi :RSV genome-L	<0.0001
6 hpi :No Primary Control vs. 12 hpi :No Primary Control	>0.9999
6 hpi :No Primary Control vs. 12 hpi :No V5 Control	0.9998
6 hpi :No Primary Control vs. 12 hpi :Scrambled Control	0.0332
6 hpi :No Primary Control vs. 12 hpi :Biotin Control	0.0045
6 hpi :No Primary Control vs. 12 hpi :No FISH Control	0.0686
6 hpi :No Primary Control vs. 12 hpi :Mock Control	0.0063
6 hpi :No Primary Control vs. 18 hpi:RSV genome-L	<0.0001
6 hpi :No Primary Control vs. 18 hpi:No Primary Control	>0.9999
6 hpi :No Primary Control vs. 18 hpi:No V5 Control	>0.9999
6 hpi :No Primary Control vs. 18 hpi:Scrambled Control	0.2324
6 hpi :No Primary Control vs. 18 hpi:Biotin Control	0.3228
6 hpi :No Primary Control vs. 18 hpi:No FISH Control	>0.9999
6 hpi :No Primary Control vs. 18 hpi:Mock Control	>0.9999
6 hpi :No Primary Control vs. 24 hpi :RSV genome-L	<0.0001
6 hpi :No Primary Control vs. 24 hpi :No Primary Control	>0.9999
6 hpi :No Primary Control vs. 24 hpi :No V5 Control	0.9863
6 hpi :No Primary Control vs. 24 hpi :Scrambled Control	0.3228
6 hpi :No Primary Control vs. 24 hpi :Biotin Control	0.0085
6 hpi :No Primary Control vs. 24 hpi :No FISH Control	>0.9999
6 hpi :No Primary Control vs. 24 hpi :Mock Control	>0.9999
6 hpi :No V5 Control vs. 6 hpi :Scrambled Control	>0.9999
6 hpi :No V5 Control vs. 6 hpi :Biotin Control	>0.9999
6 hpi :No V5 Control vs. 6 hpi :No FISH Control	>0.9999
6 hpi :No V5 Control vs. 6 hpi :Mock Control	>0.9999
6 hpi :No V5 Control vs. 8 hpi :RSV genome-L	0.0059
6 hpi :No V5 Control vs. 8 hpi :No Primary Control	>0.9999
6 hpi :No V5 Control vs. 8 hpi :No V5 Control	>0.9999
6 hpi :No V5 Control vs. 8 hpi :Scrambled Control	>0.9999
6 hpi :No V5 Control vs. 8 hpi :Biotin Control	>0.9999
6 hpi :No V5 Control vs. 8 hpi :No FISH Control	>0.9999
6 hpi :No V5 Control vs. 8 hpi :Mock Control	>0.9999
6 hpi :No V5 Control vs. 12 hpi :RSV genome-L	<0.0001
6 hpi :No V5 Control vs. 12 hpi :No Primary Control	>0.9999
6 hpi :No V5 Control vs. 12 hpi :No V5 Control	>0.9999
6 hpi :No V5 Control vs. 12 hpi :Scrambled Control	0.0416
6 hpi :No V5 Control vs. 12 hpi :Biotin Control	0.0056
6 hpi :No V5 Control vs. 12 hpi :No FISH Control	0.0851
6 hpi :No V5 Control vs. 12 hpi :Mock Control	0.008
6 hpi :No V5 Control vs. 18 hpi:RSV genome-L	<0.0001

6 hpi :No V5 Control vs. 18 hpi:No Primary Control	>0.9999
6 hpi :No V5 Control vs. 18 hpi:No V5 Control	>0.9999
6 hpi :No V5 Control vs. 18 hpi:Scrambled Control	0.278
6 hpi :No V5 Control vs. 18 hpi:Biotin Control	0.3795
6 hpi :No V5 Control vs. 18 hpi:No FISH Control	>0.9999
6 hpi :No V5 Control vs. 18 hpi:Mock Control	>0.9999
6 hpi :No V5 Control vs. 24 hpi :RSV genome-L	<0.0001
6 hpi :No V5 Control vs. 24 hpi :No Primary Control	>0.9999
6 hpi :No V5 Control vs. 24 hpi :No V5 Control	0.9936
6 hpi :No V5 Control vs. 24 hpi :Scrambled Control	0.3795
6 hpi :No V5 Control vs. 24 hpi :Biotin Control	0.0107
6 hpi :No V5 Control vs. 24 hpi :No FISH Control	>0.9999
6 hpi :No V5 Control vs. 24 hpi :Mock Control	>0.9999
6 hpi :Scrambled Control vs. 6 hpi :Biotin Control	>0.9999
6 hpi :Scrambled Control vs. 6 hpi :No FISH Control	>0.9999
6 hpi :Scrambled Control vs. 6 hpi :Mock Control	>0.9999
6 hpi :Scrambled Control vs. 8 hpi :RSV genome-L	0.0143
6 hpi :Scrambled Control vs. 8 hpi :No Primary Control	>0.9999
6 hpi :Scrambled Control vs. 8 hpi :No V5 Control	>0.9999
6 hpi :Scrambled Control vs. 8 hpi :Scrambled Control	>0.9999
6 hpi :Scrambled Control vs. 8 hpi :Biotin Control	>0.9999
6 hpi :Scrambled Control vs. 8 hpi :No FISH Control	>0.9999
6 hpi :Scrambled Control vs. 8 hpi :Mock Control	>0.9999
6 hpi :Scrambled Control vs. 12 hpi :RSV genome-L	<0.0001
6 hpi :Scrambled Control vs. 12 hpi :No Primary Control	>0.9999
6 hpi :Scrambled Control vs. 12 hpi :No V5 Control	>0.9999
6 hpi :Scrambled Control vs. 12 hpi :Scrambled Control	0.0851
6 hpi :Scrambled Control vs. 12 hpi :Biotin Control	0.0135
6 hpi :Scrambled Control vs. 12 hpi :No FISH Control	0.1609
6 hpi :Scrambled Control vs. 12 hpi :Mock Control	0.0189
6 hpi :Scrambled Control vs. 18 hpi:RSV genome-L	<0.0001
6 hpi :Scrambled Control vs. 18 hpi:No Primary Control	>0.9999
6 hpi :Scrambled Control vs. 18 hpi:No V5 Control	>0.9999
6 hpi :Scrambled Control vs. 18 hpi:Scrambled Control	0.4359
6 hpi :Scrambled Control vs. 18 hpi:Biotin Control	0.5554
6 hpi :Scrambled Control vs. 18 hpi:No FISH Control	>0.9999
6 hpi :Scrambled Control vs. 18 hpi:Mock Control	>0.9999
6 hpi :Scrambled Control vs. 24 hpi :RSV genome-L	<0.0001
6 hpi :Scrambled Control vs. 24 hpi :No Primary Control	>0.9999
6 hpi :Scrambled Control vs. 24 hpi :No V5 Control	0.9992
6 hpi :Scrambled Control vs. 24 hpi :Scrambled Control	0.5554
6 hpi :Scrambled Control vs. 24 hpi :Biotin Control	0.0247
6 hpi :Scrambled Control vs. 24 hpi :No FISH Control	>0.9999
6 hpi :Scrambled Control vs. 24 hpi :Mock Control	>0.9999
6 hpi :Biotin Control vs. 6 hpi :No FISH Control	0.9997
6 hpi :Biotin Control vs. 6 hpi :Mock Control	>0.9999
6 hpi :Biotin Control vs. 8 hpi :RSV genome-L	0.0322
6 hpi :Biotin Control vs. 8 hpi :No Primary Control	>0.9999
6 hpi :Biotin Control vs. 8 hpi :No V5 Control	>0.9999
6 hpi :Biotin Control vs. 8 hpi :Scrambled Control	>0.9999
6 hpi :Biotin Control vs. 8 hpi :Biotin Control	>0.9999
6 hpi :Biotin Control vs. 8 hpi :No FISH Control	>0.9999
6 hpi :Biotin Control vs. 8 hpi :Mock Control	>0.9999
6 hpi :Biotin Control vs. 12 hpi :RSV genome-L	<0.0001

6 hpi :Biotin Control vs. 12 hpi :No Primary Control	>0.9999
6 hpi :Biotin Control vs. 12 hpi :No V5 Control	>0.9999
6 hpi :Biotin Control vs. 12 hpi :Scrambled Control	0.1609
6 hpi :Biotin Control vs. 12 hpi :Biotin Control	0.0304
6 hpi :Biotin Control vs. 12 hpi :No FISH Control	0.278
6 hpi :Biotin Control vs. 12 hpi :Mock Control	0.0416
6 hpi :Biotin Control vs. 18 hpi:RSV genome-L	<0.0001
6 hpi :Biotin Control vs. 18 hpi:No Primary Control	>0.9999
6 hpi :Biotin Control vs. 18 hpi:No V5 Control	>0.9999
6 hpi :Biotin Control vs. 18 hpi:Scrambled Control	0.6159
6 hpi :Biotin Control vs. 18 hpi:Biotin Control	0.7312
6 hpi :Biotin Control vs. 18 hpi:No FISH Control	>0.9999
6 hpi :Biotin Control vs. 18 hpi:Mock Control	>0.9999
6 hpi :Biotin Control vs. 24 hpi :RSV genome-L	<0.0001
6 hpi :Biotin Control vs. 24 hpi :No Primary Control	>0.9999
6 hpi :Biotin Control vs. 24 hpi :No V5 Control	>0.9999
6 hpi :Biotin Control vs. 24 hpi :Scrambled Control	0.7312
6 hpi :Biotin Control vs. 24 hpi :Biotin Control	0.0532
6 hpi :Biotin Control vs. 24 hpi :No FISH Control	>0.9999
6 hpi :Biotin Control vs. 24 hpi :Mock Control	>0.9999
6 hpi :No FISH Control vs. 6 hpi :Mock Control	0.9992
6 hpi :No FISH Control vs. 8 hpi :RSV genome-L	<0.0001
6 hpi :No FISH Control vs. 8 hpi :No Primary Control	>0.9999
6 hpi :No FISH Control vs. 8 hpi :No V5 Control	>0.9999
6 hpi :No FISH Control vs. 8 hpi :Scrambled Control	>0.9999
6 hpi :No FISH Control vs. 8 hpi :Biotin Control	0.9966
6 hpi :No FISH Control vs. 8 hpi :No FISH Control	>0.9999
6 hpi :No FISH Control vs. 8 hpi :Mock Control	>0.9999
6 hpi :No FISH Control vs. 12 hpi :RSV genome-L	<0.0001
6 hpi :No FISH Control vs. 12 hpi :No Primary Control	>0.9999
6 hpi :No FISH Control vs. 12 hpi :No V5 Control	0.675
6 hpi :No FISH Control vs. 12 hpi :Scrambled Control	0.0002
6 hpi :No FISH Control vs. 12 hpi :Biotin Control	<0.0001
6 hpi :No FISH Control vs. 12 hpi :No FISH Control	0.0006
6 hpi :No FISH Control vs. 12 hpi :Mock Control	<0.0001
6 hpi :No FISH Control vs. 18 hpi:RSV genome-L	<0.0001
6 hpi :No FISH Control vs. 18 hpi:No Primary Control	>0.9999
6 hpi :No FISH Control vs. 18 hpi:No V5 Control	>0.9999
6 hpi :No FISH Control vs. 18 hpi:Scrambled Control	0.0044
6 hpi :No FISH Control vs. 18 hpi:Biotin Control	0.008
6 hpi :No FISH Control vs. 18 hpi:No FISH Control	>0.9999
6 hpi :No FISH Control vs. 18 hpi:Mock Control	0.9696
6 hpi :No FISH Control vs. 24 hpi :RSV genome-L	<0.0001
6 hpi :No FISH Control vs. 24 hpi :No Primary Control	>0.9999
6 hpi :No FISH Control vs. 24 hpi :No V5 Control	0.3267
6 hpi :No FISH Control vs. 24 hpi :Scrambled Control	0.008
6 hpi :No FISH Control vs. 24 hpi :Biotin Control	<0.0001
6 hpi :No FISH Control vs. 24 hpi :No FISH Control	0.9536
6 hpi :No FISH Control vs. 24 hpi :Mock Control	0.9536
6 hpi :Mock Control vs. 8 hpi :RSV genome-L	0.0416
6 hpi :Mock Control vs. 8 hpi :No Primary Control	>0.9999
6 hpi :Mock Control vs. 8 hpi :No V5 Control	>0.9999
6 hpi :Mock Control vs. 8 hpi :Scrambled Control	>0.9999
6 hpi :Mock Control vs. 8 hpi :Biotin Control	>0.9999

6 hpi :Mock Control vs. 8 hpi :No FISH Control	>0.9999
6 hpi :Mock Control vs. 8 hpi :Mock Control	>0.9999
6 hpi :Mock Control vs. 12 hpi :RSV genome-L	<0.0001
6 hpi :Mock Control vs. 12 hpi :No Primary Control	>0.9999
6 hpi :Mock Control vs. 12 hpi :No V5 Control	>0.9999
6 hpi :Mock Control vs. 12 hpi :Scrambled Control	0.1951
6 hpi :Mock Control vs. 12 hpi :Biotin Control	0.0393
6 hpi :Mock Control vs. 12 hpi :No FISH Control	0.3267
6 hpi :Mock Control vs. 12 hpi :Mock Control	0.0532
6 hpi :Mock Control vs. 18 hpi:RSV genome-L	<0.0001
6 hpi :Mock Control vs. 18 hpi:No Primary Control	>0.9999
6 hpi :Mock Control vs. 18 hpi:No V5 Control	>0.9999
6 hpi :Mock Control vs. 18 hpi:Scrambled Control	0.675
6 hpi :Mock Control vs. 18 hpi:Biotin Control	0.7832
6 hpi :Mock Control vs. 18 hpi:No FISH Control	>0.9999
6 hpi :Mock Control vs. 18 hpi:Mock Control	>0.9999
6 hpi :Mock Control vs. 24 hpi :RSV genome-L	<0.0001
6 hpi :Mock Control vs. 24 hpi :No Primary Control	>0.9999
6 hpi :Mock Control vs. 24 hpi :No V5 Control	>0.9999
6 hpi :Mock Control vs. 24 hpi :Scrambled Control	0.7832
6 hpi :Mock Control vs. 24 hpi :Biotin Control	0.0676
6 hpi :Mock Control vs. 24 hpi :No FISH Control	>0.9999
6 hpi :Mock Control vs. 24 hpi :Mock Control	>0.9999
8 hpi :RSV genome-L vs. 8 hpi :No Primary Control	0.0044
8 hpi :RSV genome-L vs. 8 hpi :No V5 Control	0.0143
8 hpi :RSV genome-L vs. 8 hpi :Scrambled Control	0.0012
8 hpi :RSV genome-L vs. 8 hpi :Biotin Control	0.0676
8 hpi :RSV genome-L vs. 8 hpi :No FISH Control	0.008
8 hpi :RSV genome-L vs. 8 hpi :Mock Control	0.0012
8 hpi :RSV genome-L vs. 12 hpi :RSV genome-L	<0.0001
8 hpi :RSV genome-L vs. 12 hpi :No Primary Control	0.008
8 hpi :RSV genome-L vs. 12 hpi :No V5 Control	0.5554
8 hpi :RSV genome-L vs. 12 hpi :Scrambled Control	>0.9999
8 hpi :RSV genome-L vs. 12 hpi :Biotin Control	>0.9999
8 hpi :RSV genome-L vs. 12 hpi :No FISH Control	>0.9999
8 hpi :RSV genome-L vs. 12 hpi :Mock Control	>0.9999
8 hpi :RSV genome-L vs. 18 hpi:RSV genome-L	0.1951
8 hpi :RSV genome-L vs. 18 hpi:No Primary Control	0.0044
8 hpi :RSV genome-L vs. 18 hpi:No V5 Control	0.0143
8 hpi :RSV genome-L vs. 18 hpi:Scrambled Control	>0.9999
8 hpi :RSV genome-L vs. 18 hpi:Biotin Control	>0.9999
8 hpi :RSV genome-L vs. 18 hpi:No FISH Control	0.0189
8 hpi :RSV genome-L vs. 18 hpi:Mock Control	0.1609
8 hpi :RSV genome-L vs. 24 hpi :RSV genome-L	<0.0001
8 hpi :RSV genome-L vs. 24 hpi :No Primary Control	0.0143
8 hpi :RSV genome-L vs. 24 hpi :No V5 Control	0.8705
8 hpi :RSV genome-L vs. 24 hpi :Scrambled Control	>0.9999
8 hpi :RSV genome-L vs. 24 hpi :Biotin Control	>0.9999
8 hpi :RSV genome-L vs. 24 hpi :No FISH Control	0.1951
8 hpi :RSV genome-L vs. 24 hpi :Mock Control	0.1951
8 hpi :No Primary Control vs. 8 hpi :No V5 Control	>0.9999
8 hpi :No Primary Control vs. 8 hpi :Scrambled Control	>0.9999
8 hpi :No Primary Control vs. 8 hpi :Biotin Control	>0.9999
8 hpi :No Primary Control vs. 8 hpi :No FISH Control	>0.9999

8 hpi :No Primary Control vs. 8 hpi :Mock Control	>0.9999
8 hpi :No Primary Control vs. 12 hpi :RSV genome-L	<0.0001
8 hpi :No Primary Control vs. 12 hpi :No Primary Control	>0.9999
8 hpi :No Primary Control vs. 12 hpi :No V5 Control	0.9999
8 hpi :No Primary Control vs. 12 hpi :Scrambled Control	0.0322
8 hpi :No Primary Control vs. 12 hpi :Biotin Control	0.0042
8 hpi :No Primary Control vs. 12 hpi :No FISH Control	0.0676
8 hpi :No Primary Control vs. 12 hpi :Mock Control	0.0059
8 hpi :No Primary Control vs. 18 hpi:RSV genome-L	<0.0001
8 hpi :No Primary Control vs. 18 hpi:No Primary Control	>0.9999
8 hpi :No Primary Control vs. 18 hpi:No V5 Control	>0.9999
8 hpi :No Primary Control vs. 18 hpi:Scrambled Control	0.2341
8 hpi :No Primary Control vs. 18 hpi:Biotin Control	0.3267
8 hpi :No Primary Control vs. 18 hpi:No FISH Control	>0.9999
8 hpi :No Primary Control vs. 18 hpi:Mock Control	>0.9999
8 hpi :No Primary Control vs. 24 hpi :RSV genome-L	<0.0001
8 hpi :No Primary Control vs. 24 hpi :No Primary Control	>0.9999
8 hpi :No Primary Control vs. 24 hpi :No V5 Control	0.9886
8 hpi :No Primary Control vs. 24 hpi :Scrambled Control	0.3267
8 hpi :No Primary Control vs. 24 hpi :Biotin Control	0.008
8 hpi :No Primary Control vs. 24 hpi :No FISH Control	>0.9999
8 hpi :No Primary Control vs. 24 hpi :Mock Control	>0.9999
8 hpi :No V5 Control vs. 8 hpi :Scrambled Control	>0.9999
8 hpi :No V5 Control vs. 8 hpi :Biotin Control	>0.9999
8 hpi :No V5 Control vs. 8 hpi :No FISH Control	>0.9999
8 hpi :No V5 Control vs. 8 hpi :Mock Control	>0.9999
8 hpi :No V5 Control vs. 12 hpi :RSV genome-L	<0.0001
8 hpi :No V5 Control vs. 12 hpi :No Primary Control	>0.9999
8 hpi :No V5 Control vs. 12 hpi :No V5 Control	>0.9999
8 hpi :No V5 Control vs. 12 hpi :Scrambled Control	0.0851
8 hpi :No V5 Control vs. 12 hpi :Biotin Control	0.0135
8 hpi :No V5 Control vs. 12 hpi :No FISH Control	0.1609
8 hpi :No V5 Control vs. 12 hpi :Mock Control	0.0189
8 hpi :No V5 Control vs. 18 hpi:RSV genome-L	<0.0001
8 hpi :No V5 Control vs. 18 hpi:No Primary Control	>0.9999
8 hpi :No V5 Control vs. 18 hpi:No V5 Control	>0.9999
8 hpi :No V5 Control vs. 18 hpi:Scrambled Control	0.4359
8 hpi :No V5 Control vs. 18 hpi:Biotin Control	0.5554
8 hpi :No V5 Control vs. 18 hpi:No FISH Control	>0.9999
8 hpi :No V5 Control vs. 18 hpi:Mock Control	>0.9999
8 hpi :No V5 Control vs. 24 hpi :RSV genome-L	<0.0001
8 hpi :No V5 Control vs. 24 hpi :No Primary Control	>0.9999
8 hpi :No V5 Control vs. 24 hpi :No V5 Control	0.9992
8 hpi :No V5 Control vs. 24 hpi :Scrambled Control	0.5554
8 hpi :No V5 Control vs. 24 hpi :Biotin Control	0.0247
8 hpi :No V5 Control vs. 24 hpi :No FISH Control	>0.9999
8 hpi :No V5 Control vs. 24 hpi :Mock Control	>0.9999
8 hpi :Scrambled Control vs. 8 hpi :Biotin Control	>0.9999
8 hpi :Scrambled Control vs. 8 hpi :No FISH Control	>0.9999
8 hpi :Scrambled Control vs. 8 hpi :Mock Control	>0.9999
8 hpi :Scrambled Control vs. 12 hpi :RSV genome-L	<0.0001
8 hpi :Scrambled Control vs. 12 hpi :No Primary Control	>0.9999
8 hpi :Scrambled Control vs. 12 hpi :No V5 Control	0.9966
8 hpi :Scrambled Control vs. 12 hpi :Scrambled Control	0.0107

8 hpi :Scrambled Control vs. 12 hpi :Biotin Control	0.0012
8 hpi :Scrambled Control vs. 12 hpi :No FISH Control	0.0247
8 hpi :Scrambled Control vs. 12 hpi :Mock Control	0.0017
8 hpi :Scrambled Control vs. 18 hpi:RSV genome-L	<0.0001
8 hpi :Scrambled Control vs. 18 hpi:No Primary Control	>0.9999
8 hpi :Scrambled Control vs. 18 hpi:No V5 Control	>0.9999
8 hpi :Scrambled Control vs. 18 hpi:Scrambled Control	0.1062
8 hpi :Scrambled Control vs. 18 hpi:Biotin Control	0.1609
8 hpi :Scrambled Control vs. 18 hpi:No FISH Control	>0.9999
8 hpi :Scrambled Control vs. 18 hpi:Mock Control	>0.9999
8 hpi :Scrambled Control vs. 24 hpi :RSV genome-L	<0.0001
8 hpi :Scrambled Control vs. 24 hpi :No Primary Control	>0.9999
8 hpi :Scrambled Control vs. 24 hpi :No V5 Control	0.9322
8 hpi :Scrambled Control vs. 24 hpi :Scrambled Control	0.1609
8 hpi :Scrambled Control vs. 24 hpi :Biotin Control	0.0023
8 hpi :Scrambled Control vs. 24 hpi :No FISH Control	>0.9999
8 hpi :Scrambled Control vs. 24 hpi :Mock Control	>0.9999
8 hpi :Biotin Control vs. 8 hpi :No FISH Control	>0.9999
8 hpi :Biotin Control vs. 8 hpi :Mock Control	>0.9999
8 hpi :Biotin Control vs. 12 hpi :RSV genome-L	<0.0001
8 hpi :Biotin Control vs. 12 hpi :No Primary Control	>0.9999
8 hpi :Biotin Control vs. 12 hpi :No V5 Control	>0.9999
8 hpi :Biotin Control vs. 12 hpi :Scrambled Control	0.278
8 hpi :Biotin Control vs. 12 hpi :Biotin Control	0.0639
8 hpi :Biotin Control vs. 12 hpi :No FISH Control	0.4359
8 hpi :Biotin Control vs. 12 hpi :Mock Control	0.0851
8 hpi :Biotin Control vs. 18 hpi:RSV genome-L	<0.0001
8 hpi :Biotin Control vs. 18 hpi:No Primary Control	>0.9999
8 hpi :Biotin Control vs. 18 hpi:No V5 Control	>0.9999
8 hpi :Biotin Control vs. 18 hpi:Scrambled Control	0.7832
8 hpi :Biotin Control vs. 18 hpi:Biotin Control	0.8705
8 hpi :Biotin Control vs. 18 hpi:No FISH Control	>0.9999
8 hpi :Biotin Control vs. 18 hpi:Mock Control	>0.9999
8 hpi :Biotin Control vs. 24 hpi :RSV genome-L	<0.0001
8 hpi :Biotin Control vs. 24 hpi :No Primary Control	>0.9999
8 hpi :Biotin Control vs. 24 hpi :No V5 Control	>0.9999
8 hpi :Biotin Control vs. 24 hpi :Scrambled Control	0.8705
8 hpi :Biotin Control vs. 24 hpi :Biotin Control	0.1062
8 hpi :Biotin Control vs. 24 hpi :No FISH Control	>0.9999
8 hpi :Biotin Control vs. 24 hpi :Mock Control	>0.9999
8 hpi :No FISH Control vs. 8 hpi :Mock Control	>0.9999
8 hpi :No FISH Control vs. 12 hpi :RSV genome-L	<0.0001
8 hpi :No FISH Control vs. 12 hpi :No Primary Control	>0.9999
8 hpi :No FISH Control vs. 12 hpi :No V5 Control	>0.9999
8 hpi :No FISH Control vs. 12 hpi :Scrambled Control	0.0532
8 hpi :No FISH Control vs. 12 hpi :Biotin Control	0.0076
8 hpi :No FISH Control vs. 12 hpi :No FISH Control	0.1062
8 hpi :No FISH Control vs. 12 hpi :Mock Control	0.0107
8 hpi :No FISH Control vs. 18 hpi:RSV genome-L	<0.0001
8 hpi :No FISH Control vs. 18 hpi:No Primary Control	>0.9999
8 hpi :No FISH Control vs. 18 hpi:No V5 Control	>0.9999
8 hpi :No FISH Control vs. 18 hpi:Scrambled Control	0.3267
8 hpi :No FISH Control vs. 18 hpi:Biotin Control	0.4359
8 hpi :No FISH Control vs. 18 hpi:No FISH Control	>0.9999

8 hpi :No FISH Control vs. 18 hpi:Mock Control	>0.9999
8 hpi :No FISH Control vs. 24 hpi :RSV genome-L	<0.0001
8 hpi :No FISH Control vs. 24 hpi :No Primary Control	>0.9999
8 hpi :No FISH Control vs. 24 hpi :No V5 Control	0.9966
8 hpi :No FISH Control vs. 24 hpi :Scrambled Control	0.4359
8 hpi :No FISH Control vs. 24 hpi :Biotin Control	0.0143
8 hpi :No FISH Control vs. 24 hpi :No FISH Control	>0.9999
8 hpi :No FISH Control vs. 24 hpi :Mock Control	>0.9999
8 hpi :Mock Control vs. 12 hpi :RSV genome-L	<0.0001
8 hpi :Mock Control vs. 12 hpi :No Primary Control	>0.9999
8 hpi :Mock Control vs. 12 hpi :No V5 Control	0.9966
8 hpi :Mock Control vs. 12 hpi :Scrambled Control	0.0107
8 hpi :Mock Control vs. 12 hpi :Biotin Control	0.0012
8 hpi :Mock Control vs. 12 hpi :No FISH Control	0.0247
8 hpi :Mock Control vs. 12 hpi :Mock Control	0.0017
8 hpi :Mock Control vs. 18 hpi:RSV genome-L	<0.0001
8 hpi :Mock Control vs. 18 hpi:No Primary Control	>0.9999
8 hpi :Mock Control vs. 18 hpi:No V5 Control	>0.9999
8 hpi :Mock Control vs. 18 hpi:Scrambled Control	0.1062
8 hpi :Mock Control vs. 18 hpi:Biotin Control	0.1609
8 hpi :Mock Control vs. 18 hpi:No FISH Control	>0.9999
8 hpi :Mock Control vs. 18 hpi:Mock Control	>0.9999
8 hpi :Mock Control vs. 24 hpi :RSV genome-L	<0.0001
8 hpi :Mock Control vs. 24 hpi :No Primary Control	>0.9999
8 hpi :Mock Control vs. 24 hpi :No V5 Control	0.9322
8 hpi :Mock Control vs. 24 hpi :Scrambled Control	0.1609
8 hpi :Mock Control vs. 24 hpi :Biotin Control	0.0023
8 hpi :Mock Control vs. 24 hpi :No FISH Control	>0.9999
8 hpi :Mock Control vs. 24 hpi :Mock Control	>0.9999
12 hpi :RSV genome-L vs. 12 hpi :No Primary Control	<0.0001
12 hpi :RSV genome-L vs. 12 hpi :No V5 Control	<0.0001
12 hpi :RSV genome-L vs. 12 hpi :Scrambled Control	<0.0001
12 hpi :RSV genome-L vs. 12 hpi :Biotin Control	<0.0001
12 hpi :RSV genome-L vs. 12 hpi :No FISH Control	<0.0001
12 hpi :RSV genome-L vs. 12 hpi :Mock Control	<0.0001
12 hpi :RSV genome-L vs. 18 hpi:RSV genome-L	<0.0001
12 hpi :RSV genome-L vs. 18 hpi:No Primary Control	<0.0001
12 hpi :RSV genome-L vs. 18 hpi:No V5 Control	<0.0001
12 hpi :RSV genome-L vs. 18 hpi:Scrambled Control	<0.0001
12 hpi :RSV genome-L vs. 18 hpi:Biotin Control	<0.0001
12 hpi :RSV genome-L vs. 18 hpi:No FISH Control	<0.0001
12 hpi :RSV genome-L vs. 18 hpi:Mock Control	<0.0001
12 hpi :RSV genome-L vs. 24 hpi :RSV genome-L	<0.0001
12 hpi :RSV genome-L vs. 24 hpi :No Primary Control	<0.0001
12 hpi :RSV genome-L vs. 24 hpi :No V5 Control	<0.0001
12 hpi :RSV genome-L vs. 24 hpi :Scrambled Control	<0.0001
12 hpi :RSV genome-L vs. 24 hpi :Biotin Control	<0.0001
12 hpi :RSV genome-L vs. 24 hpi :No FISH Control	<0.0001
12 hpi :RSV genome-L vs. 24 hpi :Mock Control	<0.0001
12 hpi :No Primary Control vs. 12 hpi :No V5 Control	>0.9999
12 hpi :No Primary Control vs. 12 hpi :Scrambled Control	0.0532
12 hpi :No Primary Control vs. 12 hpi :Biotin Control	0.0076
12 hpi :No Primary Control vs. 12 hpi :No FISH Control	0.1062
12 hpi :No Primary Control vs. 12 hpi :Mock Control	0.0107

12 hpi :No Primary Control vs. 18 hpi:RSV genome-L	<0.0001
12 hpi :No Primary Control vs. 18 hpi:No Primary Control	>0.9999
12 hpi :No Primary Control vs. 18 hpi:No V5 Control	>0.9999
12 hpi :No Primary Control vs. 18 hpi:Scrambled Control	0.3267
12 hpi :No Primary Control vs. 18 hpi:Biotin Control	0.4359
12 hpi :No Primary Control vs. 18 hpi:No FISH Control	>0.9999
12 hpi :No Primary Control vs. 18 hpi:Mock Control	>0.9999
12 hpi :No Primary Control vs. 24 hpi :RSV genome-L	<0.0001
12 hpi :No Primary Control vs. 24 hpi :No Primary Control	>0.9999
12 hpi :No Primary Control vs. 24 hpi :No V5 Control	0.9966
12 hpi :No Primary Control vs. 24 hpi :Scrambled Control	0.4359
12 hpi :No Primary Control vs. 24 hpi :Biotin Control	0.0143
12 hpi :No Primary Control vs. 24 hpi :No FISH Control	>0.9999
12 hpi :No Primary Control vs. 24 hpi :Mock Control	>0.9999
12 hpi :No V5 Control vs. 12 hpi :Scrambled Control	0.9046
12 hpi :No V5 Control vs. 12 hpi :Biotin Control	0.5346
12 hpi :No V5 Control vs. 12 hpi :No FISH Control	0.9696
12 hpi :No V5 Control vs. 12 hpi :Mock Control	0.6159
12 hpi :No V5 Control vs. 18 hpi:RSV genome-L	<0.0001
12 hpi :No V5 Control vs. 18 hpi:No Primary Control	0.9999
12 hpi :No V5 Control vs. 18 hpi:No V5 Control	>0.9999
12 hpi :No V5 Control vs. 18 hpi:Scrambled Control	0.9992
12 hpi :No V5 Control vs. 18 hpi:Biotin Control	0.9999
12 hpi :No V5 Control vs. 18 hpi:No FISH Control	>0.9999
12 hpi :No V5 Control vs. 18 hpi:Mock Control	>0.9999
12 hpi :No V5 Control vs. 24 hpi :RSV genome-L	<0.0001
12 hpi :No V5 Control vs. 24 hpi :No Primary Control	>0.9999
12 hpi :No V5 Control vs. 24 hpi :No V5 Control	>0.9999
12 hpi :No V5 Control vs. 24 hpi :Scrambled Control	0.9999
12 hpi :No V5 Control vs. 24 hpi :Biotin Control	0.675
12 hpi :No V5 Control vs. 24 hpi :No FISH Control	>0.9999
12 hpi :No V5 Control vs. 24 hpi :Mock Control	>0.9999
12 hpi :Scrambled Control vs. 12 hpi :Biotin Control	>0.9999
12 hpi :Scrambled Control vs. 12 hpi :No FISH Control	>0.9999
12 hpi :Scrambled Control vs. 12 hpi :Mock Control	>0.9999
12 hpi :Scrambled Control vs. 18 hpi:RSV genome-L	0.0416
12 hpi :Scrambled Control vs. 18 hpi:No Primary Control	0.0322
12 hpi :Scrambled Control vs. 18 hpi:No V5 Control	0.0851
12 hpi :Scrambled Control vs. 18 hpi:Scrambled Control	>0.9999
12 hpi :Scrambled Control vs. 18 hpi:Biotin Control	>0.9999
12 hpi :Scrambled Control vs. 18 hpi:No FISH Control	0.1062
12 hpi :Scrambled Control vs. 18 hpi:Mock Control	0.4949
12 hpi :Scrambled Control vs. 24 hpi :RSV genome-L	<0.0001
12 hpi :Scrambled Control vs. 24 hpi :No Primary Control	0.0851
12 hpi :Scrambled Control vs. 24 hpi :No V5 Control	0.9936
12 hpi :Scrambled Control vs. 24 hpi :Scrambled Control	>0.9999
12 hpi :Scrambled Control vs. 24 hpi :Biotin Control	>0.9999
12 hpi :Scrambled Control vs. 24 hpi :No FISH Control	0.5554
12 hpi :Scrambled Control vs. 24 hpi :Mock Control	0.5554
12 hpi :Biotin Control vs. 12 hpi :No FISH Control	>0.9999
12 hpi :Biotin Control vs. 12 hpi :Mock Control	>0.9999
12 hpi :Biotin Control vs. 18 hpi:RSV genome-L	0.2376
12 hpi :Biotin Control vs. 18 hpi:No Primary Control	0.0042
12 hpi :Biotin Control vs. 18 hpi:No V5 Control	0.0135

12 hpi :Biotin Control vs. 18 hpi:Scrambled Control	>0.9999
12 hpi :Biotin Control vs. 18 hpi:Biotin Control	>0.9999
12 hpi :Biotin Control vs. 18 hpi:No FISH Control	0.0179
12 hpi :Biotin Control vs. 18 hpi:Mock Control	0.1523
12 hpi :Biotin Control vs. 24 hpi :RSV genome-L	<0.0001
12 hpi :Biotin Control vs. 24 hpi :No Primary Control	0.0135
12 hpi :Biotin Control vs. 24 hpi :No V5 Control	0.8553
12 hpi :Biotin Control vs. 24 hpi :Scrambled Control	>0.9999
12 hpi :Biotin Control vs. 24 hpi :Biotin Control	>0.9999
12 hpi :Biotin Control vs. 24 hpi :No FISH Control	0.1849
12 hpi :Biotin Control vs. 24 hpi :Mock Control	0.1849
12 hpi :No FISH Control vs. 12 hpi :Mock Control	>0.9999
12 hpi :No FISH Control vs. 18 hpi:RSV genome-L	0.0189
12 hpi :No FISH Control vs. 18 hpi:No Primary Control	0.0676
12 hpi :No FISH Control vs. 18 hpi:No V5 Control	0.1609
12 hpi :No FISH Control vs. 18 hpi:Scrambled Control	>0.9999
12 hpi :No FISH Control vs. 18 hpi:Biotin Control	>0.9999
12 hpi :No FISH Control vs. 18 hpi:No FISH Control	0.1951
12 hpi :No FISH Control vs. 18 hpi:Mock Control	0.675
12 hpi :No FISH Control vs. 24 hpi :RSV genome-L	<0.0001
12 hpi :No FISH Control vs. 24 hpi :No Primary Control	0.1609
12 hpi :No FISH Control vs. 24 hpi :No V5 Control	0.9992
12 hpi :No FISH Control vs. 24 hpi :Scrambled Control	>0.9999
12 hpi :No FISH Control vs. 24 hpi :Biotin Control	>0.9999
12 hpi :No FISH Control vs. 24 hpi :No FISH Control	0.7312
12 hpi :No FISH Control vs. 24 hpi :Mock Control	0.7312
12 hpi :Mock Control vs. 18 hpi:RSV genome-L	0.1609
12 hpi :Mock Control vs. 18 hpi:No Primary Control	0.0059
12 hpi :Mock Control vs. 18 hpi:No V5 Control	0.0189
12 hpi :Mock Control vs. 18 hpi:Scrambled Control	>0.9999
12 hpi :Mock Control vs. 18 hpi:Biotin Control	>0.9999
12 hpi :Mock Control vs. 18 hpi:No FISH Control	0.0247
12 hpi :Mock Control vs. 18 hpi:Mock Control	0.1951
12 hpi :Mock Control vs. 24 hpi :RSV genome-L	<0.0001
12 hpi :Mock Control vs. 24 hpi :No Primary Control	0.0189
12 hpi :Mock Control vs. 24 hpi :No V5 Control	0.9046
12 hpi :Mock Control vs. 24 hpi :Scrambled Control	>0.9999
12 hpi :Mock Control vs. 24 hpi :Biotin Control	>0.9999
12 hpi :Mock Control vs. 24 hpi :No FISH Control	0.2341
12 hpi :Mock Control vs. 24 hpi :Mock Control	0.2341
18 hpi:RSV genome-L vs. 18 hpi:No Primary Control	<0.0001
18 hpi:RSV genome-L vs. 18 hpi:No V5 Control	<0.0001
18 hpi:RSV genome-L vs. 18 hpi:Scrambled Control	0.0032
18 hpi:RSV genome-L vs. 18 hpi:Biotin Control	0.0017
18 hpi:RSV genome-L vs. 18 hpi:No FISH Control	<0.0001
18 hpi:RSV genome-L vs. 18 hpi:Mock Control	<0.0001
18 hpi:RSV genome-L vs. 24 hpi :RSV genome-L	<0.0001
18 hpi:RSV genome-L vs. 24 hpi :No Primary Control	<0.0001
18 hpi:RSV genome-L vs. 24 hpi :No V5 Control	<0.0001
18 hpi:RSV genome-L vs. 24 hpi :Scrambled Control	0.0017
18 hpi:RSV genome-L vs. 24 hpi :Biotin Control	0.1314
18 hpi:RSV genome-L vs. 24 hpi :No FISH Control	<0.0001
18 hpi:RSV genome-L vs. 24 hpi :Mock Control	<0.0001
18 hpi:No Primary Control vs. 18 hpi:No V5 Control	>0.9999

18 hpi:No Primary Control vs. 18 hpi:Scrambled Control	0.2341
18 hpi:No Primary Control vs. 18 hpi:Biotin Control	0.3267
18 hpi:No Primary Control vs. 18 hpi:No FISH Control	>0.9999
18 hpi:No Primary Control vs. 18 hpi:Mock Control	>0.9999
18 hpi:No Primary Control vs. 24 hpi :RSV genome-L	<0.0001
18 hpi:No Primary Control vs. 24 hpi :No Primary Control	>0.9999
18 hpi:No Primary Control vs. 24 hpi :No V5 Control	0.9886
18 hpi:No Primary Control vs. 24 hpi :Scrambled Control	0.3267
18 hpi:No Primary Control vs. 24 hpi :Biotin Control	0.008
18 hpi:No Primary Control vs. 24 hpi :No FISH Control	>0.9999
18 hpi:No Primary Control vs. 24 hpi :Mock Control	>0.9999
18 hpi:No V5 Control vs. 18 hpi:Scrambled Control	0.4359
18 hpi:No V5 Control vs. 18 hpi:Biotin Control	0.5554
18 hpi:No V5 Control vs. 18 hpi:No FISH Control	>0.9999
18 hpi:No V5 Control vs. 18 hpi:Mock Control	>0.9999
18 hpi:No V5 Control vs. 24 hpi :RSV genome-L	<0.0001
18 hpi:No V5 Control vs. 24 hpi :No Primary Control	>0.9999
18 hpi:No V5 Control vs. 24 hpi :No V5 Control	0.9992
18 hpi:No V5 Control vs. 24 hpi :Scrambled Control	0.5554
18 hpi:No V5 Control vs. 24 hpi :Biotin Control	0.0247
18 hpi:No V5 Control vs. 24 hpi :No FISH Control	>0.9999
18 hpi:No V5 Control vs. 24 hpi :Mock Control	>0.9999
18 hpi:Scrambled Control vs. 18 hpi:Biotin Control	>0.9999
18 hpi:Scrambled Control vs. 18 hpi:No FISH Control	0.4949
18 hpi:Scrambled Control vs. 18 hpi:Mock Control	0.9322
18 hpi:Scrambled Control vs. 24 hpi :RSV genome-L	<0.0001
18 hpi:Scrambled Control vs. 24 hpi :No Primary Control	0.4359
18 hpi:Scrambled Control vs. 24 hpi :No V5 Control	>0.9999
18 hpi:Scrambled Control vs. 24 hpi :Scrambled Control	>0.9999
18 hpi:Scrambled Control vs. 24 hpi :Biotin Control	>0.9999
18 hpi:Scrambled Control vs. 24 hpi :No FISH Control	0.9536
18 hpi:Scrambled Control vs. 24 hpi :Mock Control	0.9536
18 hpi:Biotin Control vs. 18 hpi:No FISH Control	0.6159
18 hpi:Biotin Control vs. 18 hpi:Mock Control	0.9696
18 hpi:Biotin Control vs. 24 hpi :RSV genome-L	<0.0001
18 hpi:Biotin Control vs. 24 hpi :No Primary Control	0.5554
18 hpi:Biotin Control vs. 24 hpi :No V5 Control	>0.9999
18 hpi:Biotin Control vs. 24 hpi :Scrambled Control	>0.9999
18 hpi:Biotin Control vs. 24 hpi :Biotin Control	>0.9999
18 hpi:Biotin Control vs. 24 hpi :No FISH Control	0.981
18 hpi:Biotin Control vs. 24 hpi :Mock Control	0.981
18 hpi:No FISH Control vs. 18 hpi:Mock Control	>0.9999
18 hpi:No FISH Control vs. 24 hpi :RSV genome-L	<0.0001
18 hpi:No FISH Control vs. 24 hpi :No Primary Control	>0.9999
18 hpi:No FISH Control vs. 24 hpi :No V5 Control	0.9997
18 hpi:No FISH Control vs. 24 hpi :Scrambled Control	0.6159
18 hpi:No FISH Control vs. 24 hpi :Biotin Control	0.0322
18 hpi:No FISH Control vs. 24 hpi :No FISH Control	>0.9999
18 hpi:No FISH Control vs. 24 hpi :Mock Control	>0.9999
18 hpi:Mock Control vs. 24 hpi :RSV genome-L	<0.0001
18 hpi:Mock Control vs. 24 hpi :No Primary Control	>0.9999
18 hpi:Mock Control vs. 24 hpi :No V5 Control	>0.9999
18 hpi:Mock Control vs. 24 hpi :Scrambled Control	0.9696
18 hpi:Mock Control vs. 24 hpi :Biotin Control	0.2341

18 hpi:Mock Control vs. 24 hpi :No FISH Control	>0.9999
18 hpi:Mock Control vs. 24 hpi :Mock Control	>0.9999
24 hpi :RSV genome-L vs. 24 hpi :No Primary Control	<0.0001
24 hpi :RSV genome-L vs. 24 hpi :No V5 Control	<0.0001
24 hpi :RSV genome-L vs. 24 hpi :Scrambled Control	<0.0001
24 hpi :RSV genome-L vs. 24 hpi :Biotin Control	<0.0001
24 hpi :RSV genome-L vs. 24 hpi :No FISH Control	<0.0001
24 hpi :RSV genome-L vs. 24 hpi :Mock Control	<0.0001
24 hpi :No Primary Control vs. 24 hpi :No V5 Control	0.9992
24 hpi :No Primary Control vs. 24 hpi :Scrambled Control	0.5554
24 hpi :No Primary Control vs. 24 hpi :Biotin Control	0.0247
24 hpi :No Primary Control vs. 24 hpi :No FISH Control	>0.9999
24 hpi :No Primary Control vs. 24 hpi :Mock Control	>0.9999
24 hpi :No V5 Control vs. 24 hpi :Scrambled Control	>0.9999
24 hpi :No V5 Control vs. 24 hpi :Biotin Control	0.9322
24 hpi :No V5 Control vs. 24 hpi :No FISH Control	>0.9999
24 hpi :No V5 Control vs. 24 hpi :Mock Control	>0.9999
24 hpi :Scrambled Control vs. 24 hpi :Biotin Control	>0.9999
24 hpi :Scrambled Control vs. 24 hpi :No FISH Control	0.981
24 hpi :Scrambled Control vs. 24 hpi :Mock Control	0.981
24 hpi :Biotin Control vs. 24 hpi :No FISH Control	0.278
24 hpi :Biotin Control vs. 24 hpi :Mock Control	0.278
24 hpi :No FISH Control vs. 24 hpi :Mock Control	>0.9999
**Graph Title**	RSV genome-L PLA (Normalized PLA)
Comparison	P Value
6 hpi	
RSV genome-L vs. No Primary Control	0.0003
RSV genome-L vs. No V5 Control	<0.0001
RSV genome-L vs. Scrambled Control	<0.0001
RSV genome-L vs. Biotin Control	<0.0001
RSV genome-L vs. No FISH Control	<0.0001
No Primary Control vs. No V5 Control	0.895
No Primary Control vs. Scrambled Control	0.9837
No Primary Control vs. Biotin Control	0.9998
No Primary Control vs. No FISH Control	0.8005
No V5 Control vs. Scrambled Control	0.9989
No V5 Control vs. Biotin Control	0.9676
No V5 Control vs. No FISH Control	>0.9999
Scrambled Control vs. Biotin Control	0.9985
Scrambled Control vs. No FISH Control	0.9912
Biotin Control vs. No FISH Control	0.9148
8 hpi	
RSV genome-L vs. No Primary Control	<0.0001
RSV genome-L vs. No V5 Control	<0.0001
RSV genome-L vs. Scrambled Control	<0.0001
RSV genome-L vs. Biotin Control	0.0006
RSV genome-L vs. No FISH Control	<0.0001
No Primary Control vs. No V5 Control	0.9994
No Primary Control vs. Scrambled Control	0.9997
No Primary Control vs. Biotin Control	0.9945
No Primary Control vs. No FISH Control	>0.9999
No V5 Control vs. Scrambled Control	>0.9999
No V5 Control vs. Biotin Control	0.9518

No V5 Control vs. No FISH Control	0.9977
Scrambled Control vs. Biotin Control	0.9605
Scrambled Control vs. No FISH Control	0.9985
Biotin Control vs. No FISH Control	0.9982
12 hpi	
RSV genome-L vs. No Primary Control	<0.0001
RSV genome-L vs. No V5 Control	<0.0001
RSV genome-L vs. Scrambled Control	<0.0001
RSV genome-L vs. Biotin Control	<0.0001
RSV genome-L vs. No FISH Control	<0.0001
No Primary Control vs. No V5 Control	0.9891
No Primary Control vs. Scrambled Control	0.057
No Primary Control vs. Biotin Control	0.013
No Primary Control vs. No FISH Control	0.9978
No V5 Control vs. Scrambled Control	0.2467
No V5 Control vs. Biotin Control	0.0823
No V5 Control vs. No FISH Control	>0.9999
Scrambled Control vs. Biotin Control	0.9966
Scrambled Control vs. No FISH Control	0.17
Biotin Control vs. No FISH Control	0.0506
18 hpi	
RSV genome-L vs. No Primary Control	0.9997
RSV genome-L vs. No V5 Control	0.9939
RSV genome-L vs. Scrambled Control	0.9944
RSV genome-L vs. Biotin Control	0.9843
RSV genome-L vs. No FISH Control	0.9936
No Primary Control vs. No V5 Control	0.9998
No Primary Control vs. Scrambled Control	0.9998
No Primary Control vs. Biotin Control	0.9988
No Primary Control vs. No FISH Control	0.9998
No V5 Control vs. Scrambled Control	>0.9999
No V5 Control vs. Biotin Control	>0.9999
No V5 Control vs. No FISH Control	>0.9999
Scrambled Control vs. Biotin Control	>0.9999
Scrambled Control vs. No FISH Control	>0.9999
Biotin Control vs. No FISH Control	>0.9999
24 hpi	
RSV genome-L vs. No Primary Control	>0.9999
RSV genome-L vs. No V5 Control	0.9891
RSV genome-L vs. Scrambled Control	0.9824
RSV genome-L vs. Biotin Control	0.533
RSV genome-L vs. No FISH Control	0.9992
No Primary Control vs. No V5 Control	0.959
No Primary Control vs. Scrambled Control	0.943
No Primary Control vs. Biotin Control	0.3923
No Primary Control vs. No FISH Control	0.9925
No V5 Control vs. Scrambled Control	>0.9999
No V5 Control vs. Biotin Control	0.8924
No V5 Control vs. No FISH Control	0.9998
Scrambled Control vs. Biotin Control	0.9169
Scrambled Control vs. No FISH Control	0.9994
Biotin Control vs. No FISH Control	0.7627

Figure S7B

**Graph Title**	NS1 mRNA-L PLA (# of Puncta)
Comparison	P Value
6 hpi :NS1 mRNA-L vs. 6 hpi :No Primary Control	<0.0001
6 hpi :NS1 mRNA-L vs. 6 hpi :No V5 Control	<0.0001
6 hpi :NS1 mRNA-L vs. 6 hpi :Scrambled Control	<0.0001
6 hpi :NS1 mRNA-L vs. 6 hpi :Biotin Control	<0.0001
6 hpi :NS1 mRNA-L vs. 6 hpi :No FISH Control	<0.0001
6 hpi :NS1 mRNA-L vs. 6 hpi :Mock Control	<0.0001
6 hpi :NS1 mRNA-L vs. 8 hpi :NS1 mRNA-L	<0.0001
6 hpi :NS1 mRNA-L vs. 8 hpi :No Primary Control	<0.0001
6 hpi :NS1 mRNA-L vs. 8 hpi :No V5 Control	<0.0001
6 hpi :NS1 mRNA-L vs. 8 hpi :Scrambled Control	<0.0001
6 hpi :NS1 mRNA-L vs. 8 hpi :Biotin Control	<0.0001
6 hpi :NS1 mRNA-L vs. 8 hpi :No FISH Control	<0.0001
6 hpi :NS1 mRNA-L vs. 8 hpi :Mock Control	<0.0001
6 hpi :NS1 mRNA-L vs. 12 hpi :NS1 mRNA-L	0.0076
6 hpi :NS1 mRNA-L vs. 12 hpi :No Primary Control	<0.0001
6 hpi :NS1 mRNA-L vs. 12 hpi :No V5 Control	<0.0001
6 hpi :NS1 mRNA-L vs. 12 hpi :Scrambled Control	<0.0001
6 hpi :NS1 mRNA-L vs. 12 hpi :Biotin Control	<0.0001
6 hpi :NS1 mRNA-L vs. 12 hpi :No FISH Control	<0.0001
6 hpi :NS1 mRNA-L vs. 12 hpi :Mock Control	>0.9999
6 hpi :NS1 mRNA-L vs. 18 hpi:NS1 mRNA-L	0.0002
6 hpi :NS1 mRNA-L vs. 18 hpi:No Primary Control	<0.0001
6 hpi :NS1 mRNA-L vs. 18 hpi:No V5 Control	<0.0001
6 hpi :NS1 mRNA-L vs. 18 hpi:Scrambled Control	<0.0001
6 hpi :NS1 mRNA-L vs. 18 hpi:Biotin Control	<0.0001
6 hpi :NS1 mRNA-L vs. 18 hpi:No FISH Control	<0.0001
6 hpi :NS1 mRNA-L vs. 18 hpi:Mock Control	<0.0001
6 hpi :NS1 mRNA-L vs. 24 hpi :NS1 mRNA-L	0.9998
6 hpi :NS1 mRNA-L vs. 24 hpi :No Primary Control	<0.0001
6 hpi :NS1 mRNA-L vs. 24 hpi :No V5 Control	<0.0001
6 hpi :NS1 mRNA-L vs. 24 hpi :Scrambled Control	<0.0001
6 hpi :NS1 mRNA-L vs. 24 hpi :Biotin Control	<0.0001
6 hpi :NS1 mRNA-L vs. 24 hpi :No FISH Control	<0.0001
6 hpi :NS1 mRNA-L vs. 24 hpi :Mock Control	<0.0001
6 hpi :No Primary Control vs. 6 hpi :No V5 Control	>0.9999
6 hpi :No Primary Control vs. 6 hpi :Scrambled Control	>0.9999
6 hpi :No Primary Control vs. 6 hpi :Biotin Control	>0.9999
6 hpi :No Primary Control vs. 6 hpi :No FISH Control	>0.9999
6 hpi :No Primary Control vs. 6 hpi :Mock Control	0.1407
6 hpi :No Primary Control vs. 8 hpi :NS1 mRNA-L	<0.0001
6 hpi :No Primary Control vs. 8 hpi :No Primary Control	>0.9999
6 hpi :No Primary Control vs. 8 hpi :No V5 Control	>0.9999
6 hpi :No Primary Control vs. 8 hpi :Scrambled Control	>0.9999
6 hpi :No Primary Control vs. 8 hpi :Biotin Control	0.9996
6 hpi :No Primary Control vs. 8 hpi :No FISH Control	>0.9999
6 hpi :No Primary Control vs. 8 hpi :Mock Control	0.0011
6 hpi :No Primary Control vs. 12 hpi :NS1 mRNA-L	<0.0001
6 hpi :No Primary Control vs. 12 hpi :No Primary Control	>0.9999
6 hpi :No Primary Control vs. 12 hpi :No V5 Control	0.7223
6 hpi :No Primary Control vs. 12 hpi :Scrambled Control	0.2336
6 hpi :No Primary Control vs. 12 hpi :Biotin Control	0.062
6 hpi :No Primary Control vs. 12 hpi :No FISH Control	0.3602

6 hpi :No Primary Control vs. 12 hpi :Mock Control	<0.0001
6 hpi :No Primary Control vs. 18 hpi:NS1 mRNA-L	<0.0001
6 hpi :No Primary Control vs. 18 hpi:No Primary Control	>0.9999
6 hpi :No Primary Control vs. 18 hpi:No V5 Control	>0.9999
6 hpi :No Primary Control vs. 18 hpi:Scrambled Control	0.6722
6 hpi :No Primary Control vs. 18 hpi:Biotin Control	0.7692
6 hpi :No Primary Control vs. 18 hpi:No FISH Control	>0.9999
6 hpi :No Primary Control vs. 18 hpi:Mock Control	0.0002
6 hpi :No Primary Control vs. 24 hpi :NS1 mRNA-L	<0.0001
6 hpi :No Primary Control vs. 24 hpi :No Primary Control	>0.9999
6 hpi :No Primary Control vs. 24 hpi :No V5 Control	>0.9999
6 hpi :No Primary Control vs. 24 hpi :Scrambled Control	0.7692
6 hpi :No Primary Control vs. 24 hpi :Biotin Control	0.0966
6 hpi :No Primary Control vs. 24 hpi :No FISH Control	>0.9999
6 hpi :No Primary Control vs. 24 hpi :Mock Control	0.8841
6 hpi :No V5 Control vs. 6 hpi :Scrambled Control	>0.9999
6 hpi :No V5 Control vs. 6 hpi :Biotin Control	>0.9999
6 hpi :No V5 Control vs. 6 hpi :No FISH Control	0.9993
6 hpi :No V5 Control vs. 6 hpi :Mock Control	0.46
6 hpi :No V5 Control vs. 8 hpi :NS1 mRNA-L	<0.0001
6 hpi :No V5 Control vs. 8 hpi :No Primary Control	>0.9999
6 hpi :No V5 Control vs. 8 hpi :No V5 Control	>0.9999
6 hpi :No V5 Control vs. 8 hpi :Scrambled Control	>0.9999
6 hpi :No V5 Control vs. 8 hpi :Biotin Control	>0.9999
6 hpi :No V5 Control vs. 8 hpi :No FISH Control	>0.9999
6 hpi :No V5 Control vs. 8 hpi :Mock Control	0.0099
6 hpi :No V5 Control vs. 12 hpi :NS1 mRNA-L	<0.0001
6 hpi :No V5 Control vs. 12 hpi :No Primary Control	>0.9999
6 hpi :No V5 Control vs. 12 hpi :No V5 Control	0.9684
6 hpi :No V5 Control vs. 12 hpi :Scrambled Control	0.6199
6 hpi :No V5 Control vs. 12 hpi :Biotin Control	0.2622
6 hpi :No V5 Control vs. 12 hpi :No FISH Control	0.7692
6 hpi :No V5 Control vs. 12 hpi :Mock Control	<0.0001
6 hpi :No V5 Control vs. 18 hpi:NS1 mRNA-L	<0.0001
6 hpi :No V5 Control vs. 18 hpi:No Primary Control	>0.9999
6 hpi :No V5 Control vs. 18 hpi:No V5 Control	>0.9999
6 hpi :No V5 Control vs. 18 hpi:Scrambled Control	0.9542
6 hpi :No V5 Control vs. 18 hpi:Biotin Control	0.979
6 hpi :No V5 Control vs. 18 hpi:No FISH Control	>0.9999
6 hpi :No V5 Control vs. 18 hpi:Mock Control	0.0019
6 hpi :No V5 Control vs. 24 hpi :NS1 mRNA-L	<0.0001
6 hpi :No V5 Control vs. 24 hpi :No Primary Control	>0.9999
6 hpi :No V5 Control vs. 24 hpi :No V5 Control	>0.9999
6 hpi :No V5 Control vs. 24 hpi :Scrambled Control	0.979
6 hpi :No V5 Control vs. 24 hpi :Biotin Control	0.3602
6 hpi :No V5 Control vs. 24 hpi :No FISH Control	>0.9999
6 hpi :No V5 Control vs. 24 hpi :Mock Control	0.9951
6 hpi :Scrambled Control vs. 6 hpi :Biotin Control	>0.9999
6 hpi :Scrambled Control vs. 6 hpi :No FISH Control	>0.9999
6 hpi :Scrambled Control vs. 6 hpi :Mock Control	0.1679
6 hpi :Scrambled Control vs. 8 hpi :NS1 mRNA-L	<0.0001
6 hpi :Scrambled Control vs. 8 hpi :No Primary Control	>0.9999
6 hpi :Scrambled Control vs. 8 hpi :No V5 Control	>0.9999
6 hpi :Scrambled Control vs. 8 hpi :Scrambled Control	>0.9999

6 hpi :Scrambled Control vs. 8 hpi :Biotin Control	0.9998
6 hpi :Scrambled Control vs. 8 hpi :No FISH Control	>0.9999
6 hpi :Scrambled Control vs. 8 hpi :Mock Control	0.0014
6 hpi :Scrambled Control vs. 12 hpi :NS1 mRNA-L	<0.0001
6 hpi :Scrambled Control vs. 12 hpi :No Primary Control	>0.9999
6 hpi :Scrambled Control vs. 12 hpi :No V5 Control	0.7692
6 hpi :Scrambled Control vs. 12 hpi :Scrambled Control	0.2722
6 hpi :Scrambled Control vs. 12 hpi :Biotin Control	0.0761
6 hpi :Scrambled Control vs. 12 hpi :No FISH Control	0.4089
6 hpi :Scrambled Control vs. 12 hpi :Mock Control	<0.0001
6 hpi :Scrambled Control vs. 18 hpi:NS1 mRNA-L	<0.0001
6 hpi :Scrambled Control vs. 18 hpi:No Primary Control	>0.9999
6 hpi :Scrambled Control vs. 18 hpi:No V5 Control	>0.9999
6 hpi :Scrambled Control vs. 18 hpi:Scrambled Control	0.7223
6 hpi :Scrambled Control vs. 18 hpi:Biotin Control	0.8122
6 hpi :Scrambled Control vs. 18 hpi:No FISH Control	>0.9999
6 hpi :Scrambled Control vs. 18 hpi:Mock Control	0.0002
6 hpi :Scrambled Control vs. 24 hpi :NS1 mRNA-L	<0.0001
6 hpi :Scrambled Control vs. 24 hpi :No Primary Control	>0.9999
6 hpi :Scrambled Control vs. 24 hpi :No V5 Control	>0.9999
6 hpi :Scrambled Control vs. 24 hpi :Scrambled Control	0.8122
6 hpi :Scrambled Control vs. 24 hpi :Biotin Control	0.117
6 hpi :Scrambled Control vs. 24 hpi :No FISH Control	>0.9999
6 hpi :Scrambled Control vs. 24 hpi :Mock Control	0.9124
6 hpi :Biotin Control vs. 6 hpi :No FISH Control	>0.9999
6 hpi :Biotin Control vs. 6 hpi :Mock Control	0.2722
6 hpi :Biotin Control vs. 8 hpi :NS1 mRNA-L	<0.0001
6 hpi :Biotin Control vs. 8 hpi :No Primary Control	>0.9999
6 hpi :Biotin Control vs. 8 hpi :No V5 Control	>0.9999
6 hpi :Biotin Control vs. 8 hpi :Scrambled Control	>0.9999
6 hpi :Biotin Control vs. 8 hpi :Biotin Control	>0.9999
6 hpi :Biotin Control vs. 8 hpi :No FISH Control	>0.9999
6 hpi :Biotin Control vs. 8 hpi :Mock Control	0.0034
6 hpi :Biotin Control vs. 12 hpi :NS1 mRNA-L	<0.0001
6 hpi :Biotin Control vs. 12 hpi :No Primary Control	>0.9999
6 hpi :Biotin Control vs. 12 hpi :No V5 Control	0.8841
6 hpi :Biotin Control vs. 12 hpi :Scrambled Control	0.4089
6 hpi :Biotin Control vs. 12 hpi :Biotin Control	0.1353
6 hpi :Biotin Control vs. 12 hpi :No FISH Control	0.5664
6 hpi :Biotin Control vs. 12 hpi :Mock Control	<0.0001
6 hpi :Biotin Control vs. 18 hpi:NS1 mRNA-L	<0.0001
6 hpi :Biotin Control vs. 18 hpi:No Primary Control	>0.9999
6 hpi :Biotin Control vs. 18 hpi:No V5 Control	>0.9999
6 hpi :Biotin Control vs. 18 hpi:Scrambled Control	0.8507
6 hpi :Biotin Control vs. 18 hpi:Biotin Control	0.9124
6 hpi :Biotin Control vs. 18 hpi:No FISH Control	>0.9999
6 hpi :Biotin Control vs. 18 hpi:Mock Control	0.0006
6 hpi :Biotin Control vs. 24 hpi :NS1 mRNA-L	<0.0001
6 hpi :Biotin Control vs. 24 hpi :No Primary Control	>0.9999
6 hpi :Biotin Control vs. 24 hpi :No V5 Control	>0.9999
6 hpi :Biotin Control vs. 24 hpi :Scrambled Control	0.9124
6 hpi :Biotin Control vs. 24 hpi :Biotin Control	0.1989
6 hpi :Biotin Control vs. 24 hpi :No FISH Control	>0.9999
6 hpi :Biotin Control vs. 24 hpi :Mock Control	0.9684

6 hpi :No FISH Control vs. 6 hpi :Mock Control	0.0014
6 hpi :No FISH Control vs. 8 hpi :NS1 mRNA-L	<0.0001
6 hpi :No FISH Control vs. 8 hpi :No Primary Control	>0.9999
6 hpi :No FISH Control vs. 8 hpi :No V5 Control	0.9998
6 hpi :No FISH Control vs. 8 hpi :Scrambled Control	>0.9999
6 hpi :No FISH Control vs. 8 hpi :Biotin Control	0.5664
6 hpi :No FISH Control vs. 8 hpi :No FISH Control	>0.9999
6 hpi :No FISH Control vs. 8 hpi :Mock Control	<0.0001
6 hpi :No FISH Control vs. 12 hpi :NS1 mRNA-L	<0.0001
6 hpi :No FISH Control vs. 12 hpi :No Primary Control	>0.9999
6 hpi :No FISH Control vs. 12 hpi :No V5 Control	0.0418
6 hpi :No FISH Control vs. 12 hpi :Scrambled Control	0.0034
6 hpi :No FISH Control vs. 12 hpi :Biotin Control	0.0004
6 hpi :No FISH Control vs. 12 hpi :No FISH Control	0.0076
6 hpi :No FISH Control vs. 12 hpi :Mock Control	<0.0001
6 hpi :No FISH Control vs. 18 hpi:NS1 mRNA-L	<0.0001
6 hpi :No FISH Control vs. 18 hpi:No Primary Control	>0.9999
6 hpi :No FISH Control vs. 18 hpi:No V5 Control	>0.9999
6 hpi :No FISH Control vs. 18 hpi:Scrambled Control	0.0334
6 hpi :No FISH Control vs. 18 hpi:Biotin Control	0.0521
6 hpi :No FISH Control vs. 18 hpi:No FISH Control	>0.9999
6 hpi :No FISH Control vs. 18 hpi:Mock Control	<0.0001
6 hpi :No FISH Control vs. 24 hpi :NS1 mRNA-L	<0.0001
6 hpi :No FISH Control vs. 24 hpi :No Primary Control	0.979
6 hpi :No FISH Control vs. 24 hpi :No V5 Control	0.9866
6 hpi :No FISH Control vs. 24 hpi :Scrambled Control	0.0521
6 hpi :No FISH Control vs. 24 hpi :Biotin Control	0.0008
6 hpi :No FISH Control vs. 24 hpi :No FISH Control	0.9917
6 hpi :No FISH Control vs. 24 hpi :Mock Control	0.0966
6 hpi :Mock Control vs. 8 hpi :NS1 mRNA-L	0.2336
6 hpi :Mock Control vs. 8 hpi :No Primary Control	0.2722
6 hpi :Mock Control vs. 8 hpi :No V5 Control	0.3602
6 hpi :Mock Control vs. 8 hpi :Scrambled Control	0.0334
6 hpi :Mock Control vs. 8 hpi :Biotin Control	0.9973
6 hpi :Mock Control vs. 8 hpi :No FISH Control	0.117
6 hpi :Mock Control vs. 8 hpi :Mock Control	>0.9999
6 hpi :Mock Control vs. 12 hpi :NS1 mRNA-L	<0.0001
6 hpi :Mock Control vs. 12 hpi :No Primary Control	0.0209
6 hpi :Mock Control vs. 12 hpi :No V5 Control	>0.9999
6 hpi :Mock Control vs. 12 hpi :Scrambled Control	>0.9999
6 hpi :Mock Control vs. 12 hpi :Biotin Control	>0.9999
6 hpi :Mock Control vs. 12 hpi :No FISH Control	>0.9999
6 hpi :Mock Control vs. 12 hpi :Mock Control	<0.0001
6 hpi :Mock Control vs. 18 hpi:NS1 mRNA-L	0.0334
6 hpi :Mock Control vs. 18 hpi:No Primary Control	0.0791
6 hpi :Mock Control vs. 18 hpi:No V5 Control	0.117
6 hpi :Mock Control vs. 18 hpi:Scrambled Control	>0.9999
6 hpi :Mock Control vs. 18 hpi:Biotin Control	>0.9999
6 hpi :Mock Control vs. 18 hpi:No FISH Control	0.1989
6 hpi :Mock Control vs. 18 hpi:Mock Control	0.9996
6 hpi :Mock Control vs. 24 hpi :NS1 mRNA-L	<0.0001
6 hpi :Mock Control vs. 24 hpi :No Primary Control	0.7692
6 hpi :Mock Control vs. 24 hpi :No V5 Control	0.7223
6 hpi :Mock Control vs. 24 hpi :Scrambled Control	>0.9999

6 hpi :Mock Control vs. 24 hpi :Biotin Control	>0.9999
6 hpi :Mock Control vs. 24 hpi :No FISH Control	0.6722
6 hpi :Mock Control vs. 24 hpi :Mock Control	>0.9999
8 hpi :NS1 mRNA-L vs. 8 hpi :No Primary Control	<0.0001
8 hpi :NS1 mRNA-L vs. 8 hpi :No V5 Control	<0.0001
8 hpi :NS1 mRNA-L vs. 8 hpi :Scrambled Control	<0.0001
8 hpi :NS1 mRNA-L vs. 8 hpi :Biotin Control	0.0002
8 hpi :NS1 mRNA-L vs. 8 hpi :No FISH Control	<0.0001
8 hpi :NS1 mRNA-L vs. 8 hpi :Mock Control	0.9866
8 hpi :NS1 mRNA-L vs. 12 hpi :NS1 mRNA-L	<0.0001
8 hpi :NS1 mRNA-L vs. 12 hpi :No Primary Control	<0.0001
8 hpi :NS1 mRNA-L vs. 12 hpi :No V5 Control	0.0163
8 hpi :NS1 mRNA-L vs. 12 hpi :Scrambled Control	0.1407
8 hpi :NS1 mRNA-L vs. 12 hpi :Biotin Control	0.4648
8 hpi :NS1 mRNA-L vs. 12 hpi :No FISH Control	0.0791
8 hpi :NS1 mRNA-L vs. 12 hpi :Mock Control	0.0099
8 hpi :NS1 mRNA-L vs. 18 hpi:NS1 mRNA-L	>0.9999
8 hpi :NS1 mRNA-L vs. 18 hpi:No Primary Control	<0.0001
8 hpi :NS1 mRNA-L vs. 18 hpi:No V5 Control	<0.0001
8 hpi :NS1 mRNA-L vs. 18 hpi:Scrambled Control	0.0209
8 hpi :NS1 mRNA-L vs. 18 hpi:Biotin Control	0.0127
8 hpi :NS1 mRNA-L vs. 18 hpi:No FISH Control	<0.0001
8 hpi :NS1 mRNA-L vs. 18 hpi:Mock Control	0.9996
8 hpi :NS1 mRNA-L vs. 24 hpi :NS1 mRNA-L	<0.0001
8 hpi :NS1 mRNA-L vs. 24 hpi :No Primary Control	<0.0001
8 hpi :NS1 mRNA-L vs. 24 hpi :No V5 Control	<0.0001
8 hpi :NS1 mRNA-L vs. 24 hpi :Scrambled Control	0.0127
8 hpi :NS1 mRNA-L vs. 24 hpi :Biotin Control	0.3145
8 hpi :NS1 mRNA-L vs. 24 hpi :No FISH Control	<0.0001
8 hpi :NS1 mRNA-L vs. 24 hpi :Mock Control	0.0058
8 hpi :No Primary Control vs. 8 hpi :No V5 Control	>0.9999
8 hpi :No Primary Control vs. 8 hpi :Scrambled Control	>0.9999
8 hpi :No Primary Control vs. 8 hpi :Biotin Control	>0.9999
8 hpi :No Primary Control vs. 8 hpi :No FISH Control	>0.9999
8 hpi :No Primary Control vs. 8 hpi :Mock Control	0.0034
8 hpi :No Primary Control vs. 12 hpi :NS1 mRNA-L	<0.0001
8 hpi :No Primary Control vs. 12 hpi :No Primary Control	>0.9999
8 hpi :No Primary Control vs. 12 hpi :No V5 Control	0.8841
8 hpi :No Primary Control vs. 12 hpi :Scrambled Control	0.4089
8 hpi :No Primary Control vs. 12 hpi :Biotin Control	0.1353
8 hpi :No Primary Control vs. 12 hpi :No FISH Control	0.5664
8 hpi :No Primary Control vs. 12 hpi :Mock Control	<0.0001
8 hpi :No Primary Control vs. 18 hpi:NS1 mRNA-L	<0.0001
8 hpi :No Primary Control vs. 18 hpi:No Primary Control	>0.9999
8 hpi :No Primary Control vs. 18 hpi:No V5 Control	>0.9999
8 hpi :No Primary Control vs. 18 hpi:Scrambled Control	0.8507
8 hpi :No Primary Control vs. 18 hpi:Biotin Control	0.9124
8 hpi :No Primary Control vs. 18 hpi:No FISH Control	>0.9999
8 hpi :No Primary Control vs. 18 hpi:Mock Control	0.0006
8 hpi :No Primary Control vs. 24 hpi :NS1 mRNA-L	<0.0001
8 hpi :No Primary Control vs. 24 hpi :No Primary Control	>0.9999
8 hpi :No Primary Control vs. 24 hpi :No V5 Control	>0.9999
8 hpi :No Primary Control vs. 24 hpi :Scrambled Control	0.9124
8 hpi :No Primary Control vs. 24 hpi :Biotin Control	0.1989

8 hpi :No Primary Control vs. 24 hpi :No FISH Control	>0.9999
8 hpi :No Primary Control vs. 24 hpi :Mock Control	0.9684
8 hpi :No V5 Control vs. 8 hpi :Scrambled Control	>0.9999
8 hpi :No V5 Control vs. 8 hpi :Biotin Control	>0.9999
8 hpi :No V5 Control vs. 8 hpi :No FISH Control	>0.9999
8 hpi :No V5 Control vs. 8 hpi :Mock Control	0.0058
8 hpi :No V5 Control vs. 12 hpi :NS1 mRNA-L	<0.0001
8 hpi :No V5 Control vs. 12 hpi :No Primary Control	>0.9999
8 hpi :No V5 Control vs. 12 hpi :No V5 Control	0.9357
8 hpi :No V5 Control vs. 12 hpi :Scrambled Control	0.5128
8 hpi :No V5 Control vs. 12 hpi :Biotin Control	0.1913
8 hpi :No V5 Control vs. 12 hpi :No FISH Control	0.6722
8 hpi :No V5 Control vs. 12 hpi :Mock Control	<0.0001
8 hpi :No V5 Control vs. 18 hpi:NS1 mRNA-L	<0.0001
8 hpi :No V5 Control vs. 18 hpi:No Primary Control	>0.9999
8 hpi :No V5 Control vs. 18 hpi:No V5 Control	>0.9999
8 hpi :No V5 Control vs. 18 hpi:Scrambled Control	0.9124
8 hpi :No V5 Control vs. 18 hpi:Biotin Control	0.9542
8 hpi :No V5 Control vs. 18 hpi:No FISH Control	>0.9999
8 hpi :No V5 Control vs. 18 hpi:Mock Control	0.0011
8 hpi :No V5 Control vs. 24 hpi :NS1 mRNA-L	<0.0001
8 hpi :No V5 Control vs. 24 hpi :No Primary Control	>0.9999
8 hpi :No V5 Control vs. 24 hpi :No V5 Control	>0.9999
8 hpi :No V5 Control vs. 24 hpi :Scrambled Control	0.9542
8 hpi :No V5 Control vs. 24 hpi :Biotin Control	0.2722
8 hpi :No V5 Control vs. 24 hpi :No FISH Control	>0.9999
8 hpi :No V5 Control vs. 24 hpi :Mock Control	0.9866
8 hpi :Scrambled Control vs. 8 hpi :Biotin Control	0.979
8 hpi :Scrambled Control vs. 8 hpi :No FISH Control	>0.9999
8 hpi :Scrambled Control vs. 8 hpi :Mock Control	0.0001
8 hpi :Scrambled Control vs. 12 hpi :NS1 mRNA-L	<0.0001
8 hpi :Scrambled Control vs. 12 hpi :No Primary Control	>0.9999
8 hpi :Scrambled Control vs. 12 hpi :No V5 Control	0.3602
8 hpi :Scrambled Control vs. 12 hpi :Scrambled Control	0.0644
8 hpi :Scrambled Control vs. 12 hpi :Biotin Control	0.0124
8 hpi :Scrambled Control vs. 12 hpi :No FISH Control	0.117
8 hpi :Scrambled Control vs. 12 hpi :Mock Control	<0.0001
8 hpi :Scrambled Control vs. 18 hpi:NS1 mRNA-L	<0.0001
8 hpi :Scrambled Control vs. 18 hpi:No Primary Control	>0.9999
8 hpi :Scrambled Control vs. 18 hpi:No V5 Control	>0.9999
8 hpi :Scrambled Control vs. 18 hpi:Scrambled Control	0.3145
8 hpi :Scrambled Control vs. 18 hpi:Biotin Control	0.4089
8 hpi :Scrambled Control vs. 18 hpi:No FISH Control	>0.9999
8 hpi :Scrambled Control vs. 18 hpi:Mock Control	<0.0001
8 hpi :Scrambled Control vs. 24 hpi :NS1 mRNA-L	<0.0001
8 hpi :Scrambled Control vs. 24 hpi :No Primary Control	>0.9999
8 hpi :Scrambled Control vs. 24 hpi :No V5 Control	>0.9999
8 hpi :Scrambled Control vs. 24 hpi :Scrambled Control	0.4089
8 hpi :Scrambled Control vs. 24 hpi :Biotin Control	0.0209
8 hpi :Scrambled Control vs. 24 hpi :No FISH Control	>0.9999
8 hpi :Scrambled Control vs. 24 hpi :Mock Control	0.5664
8 hpi :Biotin Control vs. 8 hpi :No FISH Control	0.9993
8 hpi :Biotin Control vs. 8 hpi :Mock Control	0.3602
8 hpi :Biotin Control vs. 12 hpi :NS1 mRNA-L	<0.0001

8 hpi :Biotin Control vs. 12 hpi :No Primary Control	0.9542
8 hpi :Biotin Control vs. 12 hpi :No V5 Control	>0.9999
8 hpi :Biotin Control vs. 12 hpi :Scrambled Control	0.9996
8 hpi :Biotin Control vs. 12 hpi :Biotin Control	0.9752
8 hpi :Biotin Control vs. 12 hpi :No FISH Control	>0.9999
8 hpi :Biotin Control vs. 12 hpi :Mock Control	<0.0001
8 hpi :Biotin Control vs. 18 hpi:NS1 mRNA-L	<0.0001
8 hpi :Biotin Control vs. 18 hpi:No Primary Control	0.9973
8 hpi :Biotin Control vs. 18 hpi:No V5 Control	0.9993
8 hpi :Biotin Control vs. 18 hpi:Scrambled Control	>0.9999
8 hpi :Biotin Control vs. 18 hpi:Biotin Control	>0.9999
8 hpi :Biotin Control vs. 18 hpi:No FISH Control	>0.9999
8 hpi :Biotin Control vs. 18 hpi:Mock Control	0.1407
8 hpi :Biotin Control vs. 24 hpi :NS1 mRNA-L	<0.0001
8 hpi :Biotin Control vs. 24 hpi :No Primary Control	>0.9999
8 hpi :Biotin Control vs. 24 hpi :No V5 Control	>0.9999
8 hpi :Biotin Control vs. 24 hpi :Scrambled Control	>0.9999
8 hpi :Biotin Control vs. 24 hpi :Biotin Control	0.9917
8 hpi :Biotin Control vs. 24 hpi :No FISH Control	>0.9999
8 hpi :Biotin Control vs. 24 hpi :Mock Control	>0.9999
8 hpi :No FISH Control vs. 8 hpi :Mock Control	0.0008
8 hpi :No FISH Control vs. 12 hpi :NS1 mRNA-L	<0.0001
8 hpi :No FISH Control vs. 12 hpi :No Primary Control	>0.9999
8 hpi :No FISH Control vs. 12 hpi :No V5 Control	0.6722
8 hpi :No FISH Control vs. 12 hpi :Scrambled Control	0.1989
8 hpi :No FISH Control vs. 12 hpi :Biotin Control	0.0501
8 hpi :No FISH Control vs. 12 hpi :No FISH Control	0.3145
8 hpi :No FISH Control vs. 12 hpi :Mock Control	<0.0001
8 hpi :No FISH Control vs. 18 hpi:NS1 mRNA-L	<0.0001
8 hpi :No FISH Control vs. 18 hpi:No Primary Control	>0.9999
8 hpi :No FISH Control vs. 18 hpi:No V5 Control	>0.9999
8 hpi :No FISH Control vs. 18 hpi:Scrambled Control	0.6199
8 hpi :No FISH Control vs. 18 hpi:Biotin Control	0.7223
8 hpi :No FISH Control vs. 18 hpi:No FISH Control	>0.9999
8 hpi :No FISH Control vs. 18 hpi:Mock Control	0.0001
8 hpi :No FISH Control vs. 24 hpi :NS1 mRNA-L	<0.0001
8 hpi :No FISH Control vs. 24 hpi :No Primary Control	>0.9999
8 hpi :No FISH Control vs. 24 hpi :No V5 Control	>0.9999
8 hpi :No FISH Control vs. 24 hpi :Scrambled Control	0.7223
8 hpi :No FISH Control vs. 24 hpi :Biotin Control	0.0791
8 hpi :No FISH Control vs. 24 hpi :No FISH Control	>0.9999
8 hpi :No FISH Control vs. 24 hpi :Mock Control	0.8507
8 hpi :Mock Control vs. 12 hpi :NS1 mRNA-L	<0.0001
8 hpi :Mock Control vs. 12 hpi :No Primary Control	<0.0001
8 hpi :Mock Control vs. 12 hpi :No V5 Control	0.979
8 hpi :Mock Control vs. 12 hpi :Scrambled Control	>0.9999
8 hpi :Mock Control vs. 12 hpi :Biotin Control	>0.9999
8 hpi :Mock Control vs. 12 hpi :No FISH Control	0.9996
8 hpi :Mock Control vs. 12 hpi :Mock Control	<0.0001
8 hpi :Mock Control vs. 18 hpi:NS1 mRNA-L	0.7223
8 hpi :Mock Control vs. 18 hpi:No Primary Control	0.0004
8 hpi :Mock Control vs. 18 hpi:No V5 Control	0.0008
8 hpi :Mock Control vs. 18 hpi:Scrambled Control	0.9866
8 hpi :Mock Control vs. 18 hpi:Biotin Control	0.9684

8 hpi :Mock Control vs. 18 hpi:No FISH Control	0.0019
8 hpi :Mock Control vs. 18 hpi:Mock Control	>0.9999
8 hpi :Mock Control vs. 24 hpi :NS1 mRNA-L	<0.0001
8 hpi :Mock Control vs. 24 hpi :No Primary Control	0.0418
8 hpi :Mock Control vs. 24 hpi :No V5 Control	0.0334
8 hpi :Mock Control vs. 24 hpi :Scrambled Control	0.9684
8 hpi :Mock Control vs. 24 hpi :Biotin Control	>0.9999
8 hpi :Mock Control vs. 24 hpi :No FISH Control	0.0265
8 hpi :Mock Control vs. 24 hpi :Mock Control	0.9124
12 hpi :NS1 mRNA-L vs. 12 hpi :No Primary Control	<0.0001
12 hpi :NS1 mRNA-L vs. 12 hpi :No V5 Control	<0.0001
12 hpi :NS1 mRNA-L vs. 12 hpi :Scrambled Control	<0.0001
12 hpi :NS1 mRNA-L vs. 12 hpi :Biotin Control	<0.0001
12 hpi :NS1 mRNA-L vs. 12 hpi :No FISH Control	<0.0001
12 hpi :NS1 mRNA-L vs. 12 hpi :Mock Control	<0.0001
12 hpi :NS1 mRNA-L vs. 18 hpi:NS1 mRNA-L	<0.0001
12 hpi :NS1 mRNA-L vs. 18 hpi:No Primary Control	<0.0001
12 hpi :NS1 mRNA-L vs. 18 hpi:No V5 Control	<0.0001
12 hpi :NS1 mRNA-L vs. 18 hpi:Scrambled Control	<0.0001
12 hpi :NS1 mRNA-L vs. 18 hpi:Biotin Control	<0.0001
12 hpi :NS1 mRNA-L vs. 18 hpi:No FISH Control	<0.0001
12 hpi :NS1 mRNA-L vs. 18 hpi:Mock Control	<0.0001
12 hpi :NS1 mRNA-L vs. 24 hpi :NS1 mRNA-L	0.6722
12 hpi :NS1 mRNA-L vs. 24 hpi :No Primary Control	<0.0001
12 hpi :NS1 mRNA-L vs. 24 hpi :No V5 Control	<0.0001
12 hpi :NS1 mRNA-L vs. 24 hpi :Scrambled Control	<0.0001
12 hpi :NS1 mRNA-L vs. 24 hpi :Biotin Control	<0.0001
12 hpi :NS1 mRNA-L vs. 24 hpi :No FISH Control	<0.0001
12 hpi :NS1 mRNA-L vs. 24 hpi :Mock Control	<0.0001
12 hpi :No Primary Control vs. 12 hpi :No V5 Control	0.2722
12 hpi :No Primary Control vs. 12 hpi :Scrambled Control	0.0418
12 hpi :No Primary Control vs. 12 hpi :Biotin Control	0.0074
12 hpi :No Primary Control vs. 12 hpi :No FISH Control	0.0791
12 hpi :No Primary Control vs. 12 hpi :Mock Control	<0.0001
12 hpi :No Primary Control vs. 18 hpi:NS1 mRNA-L	<0.0001
12 hpi :No Primary Control vs. 18 hpi:No Primary Control	>0.9999
12 hpi :No Primary Control vs. 18 hpi:No V5 Control	>0.9999
12 hpi :No Primary Control vs. 18 hpi:Scrambled Control	0.2336
12 hpi :No Primary Control vs. 18 hpi:Biotin Control	0.3145
12 hpi :No Primary Control vs. 18 hpi:No FISH Control	>0.9999
12 hpi :No Primary Control vs. 18 hpi:Mock Control	<0.0001
12 hpi :No Primary Control vs. 24 hpi :NS1 mRNA-L	<0.0001
12 hpi :No Primary Control vs. 24 hpi :No Primary Control	>0.9999
12 hpi :No Primary Control vs. 24 hpi :No V5 Control	>0.9999
12 hpi :No Primary Control vs. 24 hpi :Scrambled Control	0.3145
12 hpi :No Primary Control vs. 24 hpi :Biotin Control	0.0127
12 hpi :No Primary Control vs. 24 hpi :No FISH Control	>0.9999
12 hpi :No Primary Control vs. 24 hpi :Mock Control	0.46
12 hpi :No V5 Control vs. 12 hpi :Scrambled Control	>0.9999
12 hpi :No V5 Control vs. 12 hpi :Biotin Control	>0.9999
12 hpi :No V5 Control vs. 12 hpi :No FISH Control	>0.9999
12 hpi :No V5 Control vs. 12 hpi :Mock Control	<0.0001
12 hpi :No V5 Control vs. 18 hpi:NS1 mRNA-L	0.0011
12 hpi :No V5 Control vs. 18 hpi:No Primary Control	0.5664

12 hpi :No V5 Control vs. 18 hpi:No V5 Control	0.6722
12 hpi :No V5 Control vs. 18 hpi:Scrambled Control	>0.9999
12 hpi :No V5 Control vs. 18 hpi:Biotin Control	>0.9999
12 hpi :No V5 Control vs. 18 hpi:No FISH Control	0.8122
12 hpi :No V5 Control vs. 18 hpi:Mock Control	0.8507
12 hpi :No V5 Control vs. 24 hpi :NS1 mRNA-L	<0.0001
12 hpi :No V5 Control vs. 24 hpi :No Primary Control	0.9985
12 hpi :No V5 Control vs. 24 hpi :No V5 Control	0.9973
12 hpi :No V5 Control vs. 24 hpi :Scrambled Control	>0.9999
12 hpi :No V5 Control vs. 24 hpi :Biotin Control	>0.9999
12 hpi :No V5 Control vs. 24 hpi :No FISH Control	0.9951
12 hpi :No V5 Control vs. 24 hpi :Mock Control	>0.9999
12 hpi :Scrambled Control vs. 12 hpi :Biotin Control	>0.9999
12 hpi :Scrambled Control vs. 12 hpi :No FISH Control	>0.9999
12 hpi :Scrambled Control vs. 12 hpi :Mock Control	<0.0001
12 hpi :Scrambled Control vs. 18 hpi:NS1 mRNA-L	0.0163
12 hpi :Scrambled Control vs. 18 hpi:No Primary Control	0.1407
12 hpi :Scrambled Control vs. 18 hpi:No V5 Control	0.1989
12 hpi :Scrambled Control vs. 18 hpi:Scrambled Control	>0.9999
12 hpi :Scrambled Control vs. 18 hpi:Biotin Control	>0.9999
12 hpi :Scrambled Control vs. 18 hpi:No FISH Control	0.3145
12 hpi :Scrambled Control vs. 18 hpi:Mock Control	0.9973
12 hpi :Scrambled Control vs. 24 hpi :NS1 mRNA-L	<0.0001
12 hpi :Scrambled Control vs. 24 hpi :No Primary Control	0.8841
12 hpi :Scrambled Control vs. 24 hpi :No V5 Control	0.8507
12 hpi :Scrambled Control vs. 24 hpi :Scrambled Control	>0.9999
12 hpi :Scrambled Control vs. 24 hpi :Biotin Control	>0.9999
12 hpi :Scrambled Control vs. 24 hpi :No FISH Control	0.8122
12 hpi :Scrambled Control vs. 24 hpi :Mock Control	>0.9999
12 hpi :Biotin Control vs. 12 hpi :No FISH Control	>0.9999
12 hpi :Biotin Control vs. 12 hpi :Mock Control	<0.0001
12 hpi :Biotin Control vs. 18 hpi:NS1 mRNA-L	0.1
12 hpi :Biotin Control vs. 18 hpi:No Primary Control	0.0322
12 hpi :Biotin Control vs. 18 hpi:No V5 Control	0.0501
12 hpi :Biotin Control vs. 18 hpi:Scrambled Control	>0.9999
12 hpi :Biotin Control vs. 18 hpi:Biotin Control	>0.9999
12 hpi :Biotin Control vs. 18 hpi:No FISH Control	0.0929
12 hpi :Biotin Control vs. 18 hpi:Mock Control	>0.9999
12 hpi :Biotin Control vs. 24 hpi :NS1 mRNA-L	<0.0001
12 hpi :Biotin Control vs. 24 hpi :No Primary Control	0.5503
12 hpi :Biotin Control vs. 24 hpi :No V5 Control	0.4973
12 hpi :Biotin Control vs. 24 hpi :Scrambled Control	>0.9999
12 hpi :Biotin Control vs. 24 hpi :Biotin Control	>0.9999
12 hpi :Biotin Control vs. 24 hpi :No FISH Control	0.4453
12 hpi :Biotin Control vs. 24 hpi :Mock Control	>0.9999
12 hpi :No FISH Control vs. 12 hpi :Mock Control	<0.0001
12 hpi :No FISH Control vs. 18 hpi:NS1 mRNA-L	0.0076
12 hpi :No FISH Control vs. 18 hpi:No Primary Control	0.2336
12 hpi :No FISH Control vs. 18 hpi:No V5 Control	0.3145
12 hpi :No FISH Control vs. 18 hpi:Scrambled Control	>0.9999
12 hpi :No FISH Control vs. 18 hpi:Biotin Control	>0.9999
12 hpi :No FISH Control vs. 18 hpi:No FISH Control	0.46
12 hpi :No FISH Control vs. 18 hpi:Mock Control	0.9866
12 hpi :No FISH Control vs. 24 hpi :NS1 mRNA-L	<0.0001

12 hpi :No FISH Control vs. 24 hpi :No Primary Control	0.9542
12 hpi :No FISH Control vs. 24 hpi :No V5 Control	0.9357
12 hpi :No FISH Control vs. 24 hpi :Scrambled Control	>0.9999
12 hpi :No FISH Control vs. 24 hpi :Biotin Control	>0.9999
12 hpi :No FISH Control vs. 24 hpi :No FISH Control	0.9124
12 hpi :No FISH Control vs. 24 hpi :Mock Control	>0.9999
12 hpi :Mock Control vs. 18 hpi:NS1 mRNA-L	0.0966
12 hpi :Mock Control vs. 18 hpi:No Primary Control	<0.0001
12 hpi :Mock Control vs. 18 hpi:No V5 Control	<0.0001
12 hpi :Mock Control vs. 18 hpi:Scrambled Control	<0.0001
12 hpi :Mock Control vs. 18 hpi:Biotin Control	<0.0001
12 hpi :Mock Control vs. 18 hpi:No FISH Control	<0.0001
12 hpi :Mock Control vs. 18 hpi:Mock Control	<0.0001
12 hpi :Mock Control vs. 24 hpi :NS1 mRNA-L	0.3602
12 hpi :Mock Control vs. 24 hpi :No Primary Control	<0.0001
12 hpi :Mock Control vs. 24 hpi :No V5 Control	<0.0001
12 hpi :Mock Control vs. 24 hpi :Scrambled Control	<0.0001
12 hpi :Mock Control vs. 24 hpi :Biotin Control	<0.0001
12 hpi :Mock Control vs. 24 hpi :No FISH Control	<0.0001
12 hpi :Mock Control vs. 24 hpi :Mock Control	<0.0001
18 hpi:NS1 mRNA-L vs. 18 hpi:No Primary Control	<0.0001
18 hpi:NS1 mRNA-L vs. 18 hpi:No V5 Control	<0.0001
18 hpi:NS1 mRNA-L vs. 18 hpi:Scrambled Control	0.0014
18 hpi:NS1 mRNA-L vs. 18 hpi:Biotin Control	0.0008
18 hpi:NS1 mRNA-L vs. 18 hpi:No FISH Control	<0.0001
18 hpi:NS1 mRNA-L vs. 18 hpi:Mock Control	0.9357
18 hpi:NS1 mRNA-L vs. 24 hpi :NS1 mRNA-L	<0.0001
18 hpi:NS1 mRNA-L vs. 24 hpi :No Primary Control	<0.0001
18 hpi:NS1 mRNA-L vs. 24 hpi :No V5 Control	<0.0001
18 hpi:NS1 mRNA-L vs. 24 hpi :Scrambled Control	0.0008
18 hpi:NS1 mRNA-L vs. 24 hpi :Biotin Control	0.0521
18 hpi:NS1 mRNA-L vs. 24 hpi :No FISH Control	<0.0001
18 hpi:NS1 mRNA-L vs. 24 hpi :Mock Control	0.0003
18 hpi:No Primary Control vs. 18 hpi:No V5 Control	>0.9999
18 hpi:No Primary Control vs. 18 hpi:Scrambled Control	0.5128
18 hpi:No Primary Control vs. 18 hpi:Biotin Control	0.6199
18 hpi:No Primary Control vs. 18 hpi:No FISH Control	>0.9999
18 hpi:No Primary Control vs. 18 hpi:Mock Control	<0.0001
18 hpi:No Primary Control vs. 24 hpi :NS1 mRNA-L	<0.0001
18 hpi:No Primary Control vs. 24 hpi :No Primary Control	>0.9999
18 hpi:No Primary Control vs. 24 hpi :No V5 Control	>0.9999
18 hpi:No Primary Control vs. 24 hpi :Scrambled Control	0.6199
18 hpi:No Primary Control vs. 24 hpi :Biotin Control	0.0521
18 hpi:No Primary Control vs. 24 hpi :No FISH Control	>0.9999
18 hpi:No Primary Control vs. 24 hpi :Mock Control	0.7692
18 hpi:No V5 Control vs. 18 hpi:Scrambled Control	0.6199
18 hpi:No V5 Control vs. 18 hpi:Biotin Control	0.7223
18 hpi:No V5 Control vs. 18 hpi:No FISH Control	>0.9999
18 hpi:No V5 Control vs. 18 hpi:Mock Control	0.0001
18 hpi:No V5 Control vs. 24 hpi :NS1 mRNA-L	<0.0001
18 hpi:No V5 Control vs. 24 hpi :No Primary Control	>0.9999
18 hpi:No V5 Control vs. 24 hpi :No V5 Control	>0.9999
18 hpi:No V5 Control vs. 24 hpi :Scrambled Control	0.7223
18 hpi:No V5 Control vs. 24 hpi :Biotin Control	0.0791

18 hpi:No V5 Control vs. 24 hpi :No FISH Control	>0.9999
18 hpi:No V5 Control vs. 24 hpi :Mock Control	0.8507
18 hpi:Scrambled Control vs. 18 hpi:Biotin Control	>0.9999
18 hpi:Scrambled Control vs. 18 hpi:No FISH Control	0.7692
18 hpi:Scrambled Control vs. 18 hpi:Mock Control	0.8841
18 hpi:Scrambled Control vs. 24 hpi :NS1 mRNA-L	<0.0001
18 hpi:Scrambled Control vs. 24 hpi :No Primary Control	0.9973
18 hpi:Scrambled Control vs. 24 hpi :No V5 Control	0.9951
18 hpi:Scrambled Control vs. 24 hpi :Scrambled Control	>0.9999
18 hpi:Scrambled Control vs. 24 hpi :Biotin Control	>0.9999
18 hpi:Scrambled Control vs. 24 hpi :No FISH Control	0.9917
18 hpi:Scrambled Control vs. 24 hpi :Mock Control	>0.9999
18 hpi:Biotin Control vs. 18 hpi:No FISH Control	0.8507
18 hpi:Biotin Control vs. 18 hpi:Mock Control	0.8122
18 hpi:Biotin Control vs. 24 hpi :NS1 mRNA-L	<0.0001
18 hpi:Biotin Control vs. 24 hpi :No Primary Control	0.9993
18 hpi:Biotin Control vs. 24 hpi :No V5 Control	0.9985
18 hpi:Biotin Control vs. 24 hpi :Scrambled Control	>0.9999
18 hpi:Biotin Control vs. 24 hpi :Biotin Control	>0.9999
18 hpi:Biotin Control vs. 24 hpi :No FISH Control	0.9973
18 hpi:Biotin Control vs. 24 hpi :Mock Control	>0.9999
18 hpi:No FISH Control vs. 18 hpi:Mock Control	0.0003
18 hpi:No FISH Control vs. 24 hpi :NS1 mRNA-L	<0.0001
18 hpi:No FISH Control vs. 24 hpi :No Primary Control	>0.9999
18 hpi:No FISH Control vs. 24 hpi :No V5 Control	>0.9999
18 hpi:No FISH Control vs. 24 hpi :Scrambled Control	0.8507
18 hpi:No FISH Control vs. 24 hpi :Biotin Control	0.1407
18 hpi:No FISH Control vs. 24 hpi :No FISH Control	>0.9999
18 hpi:No FISH Control vs. 24 hpi :Mock Control	0.9357
18 hpi:Mock Control vs. 24 hpi :NS1 mRNA-L	<0.0001
18 hpi:Mock Control vs. 24 hpi :No Primary Control	0.0099
18 hpi:Mock Control vs. 24 hpi :No V5 Control	0.0076
18 hpi:Mock Control vs. 24 hpi :Scrambled Control	0.8122
18 hpi:Mock Control vs. 24 hpi :Biotin Control	>0.9999
18 hpi:Mock Control vs. 24 hpi :No FISH Control	0.0058
18 hpi:Mock Control vs. 24 hpi :Mock Control	0.6722
24 hpi :NS1 mRNA-L vs. 24 hpi :No Primary Control	<0.0001
24 hpi :NS1 mRNA-L vs. 24 hpi :No V5 Control	<0.0001
24 hpi :NS1 mRNA-L vs. 24 hpi :Scrambled Control	<0.0001
24 hpi :NS1 mRNA-L vs. 24 hpi :Biotin Control	<0.0001
24 hpi :NS1 mRNA-L vs. 24 hpi :No FISH Control	<0.0001
24 hpi :NS1 mRNA-L vs. 24 hpi :Mock Control	<0.0001
24 hpi :No Primary Control vs. 24 hpi :No V5 Control	>0.9999
24 hpi :No Primary Control vs. 24 hpi :Scrambled Control	0.9993
24 hpi :No Primary Control vs. 24 hpi :Biotin Control	0.6722
24 hpi :No Primary Control vs. 24 hpi :No FISH Control	>0.9999
24 hpi :No Primary Control vs. 24 hpi :Mock Control	>0.9999
24 hpi :No V5 Control vs. 24 hpi :Scrambled Control	0.9985
24 hpi :No V5 Control vs. 24 hpi :Biotin Control	0.6199
24 hpi :No V5 Control vs. 24 hpi :No FISH Control	>0.9999
24 hpi :No V5 Control vs. 24 hpi :Mock Control	0.9998
24 hpi :Scrambled Control vs. 24 hpi :Biotin Control	>0.9999
24 hpi :Scrambled Control vs. 24 hpi :No FISH Control	0.9973
24 hpi :Scrambled Control vs. 24 hpi :Mock Control	>0.9999

24 hpi :Biotin Control vs. 24 hpi :No FISH Control	0.5664
24 hpi :Biotin Control vs. 24 hpi :Mock Control	>0.9999
24 hpi :No FISH Control vs. 24 hpi :Mock Control	0.9996
**Graph Title**	NS1 mRNA-L PLA (Normalized PLA)
Comparison	P Value
6 hpi	
NS1 mRNA-L vs. No Primary Control	<0.0001
NS1 mRNA-L vs. No V5 Control	<0.0001
NS1 mRNA-L vs. Scrambled Control	<0.0001
NS1 mRNA-L vs. Biotin Control	<0.0001
NS1 mRNA-L vs. No FISH Control	<0.0001
No Primary Control vs. No V5 Control	0.7602
No Primary Control vs. Scrambled Control	>0.9999
No Primary Control vs. Biotin Control	0.9952
No Primary Control vs. No FISH Control	0.9557
No V5 Control vs. Scrambled Control	0.7358
No V5 Control vs. Biotin Control	0.9638
No V5 Control vs. No FISH Control	0.2367
Scrambled Control vs. Biotin Control	0.9932
Scrambled Control vs. No FISH Control	0.9641
Biotin Control vs. No FISH Control	0.7368
8 hpi	
NS1 mRNA-L vs. No Primary Control	0.2007
NS1 mRNA-L vs. No V5 Control	0.5055
NS1 mRNA-L vs. Scrambled Control	0.0006
NS1 mRNA-L vs. Biotin Control	0.0362
NS1 mRNA-L vs. No FISH Control	0.0054
No Primary Control vs. No V5 Control	0.9942
No Primary Control vs. Scrambled Control	0.447
No Primary Control vs. Biotin Control	0.9839
No Primary Control vs. No FISH Control	0.8028
No V5 Control vs. Scrambled Control	0.1658
No V5 Control vs. Biotin Control	0.823
No V5 Control vs. No FISH Control	0.4574
Scrambled Control vs. Biotin Control	0.8612
Scrambled Control vs. No FISH Control	0.9934
Biotin Control vs. No FISH Control	0.992
12 hpi	
NS1 mRNA-L vs. No Primary Control	<0.0001
NS1 mRNA-L vs. No V5 Control	0.4994
NS1 mRNA-L vs. Scrambled Control	0.6493
NS1 mRNA-L vs. Biotin Control	0.9568
NS1 mRNA-L vs. No FISH Control	<0.0001
No Primary Control vs. No V5 Control	0.0058
No Primary Control vs. Scrambled Control	0.0025
No Primary Control vs. Biotin Control	0.0002
No Primary Control vs. No FISH Control	0.9955
No V5 Control vs. Scrambled Control	>0.9999
No V5 Control vs. Biotin Control	0.9479
No V5 Control vs. No FISH Control	0.0316
Scrambled Control vs. Biotin Control	0.9853
Scrambled Control vs. No FISH Control	0.0156

Biotin Control vs. No FISH Control	0.0014
18 hpi	
NS1 mRNA-L vs. No Primary Control	0.7854
NS1 mRNA-L vs. No V5 Control	0.8917
NS1 mRNA-L vs. Scrambled Control	0.9206
NS1 mRNA-L vs. Biotin Control	0.8474
NS1 mRNA-L vs. No FISH Control	0.9138
No Primary Control vs. No V5 Control	>0.9999
No Primary Control vs. Scrambled Control	0.9996
No Primary Control vs. Biotin Control	>0.9999
No Primary Control vs. No FISH Control	0.9997
No V5 Control vs. Scrambled Control	>0.9999
No V5 Control vs. Biotin Control	>0.9999
No V5 Control vs. No FISH Control	>0.9999
Scrambled Control vs. Biotin Control	>0.9999
Scrambled Control vs. No FISH Control	>0.9999
Biotin Control vs. No FISH Control	>0.9999
24 hpi	
NS1 mRNA-L vs. No Primary Control	0.5273
NS1 mRNA-L vs. No V5 Control	0.4757
NS1 mRNA-L vs. Scrambled Control	0.9993
NS1 mRNA-L vs. Biotin Control	0.9107
NS1 mRNA-L vs. No FISH Control	0.9649
No Primary Control vs. No V5 Control	>0.9999
No Primary Control vs. Scrambled Control	0.7545
No Primary Control vs. Biotin Control	0.0707
No Primary Control vs. No FISH Control	0.9483
No V5 Control vs. Scrambled Control	0.7073
No V5 Control vs. Biotin Control	0.0572
No V5 Control vs. No FISH Control	0.9271
Scrambled Control vs. Biotin Control	0.7421
Scrambled Control vs. No FISH Control	0.9974
Biotin Control vs. No FISH Control	0.4428

Figure S7D	
**Graph Title**	Poly(A)-L PLA (# of Puncta)
Comparison	P Value
6 hpi	
PolyA-L vs. No Primary Control	0.001
PolyA-L vs. No V5 Control	<0.0001
PolyA-L vs. Scrambled Control	0.0029
PolyA-L vs. Biotin Control	0.0078
PolyA-L vs. No FISH Control	<0.0001
PolyA-L vs. Mock Control	0.061
No Primary Control vs. No V5 Control	0.9922
No Primary Control vs. Scrambled Control	>0.9999
No Primary Control vs. Biotin Control	0.9984
No Primary Control vs. No FISH Control	0.7441
No Primary Control vs. Mock Control	0.9141
No V5 Control vs. Scrambled Control	0.96
No V5 Control vs. Biotin Control	0.8798
No V5 Control vs. No FISH Control	0.9855
No V5 Control vs. Mock Control	0.5178
Scrambled Control vs. Biotin Control	>0.9999

Scrambled Control vs. No FISH Control	0.5713
Scrambled Control vs. Mock Control	0.9753
Biotin Control vs. No FISH Control	0.3954
Biotin Control vs. Mock Control	0.9962
No FISH Control vs. Mock Control	0.1172
8 hpi	
PolyA-L vs. No Primary Control	0.0144
PolyA-L vs. No V5 Control	0.0255
PolyA-L vs. Scrambled Control	0.0144
PolyA-L vs. Biotin Control	0.3418
PolyA-L vs. No FISH Control	0.0714
PolyA-L vs. Mock Control	0.0714
No Primary Control vs. No V5 Control	>0.9999
No Primary Control vs. Scrambled Control	>0.9999
No Primary Control vs. Biotin Control	0.8798
No Primary Control vs. No FISH Control	0.9984
No Primary Control vs. Mock Control	0.9984
No V5 Control vs. Scrambled Control	>0.9999
No V5 Control vs. Biotin Control	0.9395
No V5 Control vs. No FISH Control	0.9998
No V5 Control vs. Mock Control	0.9998
Scrambled Control vs. Biotin Control	0.8798
Scrambled Control vs. No FISH Control	0.9984
Scrambled Control vs. Mock Control	0.9984
Biotin Control vs. No FISH Control	0.9922
Biotin Control vs. Mock Control	0.9922
No FISH Control vs. Mock Control	>0.9999
12 hpi	
PolyA-L vs. No Primary Control	<0.0001
PolyA-L vs. No V5 Control	<0.0001
PolyA-L vs. Scrambled Control	<0.0001
PolyA-L vs. Biotin Control	<0.0001
PolyA-L vs. No FISH Control	<0.0001
PolyA-L vs. Mock Control	<0.0001
No Primary Control vs. No V5 Control	0.9129
No Primary Control vs. Scrambled Control	0.1392
No Primary Control vs. Biotin Control	0.0229
No Primary Control vs. No FISH Control	0.2467
No Primary Control vs. Mock Control	0.0028
No V5 Control vs. Scrambled Control	0.7949
No V5 Control vs. Biotin Control	0.3688
No V5 Control vs. No FISH Control	0.9129
No V5 Control vs. Mock Control	0.105
Scrambled Control vs. Biotin Control	0.994
Scrambled Control vs. No FISH Control	>0.9999
Scrambled Control vs. Mock Control	0.857
Biotin Control vs. No FISH Control	0.9667
Biotin Control vs. Mock Control	0.9963
No FISH Control vs. Mock Control	0.7145
18 hpi	
PolyA-L vs. No Primary Control	<0.0001
PolyA-L vs. No V5 Control	<0.0001
PolyA-L vs. Scrambled Control	0.0274
PolyA-L vs. Biotin Control	0.0435

PolyA-L vs. No FISH Control	<0.0001
PolyA-L vs. Mock Control	<0.0001
No Primary Control vs. No V5 Control	0.9751
No Primary Control vs. Scrambled Control	0.0119
No Primary Control vs. Biotin Control	0.0057
No Primary Control vs. No FISH Control	>0.9999
No Primary Control vs. Mock Control	0.8798
No V5 Control vs. Scrambled Control	0.1475
No V5 Control vs. Biotin Control	0.0901
No V5 Control vs. No FISH Control	0.9962
No V5 Control vs. Mock Control	0.9998
Scrambled Control vs. Biotin Control	>0.9999
Scrambled Control vs. No FISH Control	0.028
Scrambled Control vs. Mock Control	0.3039
Biotin Control vs. No FISH Control	0.0144
Biotin Control vs. Mock Control	0.2061
No FISH Control vs. Mock Control	0.96
24 hpi	
PolyA-L vs. No Primary Control	<0.0001
PolyA-L vs. No V5 Control	<0.0001
PolyA-L vs. Scrambled Control	0.0015
PolyA-L vs. Biotin Control	0.0403
PolyA-L vs. No FISH Control	<0.0001
PolyA-L vs. Mock Control	<0.0001
No Primary Control vs. No V5 Control	0.9962
No Primary Control vs. Scrambled Control	0.0334
No Primary Control vs. Biotin Control	0.0015
No Primary Control vs. No FISH Control	0.9984
No Primary Control vs. Mock Control	0.9855
No V5 Control vs. Scrambled Control	0.1703
No V5 Control vs. Biotin Control	0.014
No V5 Control vs. No FISH Control	>0.9999
No V5 Control vs. Mock Control	>0.9999
Scrambled Control vs. Biotin Control	0.9707
Scrambled Control vs. No FISH Control	0.1392
Scrambled Control vs. Mock Control	0.2467
Biotin Control vs. No FISH Control	0.0104
Biotin Control vs. Mock Control	0.0247
No FISH Control vs. Mock Control	>0.9999
**Graph Title**	Poly(A)-L PLA (Normalized PLA)
Comparison	P Value
6 hpi	
poly(A)-L vs. No Primary Control	0.0011
poly(A)-L vs. No V5 Control	<0.0001
poly(A)-L vs. Scrambled Control	<0.0001
poly(A)-L vs. Biotin Control	0.0002
poly(A)-L vs. No FISH Control	<0.0001
No Primary Control vs. No V5 Control	0.6882
No Primary Control vs. Scrambled Control	0.9406
No Primary Control vs. Biotin Control	0.999
No Primary Control vs. No FISH Control	0.5261
No V5 Control vs. Scrambled Control	0.9943
No V5 Control vs. Biotin Control	0.885
No V5 Control vs. No FISH Control	0.9999

Scrambled Control vs. Biotin Control	0.9942
Scrambled Control vs. No FISH Control	0.9688
Biotin Control vs. No FISH Control	0.7629
8 hpi	
poly(A)-L vs. No Primary Control	0.6621
poly(A)-L vs. No V5 Control	0.6031
poly(A)-L vs. Scrambled Control	0.2549
poly(A)-L vs. Biotin Control	0.8988
poly(A)-L vs. No FISH Control	0.5844
No Primary Control vs. No V5 Control	>0.9999
No Primary Control vs. Scrambled Control	0.9859
No Primary Control vs. Biotin Control	0.9977
No Primary Control vs. No FISH Control	>0.9999
No V5 Control vs. Scrambled Control	0.9928
No V5 Control vs. Biotin Control	0.9946
No V5 Control vs. No FISH Control	>0.9999
Scrambled Control vs. Biotin Control	0.877
Scrambled Control vs. No FISH Control	0.9943
Biotin Control vs. No FISH Control	0.9931
12 hpi	
poly(A)-L vs. No Primary Control	0.5478
poly(A)-L vs. No V5 Control	0.024
poly(A)-L vs. Scrambled Control	0.8256
poly(A)-L vs. Biotin Control	0.4215
poly(A)-L vs. No FISH Control	0.3942
No Primary Control vs. No V5 Control	<0.0001
No Primary Control vs. Scrambled Control	0.0442
No Primary Control vs. Biotin Control	0.0055
No Primary Control vs. No FISH Control	0.9999
No V5 Control vs. Scrambled Control	0.4142
No V5 Control vs. Biotin Control	0.8197
No V5 Control vs. No FISH Control	<0.0001
Scrambled Control vs. Biotin Control	0.9874
Scrambled Control vs. No FISH Control	0.0217
Biotin Control vs. No FISH Control	0.0023
18 hpi	
poly(A)-L vs. No Primary Control	0.9727
poly(A)-L vs. No V5 Control	0.9948
poly(A)-L vs. Scrambled Control	0.9966
poly(A)-L vs. Biotin Control	0.9854
poly(A)-L vs. No FISH Control	0.9959
No Primary Control vs. No V5 Control	>0.9999
No Primary Control vs. Scrambled Control	0.9998
No Primary Control vs. Biotin Control	>0.9999
No Primary Control vs. No FISH Control	0.9998
No V5 Control vs. Scrambled Control	>0.9999
No V5 Control vs. Biotin Control	>0.9999
No V5 Control vs. No FISH Control	>0.9999
Scrambled Control vs. Biotin Control	>0.9999
Scrambled Control vs. No FISH Control	>0.9999
Biotin Control vs. No FISH Control	>0.9999
24 hpi	
poly(A)-L vs. No Primary Control	>0.9999
poly(A)-L vs. No V5 Control	0.9987

poly(A)-L vs. Scrambled Control	0.9249
poly(A)-L vs. Biotin Control	0.1914
poly(A)-L vs. No FISH Control	0.9949
No Primary Control vs. No V5 Control	0.9996
No Primary Control vs. Scrambled Control	0.8932
No Primary Control vs. Biotin Control	0.157
No Primary Control vs. No FISH Control	0.9893
No V5 Control vs. Scrambled Control	0.7412
No V5 Control vs. Biotin Control	0.0748
No V5 Control vs. No FISH Control	0.9388
Scrambled Control vs. Biotin Control	0.7678
Scrambled Control vs. No FISH Control	0.9978
Biotin Control vs. No FISH Control	0.4809

**Figure S7E**	
**Graph Title**	NS1 mRNA vs Poly(A)
Comparison	P Value
6 hpi	>0.9999
8 hpi	0.3876
12 hpi	0.0637
18 hpi	0.9956
24 hpi	0.7162

**Figure S8C**	
**Graph Title**	Plaque Assay
Comparison	P Value
M2-2-Myc mRNA:200 ng vs. M2-2-Myc mRNA:100 ng	0.7709
M2-2-Myc mRNA:200 ng vs. M2-2-Myc mRNA:50 ng	0.1574
M2-2-Myc mRNA:200 ng vs. M2-2-Myc mRNA:0 ng	<0.0001
M2-2-Myc mRNA:200 ng vs. M2-1-HA mRNA:200 ng	<0.0001
M2-2-Myc mRNA:200 ng vs. M2-1-HA mRNA:100 ng	<0.0001
M2-2-Myc mRNA:200 ng vs. M2-1-HA mRNA:50 ng	<0.0001
M2-2-Myc mRNA:200 ng vs. M2-1-HA mRNA:0 ng	<0.0001
M2-2-Myc mRNA:100 ng vs. M2-2-Myc mRNA:50 ng	0.9274
M2-2-Myc mRNA:100 ng vs. M2-2-Myc mRNA:0 ng	0.0012
M2-2-Myc mRNA:100 ng vs. M2-1-HA mRNA:200 ng	0.0017
M2-2-Myc mRNA:100 ng vs. M2-1-HA mRNA:100 ng	0.0001
M2-2-Myc mRNA:100 ng vs. M2-1-HA mRNA:50 ng	<0.0001
M2-2-Myc mRNA:100 ng vs. M2-1-HA mRNA:0 ng	0.0012
M2-2-Myc mRNA:50 ng vs. M2-2-Myc mRNA:0 ng	0.0209
M2-2-Myc mRNA:50 ng vs. M2-1-HA mRNA:200 ng	0.0285
M2-2-Myc mRNA:50 ng vs. M2-1-HA mRNA:100 ng	0.0021
M2-2-Myc mRNA:50 ng vs. M2-1-HA mRNA:50 ng	0.0002
M2-2-Myc mRNA:50 ng vs. M2-1-HA mRNA:0 ng	0.0209
M2-2-Myc mRNA:0 ng vs. M2-1-HA mRNA:200 ng	>0.9999
M2-2-Myc mRNA:0 ng vs. M2-1-HA mRNA:100 ng	0.9759
M2-2-Myc mRNA:0 ng vs. M2-1-HA mRNA:50 ng	0.5227
M2-2-Myc mRNA:0 ng vs. M2-1-HA mRNA:0 ng	>0.9999
M2-1-HA mRNA:200 ng vs. M2-1-HA mRNA:100 ng	0.9512
M2-1-HA mRNA:200 ng vs. M2-1-HA mRNA:50 ng	0.4404
M2-1-HA mRNA:200 ng vs. M2-1-HA mRNA:0 ng	>0.9999
M2-1-HA mRNA:100 ng vs. M2-1-HA mRNA:50 ng	0.969
M2-1-HA mRNA:100 ng vs. M2-1-HA mRNA:0 ng	0.9759

M2-1-HA mRNA:50 ng vs. M2-1-HA mRNA:0 ng	0.5227

Figure S8A	
**Graph Title**	Veros vs A549s dSTORM
Comparison	P Value
Veros:6 hpi vs. Veros:8 hpi	0.0005
Veros:6 hpi vs. A549s:6 hpi	0.759
Veros:6 hpi vs. A549s:8 hpi	<0.0001
Veros:8 hpi vs. A549s:6 hpi	0.3416
Veros:8 hpi vs. A549s:8 hpi	0.1551
A549s:6 hpi vs. A549s:8 hpi	0.0163

Figure S8D	
**Graph Title**	qRT-PCR
Comparison	P Value
6:M2-2 vs. 6:GFP	0.5399
6:M2-2 vs. 6:L2K	0.6113
6:M2-2 vs. 8:M2-2	0.2516
6:M2-2 vs. 8:GFP	0.5944
6:M2-2 vs. 8:L2K	0.5472
6:M2-2 vs. 12:M2-2	0.0942
6:M2-2 vs. 12:GFP	0.0007
6:M2-2 vs. 12:L2K	0.0002
6:GFP vs. 6:L2K	>0.9999
6:GFP vs. 8:M2-2	0.998
6:GFP vs. 8:GFP	>0.9999
6:GFP vs. 8:L2K	>0.9999
6:GFP vs. 12:M2-2	0.0063
6:GFP vs. 12:GFP	<0.0001
6:GFP vs. 12:L2K	<0.0001
6:L2K vs. 8:M2-2	0.9933
6:L2K vs. 8:GFP	>0.9999
6:L2K vs. 8:L2K	>0.9999
6:L2K vs. 12:M2-2	0.0074
6:L2K vs. 12:GFP	0.0001
6:L2K vs. 12:L2K	<0.0001
8:M2-2 vs. 8:GFP	0.9948
8:M2-2 vs. 8:L2K	0.9977
8:M2-2 vs. 12:M2-2	0.0028
8:M2-2 vs. 12:GFP	<0.0001
8:M2-2 vs. 12:L2K	<0.0001
8:GFP vs. 8:L2K	>0.9999
8:GFP vs. 12:M2-2	0.0071
8:GFP vs. 12:GFP	0.0001
8:GFP vs. 12:L2K	<0.0001
8:L2K vs. 12:M2-2	0.0064
8:L2K vs. 12:GFP	<0.0001
8:L2K vs. 12:L2K	<0.0001
12:M2-2 vs. 12:GFP	0.0414
12:M2-2 vs. 12:L2K	0.0077
12:GFP vs. 12:L2K	0.9101

Figure S9C

**Graph Title**	RSV genome-L PLA (# of Puncta)
Comparison	P Value
M2-2 Myc mRNA 6 hpi	
RSVg-L vs. No Primary Control	<0.0001
RSVg-L vs. No V5 Control	0.001
RSVg-L vs. Scrambled Control	0.0039
RSVg-L vs. Biotin Control	0.0055
RSVg-L vs. No FISH Control	0.0071
RSVg-L vs. Mock Infected Control	0.9979
No Primary Control vs. No V5 Control	0.9194
No Primary Control vs. Scrambled Control	<0.0001
No Primary Control vs. Biotin Control	<0.0001
No Primary Control vs. No FISH Control	0.6662
No Primary Control vs. Mock Infected Control	<0.0001
No V5 Control vs. Scrambled Control	<0.0001
No V5 Control vs. Biotin Control	<0.0001
No V5 Control vs. No FISH Control	0.9988
No V5 Control vs. Mock Infected Control	0.0087
Scrambled Control vs. Biotin Control	>0.9999
Scrambled Control vs. No FISH Control	<0.0001
Scrambled Control vs. Mock Infected Control	0.0004
Biotin Control vs. No FISH Control	<0.0001
Biotin Control vs. Mock Infected Control	0.0006
No FISH Control vs. Mock Infected Control	0.0433
M2-2 Myc mRNA 8 hpi	
RSVg-L vs. No Primary Control	<0.0001
RSVg-L vs. No V5 Control	0.6271
RSVg-L vs. Scrambled Control	0.6271
RSVg-L vs. Biotin Control	0.3397
RSVg-L vs. No FISH Control	<0.0001
RSVg-L vs. Mock Infected Control	0.9348
No Primary Control vs. No V5 Control	<0.0001
No Primary Control vs. Scrambled Control	<0.0001
No Primary Control vs. Biotin Control	0.0003
No Primary Control vs. No FISH Control	0.9993
No Primary Control vs. Mock Infected Control	<0.0001
No V5 Control vs. Scrambled Control	>0.9999
No V5 Control vs. Biotin Control	0.0025
No V5 Control vs. No FISH Control	<0.0001
No V5 Control vs. Mock Infected Control	0.9964
Scrambled Control vs. Biotin Control	0.0025
Scrambled Control vs. No FISH Control	<0.0001
Scrambled Control vs. Mock Infected Control	0.9964
Biotin Control vs. No FISH Control	0.002
Biotin Control vs. Mock Infected Control	0.022
No FISH Control vs. Mock Infected Control	<0.0001
M2-1 HA mRNA 6 hpi	
RSVg-L vs. No Primary Control	<0.0001
RSVg-L vs. No V5 Control	<0.0001
RSVg-L vs. Scrambled Control	0.0105
RSVg-L vs. Biotin Control	<0.0001
RSVg-L vs. No FISH Control	<0.0001
RSVg-L vs. Mock Infected Control	<0.0001
No Primary Control vs. No V5 Control	0.6734

No Primary Control vs. Scrambled Control	<0.0001
No Primary Control vs. Biotin Control	0.0071
No Primary Control vs. No FISH Control	0.2275
No Primary Control vs. Mock Infected Control	0.0031
No V5 Control vs. Scrambled Control	<0.0001
No V5 Control vs. Biotin Control	0.4492
No V5 Control vs. No FISH Control	0.9912
No V5 Control vs. Mock Infected Control	0.3128
Scrambled Control vs. Biotin Control	0.022
Scrambled Control vs. No FISH Control	0.0002
Scrambled Control vs. Mock Infected Control	0.0433
Biotin Control vs. No FISH Control	0.8847
Biotin Control vs. Mock Infected Control	>0.9999
No FISH Control vs. Mock Infected Control	0.7759
M2-1 HA mRNA 8 hpi	
RSVg-L vs. No Primary Control	<0.0001
RSVg-L vs. No V5 Control	<0.0001
RSVg-L vs. Scrambled Control	<0.0001
RSVg-L vs. Biotin Control	<0.0001
RSVg-L vs. No FISH Control	<0.0001
RSVg-L vs. Mock Infected Control	<0.0001
No Primary Control vs. No V5 Control	0.9994
No Primary Control vs. Scrambled Control	0.5614
No Primary Control vs. Biotin Control	0.0016
No Primary Control vs. No FISH Control	0.835
No Primary Control vs. Mock Infected Control	<0.0001
No V5 Control vs. Scrambled Control	0.835
No V5 Control vs. Biotin Control	0.0087
No V5 Control vs. No FISH Control	0.974
No V5 Control vs. Mock Infected Control	0.0001
Scrambled Control vs. Biotin Control	0.3128
Scrambled Control vs. No FISH Control	0.9994
Scrambled Control vs. Mock Infected Control	0.022
Biotin Control vs. No FISH Control	0.1225
Biotin Control vs. Mock Infected Control	0.9401
No FISH Control vs. Mock Infected Control	0.0048
**Graph Title**	RSV genome-L PLA (Normalized PLA)
Comparison	P Value
M2-2 Myc mRNA 6 hpi	
RSVg-L vs. No Primary Control	0.1579
RSVg-L vs. No V5 Control	0.2997
RSVg-L vs. Scrambled Control	0.2434
RSVg-L vs. Biotin Control	0.0937
RSVg-L vs. No FISH Control	0.1391
No Primary Control vs. No V5 Control	0.9994
No Primary Control vs. Scrambled Control	<0.0001
No Primary Control vs. Biotin Control	<0.0001
No Primary Control vs. No FISH Control	>0.9999
No V5 Control vs. Scrambled Control	0.0003
No V5 Control vs. Biotin Control	<0.0001
No V5 Control vs. No FISH Control	0.999
Scrambled Control vs. Biotin Control	0.9981
Scrambled Control vs. No FISH Control	<0.0001

Biotin Control vs. No FISH Control	<0.0001
M2-2 Myc mRNA 8 hpi	
RSVg-L vs. No Primary Control	0.0637
RSVg-L vs. No V5 Control	0.83
RSVg-L vs. Scrambled Control	0.4367
RSVg-L vs. Biotin Control	>0.9999
RSVg-L vs. No FISH Control	0.8429
No Primary Control vs. No V5 Control	0.001
No Primary Control vs. Scrambled Control	<0.0001
No Primary Control vs. Biotin Control	0.089
No Primary Control vs. No FISH Control	0.5952
No V5 Control vs. Scrambled Control	0.9881
No V5 Control vs. Biotin Control	0.7406
No V5 Control vs. No FISH Control	0.15
Scrambled Control vs. Biotin Control	0.3347
Scrambled Control vs. No FISH Control	0.0278
Biotin Control vs. No FISH Control	0.9027
M2-1 HA mRNA 6 hpi	
RSVg-L vs. No Primary Control	<0.0001
RSVg-L vs. No V5 Control	0.0091
RSVg-L vs. Scrambled Control	0.007
RSVg-L vs. Biotin Control	0.0358
RSVg-L vs. No FISH Control	0.0251
No Primary Control vs. No V5 Control	0.8535
No Primary Control vs. Scrambled Control	0.8856
No Primary Control vs. Biotin Control	0.6028
No Primary Control vs. No FISH Control	0.68
No V5 Control vs. Scrambled Control	>0.9999
No V5 Control vs. Biotin Control	0.9981
No V5 Control vs. No FISH Control	0.9996
Scrambled Control vs. Biotin Control	0.996
Scrambled Control vs. No FISH Control	0.9989
Biotin Control vs. No FISH Control	>0.9999
M2-1 HA mRNA 8 hpi	
RSVg-L vs. No Primary Control	<0.0001
RSVg-L vs. No V5 Control	0.0007
RSVg-L vs. Scrambled Control	0.008
RSVg-L vs. Biotin Control	0.0139
RSVg-L vs. No FISH Control	<0.0001
No Primary Control vs. No V5 Control	0.9487
No Primary Control vs. Scrambled Control	0.6522
No Primary Control vs. Biotin Control	0.5435
No Primary Control vs. No FISH Control	>0.9999
No V5 Control vs. Scrambled Control	0.9889
No V5 Control vs. Biotin Control	0.9688
No V5 Control vs. No FISH Control	0.9555
Scrambled Control vs. Biotin Control	>0.9999
Scrambled Control vs. No FISH Control	0.6712
Biotin Control vs. No FISH Control	0.5632
**Graph Title**	NS1 mRNA-L PLA (# of Puncta)
Comparison	P Value
M2-2-Myc mRNA 6 hpi	
NS1-L vs. No Primary Control	<0.0001

NS1-L vs. No V5 Control	0.0276
NS1-L vs. Scrambled Control	0.6412
NS1-L vs. Biotin Control	0.0104
NS1-L vs. No FISH Control	0.8615
NS1-L vs. Mock Infected Control	0.9981
No Primary Control vs. No V5 Control	0.5838
No Primary Control vs. Scrambled Control	0.0211
No Primary Control vs. Biotin Control	0.7744
No Primary Control vs. No FISH Control	0.0057
No Primary Control vs. Mock Infected Control	<0.0001
No V5 Control vs. Scrambled Control	0.7496
No V5 Control vs. Biotin Control	>0.9999
No V5 Control vs. No FISH Control	0.497
No V5 Control vs. Mock Infected Control	0.0048
Scrambled Control vs. Biotin Control	0.5548
Scrambled Control vs. No FISH Control	0.9998
Scrambled Control vs. Mock Infected Control	0.3
Biotin Control vs. No FISH Control	0.3107
Biotin Control vs. Mock Infected Control	0.0015
No FISH Control vs. Mock Infected Control	0.5394
M2-2-Myc mRNA 8 hpi	
NS1-L vs. No Primary Control	<0.0001
NS1-L vs. No V5 Control	<0.0001
NS1-L vs. Scrambled Control	<0.0001
NS1-L vs. Biotin Control	0.023
NS1-L vs. No FISH Control	0.3107
NS1-L vs. Mock Infected Control	0.0143
No Primary Control vs. No V5 Control	0.1873
No Primary Control vs. Scrambled Control	0.0358
No Primary Control vs. Biotin Control	<0.0001
No Primary Control vs. No FISH Control	<0.0001
No Primary Control vs. Mock Infected Control	<0.0001
No V5 Control vs. Scrambled Control	0.9955
No V5 Control vs. Biotin Control	<0.0001
No V5 Control vs. No FISH Control	<0.0001
No V5 Control vs. Mock Infected Control	<0.0001
Scrambled Control vs. Biotin Control	0.0013
Scrambled Control vs. No FISH Control	<0.0001
Scrambled Control vs. Mock Infected Control	<0.0001
Biotin Control vs. No FISH Control	<0.0001
Biotin Control vs. Mock Infected Control	<0.0001
No FISH Control vs. Mock Infected Control	0.8849
M2-1-HA mRNA 6 hpi	
NS1-L vs. No Primary Control	<0.0001
NS1-L vs. No V5 Control	<0.0001
NS1-L vs. Scrambled Control	<0.0001
NS1-L vs. Biotin Control	<0.0001
NS1-L vs. No FISH Control	<0.0001
NS1-L vs. Mock Infected Control	<0.0001
No Primary Control vs. No V5 Control	0.9998
No Primary Control vs. Scrambled Control	0.8207
No Primary Control vs. Biotin Control	0.413
No Primary Control vs. No FISH Control	0.0018
No Primary Control vs. Mock Infected Control	0.0104

No V5 Control vs. Scrambled Control	0.9581
No V5 Control vs. Biotin Control	0.6694
No V5 Control vs. No FISH Control	0.0077
No V5 Control vs. Mock Infected Control	0.0358
Scrambled Control vs. Biotin Control	0.9955
Scrambled Control vs. No FISH Control	0.1405
Scrambled Control vs. Mock Infected Control	0.3601
Biotin Control vs. No FISH Control	0.4685
Biotin Control vs. Mock Infected Control	0.7744
No FISH Control vs. Mock Infected Control	0.9992
M2-1-HA mRNA 8 hpi	
NS1-L vs. No Primary Control	<0.0001
NS1-L vs. No V5 Control	<0.0001
NS1-L vs. Scrambled Control	<0.0001
NS1-L vs. Biotin Control	<0.0001
NS1-L vs. No FISH Control	0.9987
NS1-L vs. Mock Infected Control	0.9998
No Primary Control vs. No V5 Control	0.5838
No Primary Control vs. Scrambled Control	0.2441
No Primary Control vs. Biotin Control	0.497
No Primary Control vs. No FISH Control	<0.0001
No Primary Control vs. Mock Infected Control	<0.0001
No V5 Control vs. Scrambled Control	0.998
No V5 Control vs. Biotin Control	>0.9999
No V5 Control vs. No FISH Control	<0.0001
No V5 Control vs. Mock Infected Control	<0.0001
Scrambled Control vs. Biotin Control	0.9995
Scrambled Control vs. No FISH Control	<0.0001
Scrambled Control vs. Mock Infected Control	<0.0001
Biotin Control vs. No FISH Control	<0.0001
Biotin Control vs. Mock Infected Control	<0.0001
No FISH Control vs. Mock Infected Control	0.9733
**Graph Title**	NS1 mRNA-L PLA (Normalized PLA)
Comparison	P Value
M2-2-Myc mRNA 6 hpi	
NS1-L vs. No Primary Control	0.758
NS1-L vs. No V5 Control	0.9895
NS1-L vs. Scrambled Control	0.9998
NS1-L vs. Biotin Control	0.9996
NS1-L vs. No FISH Control	0.6786
No Primary Control vs. No V5 Control	0.9786
No Primary Control vs. Scrambled Control	0.5883
No Primary Control vs. Biotin Control	0.9039
No Primary Control vs. No FISH Control	0.0546
No V5 Control vs. Scrambled Control	0.9491
No V5 Control vs. Biotin Control	0.9996
No V5 Control vs. No FISH Control	0.289
Scrambled Control vs. Biotin Control	0.9926
Scrambled Control vs. No FISH Control	0.8321
Biotin Control vs. No FISH Control	0.4764
M2-2-Myc mRNA 8 hpi	
NS1-L vs. No Primary Control	<0.0001
NS1-L vs. No V5 Control	<0.0001

NS1-L vs. Scrambled Control	<0.0001
NS1-L vs. Biotin Control	<0.0001
NS1-L vs. No FISH Control	<0.0001
No Primary Control vs. No V5 Control	0.1676
No Primary Control vs. Scrambled Control	0.7066
No Primary Control vs. Biotin Control	0.0057
No Primary Control vs. No FISH Control	<0.0001
No V5 Control vs. Scrambled Control	0.9364
No V5 Control vs. Biotin Control	0.8531
No V5 Control vs. No FISH Control	<0.0001
Scrambled Control vs. Biotin Control	0.2864
Scrambled Control vs. No FISH Control	<0.0001
Biotin Control vs. No FISH Control	<0.0001
M2-1-HA mRNA 6 hpi	
NS1-L vs. No Primary Control	<0.0001
NS1-L vs. No V5 Control	<0.0001
NS1-L vs. Scrambled Control	0.0004
NS1-L vs. Biotin Control	0.0001
NS1-L vs. No FISH Control	0.0006
No Primary Control vs. No V5 Control	>0.9999
No Primary Control vs. Scrambled Control	0.989
No Primary Control vs. Biotin Control	0.9994
No Primary Control vs. No FISH Control	0.9772
No V5 Control vs. Scrambled Control	0.9982
No V5 Control vs. Biotin Control	>0.9999
No V5 Control vs. No FISH Control	0.9945
Scrambled Control vs. Biotin Control	0.9998
Scrambled Control vs. No FISH Control	>0.9999
Biotin Control vs. No FISH Control	0.9988
M2-1-HA mRNA 8 hpi	
NS1-L vs. No Primary Control	0.0561
NS1-L vs. No V5 Control	0.4353
NS1-L vs. Scrambled Control	0.456
NS1-L vs. Biotin Control	0.9455
NS1-L vs. No FISH Control	0.3035
No Primary Control vs. No V5 Control	0.9279
No Primary Control vs. Scrambled Control	0.9178
No Primary Control vs. Biotin Control	0.3953
No Primary Control vs. No FISH Control	<0.0001
No V5 Control vs. Scrambled Control	>0.9999
No V5 Control vs. Biotin Control	0.9347
No V5 Control vs. No FISH Control	0.0014
Scrambled Control vs. Biotin Control	0.9434
Scrambled Control vs. No FISH Control	0.0015
Biotin Control vs. No FISH Control	0.0357
